# Effectiveness of electroacupuncture and acupuncture in alleviating cold hypersensitivity in the hands and feet: A randomized controlled trial

**DOI:** 10.1371/journal.pone.0313789

**Published:** 2024-11-13

**Authors:** Na-Yoen Kwon, Jun-Sang Yu, Dong-Il Kim, Hyeong-Jun Kim, Dong-Nyung Lee

**Affiliations:** 1 Department of Obstetrics and Gynecology, College of Korean Medicine, Ga-Chon University, Seongnam-si, Republic of Korea; 2 Department of Sasang Constitutional Medicine, College of Korean Medicine, Sang-Ji University & Research Institute of Korean Medicine, Sangji University, Wonju, Republic of Korea; 3 Department of Korean Obstetrics and Gynecology, Dongguk University Ilsan Oriental Hospital, Goyang, Republic of Korea; 4 Department of Oriental Gynecology, Jecheon Oriental Hospital of Semyung University, Jecheon, Republic of Korea; 5 Dongnam Esoo Korean Medical Clinic, Cheongju-si, Republic of Korea; Federal University of Rio Grande do Sul: Universidade Federal do Rio Grande do Sul, BRAZIL

## Abstract

**Objective:**

Cold hypersensitivity in the hands and feet(CHHF) is a common condition that reduces the quality of life and causes daily discomfort. The current treatments are primarily pharmacological. This study aimed to expand treatment options by comparing the efficacy of electroacupuncture (EA) and acupuncture (AC) with that of no treatment (control).

**Methods:**

A three-group randomized controlled trial was conducted with 72 women diagnosed with cold hypersensitivity in the hands and feet, as confirmed by subjective symptoms and objective temperature differences. Participants were randomly assigned to the EA, AC, or control groups. Outcome measures included hand and feet visual analog scale (VAS) scores, temperature changes measured using a non-contact thermometer, and World Health Organization Quality of Life-BREF (WHOQOL-BREF) scores assessed at pretreatment (T0), posttreatment (T1), and follow-up (T2). Repeated measures ANOVA and 2-way mixed-model ANOVA were used to evaluate group, time, and interaction effects.

**Results:**

Both the EA and AC groups showed significant improvements in hand and feet VAS and WHOQOL-BREF scores compared with those of the control group posttreatment (T1). Notably, the EA group demonstrated sustained benefits at follow-up (T2), with significant reductions in feet VAS scores and positive changes in several WHOQOL-BREF domains. Interaction effects between group and time were observed, indicating that the changes in the EA and AC groups were meaningfully different form those in the control group. The control group also exhibited a statistically significant reduction in the VAS scores at follow-up (T2), likely due to the natural variability of cold extremity symptoms and psychological factors.

**Conclusion:**

This study demonstrated that EA and AC are effective in alleviating the symptoms of CHHF and enhancing the quality of life compared to no treatment. EA showed long-lasting effects than those of AC, suggesting its potential to regulate the autonomic nervous system. These findings provide a foundation for expanding non-pharmacological treatment options for CHHF and offer clinical guidance on the use of EA and AC.

## Introduction

Cold hypersensitivity in the hands and feet (CHHF), also known as hyperesthesia, is a condition characterized by abnormal sensitivity to temperatures that typically do not cause discomfort [1]. This hypersensitivity can significantly impair daily activities because individuals with CHHF are particularly vulnerable to seasonal, climatic, and environmental changes [2]. These sensitivities often delay the restoration of normal body temperature, limiting both outdoor and indoor activities. Consequently, CHHF can adversely affect the quality of life from both economic and personal perspectives, posing substantial challenges in maintaining normal social and professional interactions.

The primary pathophysiology of CHHF is believed to be a reduction in cutaneous blood flow [3]. CHHF can be caused by Raynaud’s disease, rheumatic disease, hypothyroidism, or cardiovascular disease. In addition, CHHF can occur idiopathically without an identifiable cause. These idiopathic cases are often associated with chronic conditions, including lower extremity edema, fatigue, dyspepsia, and infertility [4, 5].

Interestingly, CHHF is predominantly observed in women, suggesting a potential sex disparity in susceptibility to this condition [6]. Epidemiological studies have indicated that hormonal differences, particularly in estrogen levels, may influence vascular reactivity and pain perception, contributing to a higher prevalence of CHHF among women [7]. This sex-specific prevalence underscores the need for targeted therapeutic strategies that consider physiological differences between men and women.

Acupuncture (AC) has been used in Eastern medicine to treat various ailments, including musculoskeletal pain, mental health disorders, respiratory diseases, and gynecological diseases. In Eastern medicine, AC is understood to regulate the imbalance of yin and yang, whereas in Western medicine, it appears to work on both the peripheral and central nervous systems, balancing the sympathetic and parasympathetic nervous systems [8, 9]. Electroacupuncture (EA), a modern adaptation involving mild electrical currents applied through AC needles, enhances therapeutic effects and is employed in various conditions similar to traditional AC.

Previous randomized controlled trials addressing CHHF have explored treatments, such as Onkyeong-tang, red ginseng, capsinoids, and fermented green tea [1, 10–12]. In Korea, clinical practice guidelines for CHHF in traditional Korean medicine have been published, offering graded recommendations (Grade A or B) for various interventions, including herbal medicine, AC, EA, warm needling, pharmacuacupuncture, chuna therapy, and herbal medicine steaming and washing treatment. These treatments have been rated as ‘should be considered’ for CHHF management [13].

However, despite these established recommendations, there remains a notable gap in the literature concerning direct interventions using AC in patients with CHHF. Previous research has mainly focused on herbal medicine and while acupuncture has been widely used in cilinical practice, its comparative efficacy, especially in relation to electroacupuncture, has not been rigorously tested.

A related study assessed the effects of AC along with transcutaneous electrical nerve stimulation on cold pressor tolerance in healthy individuals and revealed significant posttreatment improvements [14, 15]. Building on these preliminary insights, the objective of this study was to rigorously evaluate the efficacy of both AC and EA in alleviating the symptoms of CHHF, with the aim of substantiating and expanding upon the existing body of research. Therefore, this study includes a no-treatment control group to account for the natural variability of symptoms and placebo effects, and compare it with AC and EA groups to evaluate if EA provides superior outcomes due to enhanced physiolgical responses.

## Method

### Study design

This multicenter, randomized, controlled, three-arm clinical trial recruited participants from 30/10/2019 to 26/09/2020. It was conducted at Sangji University Hospital, Dongguk University Ilsan Hospital, and Semyung University Chungju Hospital in accordance with the guidelines of the Declaration of Helsinki. Ethics approval was granted by the independent ethics committees of Sangji University, Dongguk University, and Semyung University. This study was registered in advance at https://cris.nih.go.kr/cris/index/index.do (number KCT0004306). The purpose and methods of the study were explained to the participants, their written consent was obtained, and they were informed that they could withdraw at any time.

### Participants

The study participants were women between the ages of 19 and 59 years who experienced cold hands and feet, were uncomfortable in their daily activities, felt cold at temperatures where they should not normally feel cold, felt excessively cold compared to others at temperatures where they should normally feel cold, or did not recover easily from coldness when moving from a cold to a warm environment.

#### Inclusion criteria

Participants were recruited from hospital visits where their upper and lower extremities were assessed using a non-contact thermometer (Testo 835-T1, Lenzkirch, Germany) after being exposed to a temperature of 24(±2)°C for 10 min. Candidates were initially considered for inclusion if they exhibited a temperature differential of at least 0.3°C between the arm (LU4) and palm (PC8), or a differential of 2.0°C or more between the anterior thigh (ST32) and dorsum of the foot (LR3). Blood tests were conducted after a preliminary selection. Final inclusion was contingent upon normal blood test results, voluntary participation, and the signing of an informed consent form after the candidates were fully briefed on the study’s aims and procedures.

#### Exclusion criteria

Participants were excluded from the study under any of the following conditions: presence of medical conditions known to cause cold feet other than CHHF; current use of calcium antagonists or beta-blockers for cold foot treatment; diagnosis of heart disease or diabetes; history of substance abuse; diagnosed psychiatric disorders; potential for pregnancy or current pregnancy plans, unless they agreed to use reliable contraception methods throughout the study duration.

### Sample size

The number of participants in this study was determined based on previous randomized trials [16] on CHHF. In the referenced trial, the mean visual analog scale (VAS) score change in the control group was -0.48 with a standard deviation of 1.03, and the mean VAS score change in the treatment group was -1.52 with a standard deviation of 1.17. Based on these values, the expected difference in VAS score change with treatment was calculated to be 1.04. Using the pooled standard deviation formula, the standard deviation was estimated to be 1.102.

However, to ensure the clinical relevance of this sample size estimation, the effect size was chosen to reflect a clinically meaningful difference in CHHF symptoms rather than purely statistical significance. Given the chronic and idiopathic nature of CHHF, an effect size that demonstrated meaningful symptom improvement in daily life was prioritized. This was validated by consulting prior trials in related fields.

The significance level (α), the probability of making a type I error, was set at 0.05, and the probability of making a type II error (β) was assumed to be 0.2, resulting in a power (1-β) of 80%. The number of participants in the EA, AC, and control groups was set to be equal, and the formula for estimating the number of participants by comparing the means of the three groups is as follows: N = 2(Z_α/2_+Z_β_)^2^σ^2^/(μ_c_-μ_t_)^2^.

Based on these calculations, 18 participants were required in each group. Considering a 20% dropout rate, 24 participants were enrolled in each group. Accordingly, this study was designed to enroll 72 participants, assigning 24 participants each to the EA, AC, and control groups for analysis.

### Randomization

In this study, randomization was performed using a sealed letter envelope method. After participants provided informed consent and passed the screening process, they were assigned a randomization code generated by an independent statistician. The statistician provided the randomization codes to each site representative in sealed opaque envelopes, which were opened sequentially based on the order of screening completion to ensure allocation concealment. Particiapnts were randomized in a 1:1:1 ratio into three groups: acupuncture treatment (AC), electroacupuncture treatment (EA), and no-treatment (Control). The block randomization method was applied using the blockrand package in R version 3.4.2 or later, with stratification by site to balance participants allocation across groups.

Randomization ensured that the assessors evaluating the participants’ symptoms were blinded to the treatment groups. However, the practitioners administering the acupuncture and electroacupuncture treatments were not blinded, as their role required knowledge of the intervention type. To minimize potential bias, the assessors were blinded to the group assignments and were responsible for evaluating the outcomes independently from the practitiones.

### Treatments

Following randomization, the untreated group was sent home without intervention. Participants in the EA and AC groups received treatment twice weekly for a total duration of five weeks, resulting in ten treatments. For AC, 0.20 mm gauge and 30 mm long stainless-steel needles (manufactured by Dongbang Medical, Boryeong, Korea) were used. These needles were inserted subcutaneously to depths ranging from 10 to 25 mm at selected AC points, including bilateral TE5, LI4, LR3, and SP6, and were left in place for 15 min to allow qi flow.

The EA group underwent a similar procedure. However, they also received additional stimulation using a low-frequency stimulator (STN-330, Stratek, Republic of Korea), which was connected between pairs of AC points TE5-LI4 and LR3-SP6. A dipolar symmetrical wave set at 2 Hz was applied for 15 min at the highest intensity tolerated by the participants without pain. All AC treatments were administered by practitioners licensed in Korean medicine, each with a minimum of five years of clinical experience.

### Outcome

The primary efficacy outcome of this study was assessed using the VAS for CHHF symptoms. This tool was previously used in a randomized trial to investigate the effects of red ginseng on CHHF. During each study visit, participants were asked to rate their level of coldness on a VAS, which consisted of a horizontal line labeled with numbers ranging from 0 to 10. The left end of the line was marked "no cold," indicating no symptoms, whereas the right end was labeled "the most severe cold imaginable," representing extreme discomfort. The participants selected the number that best corresponded to their current level of coldness, which was recorded as their score. Participants were evaluated at three key time points throughout the study: at baseline (T0), which refers to the time point immediately before randomization; post-treatment (T1), which was measured immediately after the 5-week treatment period (5 weeks ± 3days after randomization); and follow-up (T2), conducted 4 weeks after the conclusion of the treatment period (9 weeks ± 3 days after randomization).

The secondary efficacy outcome of this study was the body surface temperatures of the hands and feet. At each visit in the study, the participants’ upper and lower extremities were exposed to a room temperature of 24 (±2)°C for 10 min. Subsequently, body surface temperatures were measured bilaterally on the arm (LU4), palm (PC8), anterior thigh (ST32), and dorsum of the foot (LR3) using a non-contact thermometer. During the screening visit, only the left side was measured to exclude changes in the body surface temperature due to the participant’s activity level. After enrollment, measurements were performed bilaterally. The assessment was conducted by calculating the average temperature difference between the bilateral LU4 and PC8 points and the average temperature difference between the bilateral ST32 and LR3 points.

Another secondary efficacy outcome focused on assessing quality of life. The patients completed the questionnaires at three key points: immediately before randomization, immediately after the intervention was completed, and during the final follow-up visit. The questionnaire was the World Health Organization Quality of Life-BREF (WHOQOL-BREF) scale, which covers various domains, including overall quality of life and health status, physical health, psychological health, social relationships, and environmental condition. Each domain was scored independently by summing the scores of all items within that domain, averaging them, and multiplying by four to obtain the domain score. This method ensured a comprehensive evaluation of the impact of the treatments on different aspects of the participants’ lives.

Safety assessments were conducted by monitoring vital signs and conducting laboratory tests. At each visit, vital signs (blood pressure, pulse, and temperature) were measured. Additionally, comprehensive hematology tests (including white blood cells, red blood cells, hemoglobin, and platelets) and blood biochemistry panels (covering blood urea nitrogen, creatinine, aspartate aminotransferase, alanine aminotransferase, and gamma-glutamyl transpeptidase) were performed during the initial screening (visit 0) and at the conclusion of the intervention (visit 10). The investigators closely monitored the test results, along with any reported adverse events (AEs), to evaluate the safety of the intervention. AEs were diligently recorded at each visit, with participants being advised that they could withdraw from the study at any point if they chose to do so.

### Statistical analysis

Statistical analyses of the efficacy endpoints were conducted using intention-to-treat analysis. To evaluate differences between groups, as well as changes across visits within groups, a combination of 2-way mixed-model analysis of variance (ANOVA) and repeated measures ANOVA was employed. Specifically, the mixed-model ANOVA was used to assess the interaction effects between group (EA, AC, and Control) and time (T0, T1, T2). Repeated measures ANOVA was performed to evaluate changes within each group across time points, and paired t-tests were used for post hoc comparisons.

Furthermore, the differences between posttreatment and pretreatment values, as well as between follow-up and pretreatment values, were calculated to obtain Cohen’s effect size. Confidence intervals were calculated to determine the differences between groups. For all mulitple hypothesis testing, a correction for multiple comparisons using the Bonferroni method was applied to control for Type Ⅰ error inflation, ensuring that significant findings across multiple time points and variables were robust and not due to chance.

For safety endpoints, appropriate statistical tests, such as ANOVA, paired t-tests, chi-squared tests, and Fisher’s exact tests, were selected based on the type of data being analyzed. The last observation carried forward (LOCF) method was used to address missing values. This approach substitutes missing data points for efficacy endpoints with the last available observation. However, safety endpoints were treated as missing data. All statistical analyses were performed using R version 3.6.1, and SPSS for Windows version 23.0. A p-value of 0.05 or lower was predetermined as the threshold for statistical significance.

## Results

### Participants’ characteristics

**Fig 1:**
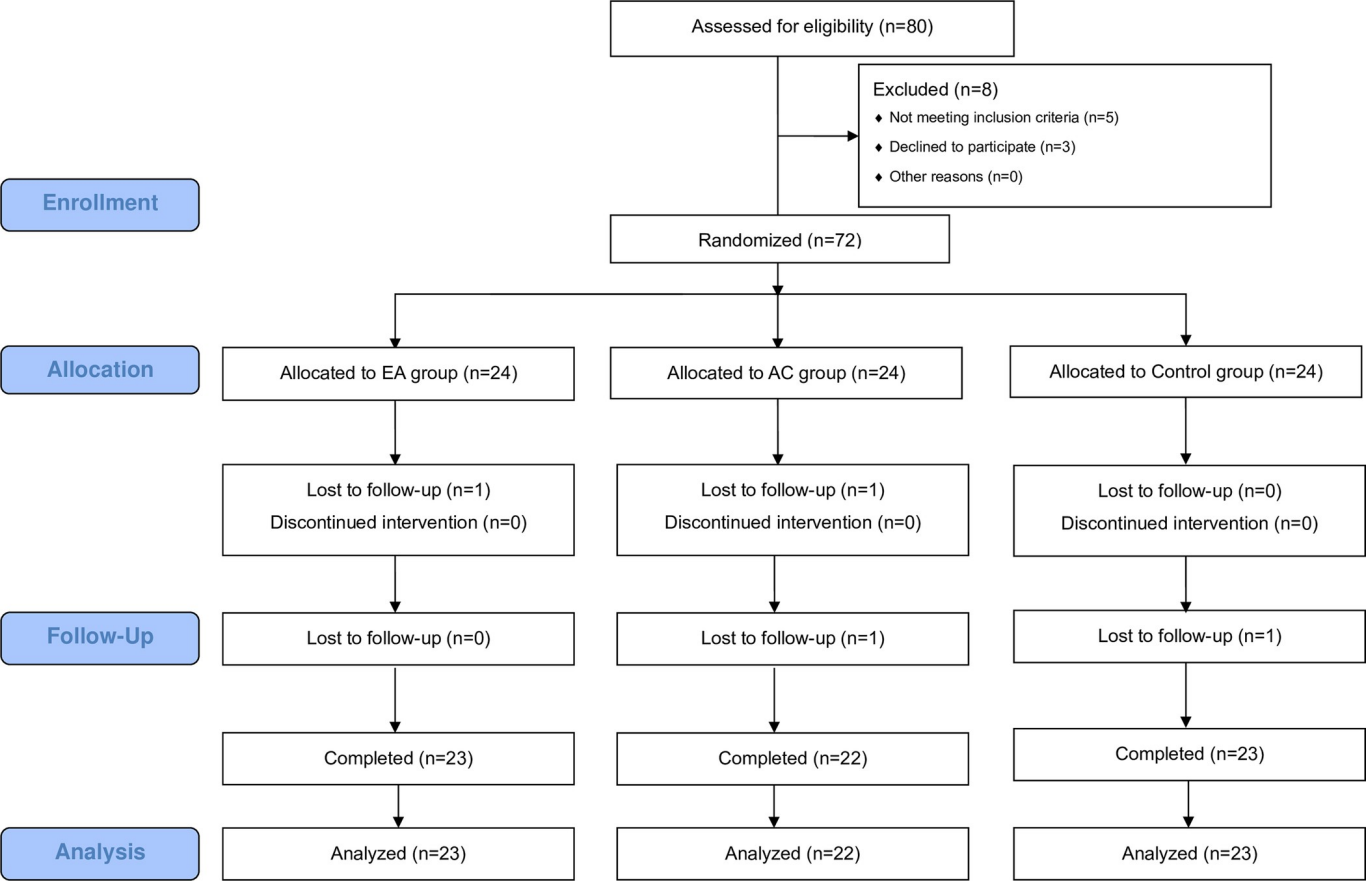
Flow chart of the participants. EA, electroacupuncture; AC, acupuncture.

Eighty participants were initially enrolled in the study, which was conducted across three hospitals. Of these, 72 participants who met the inclusion criteria were randomly assigned to one of three groups: EA, AC, or control. During the course of the study, one patient from the EA group, two from the AC group, and one from the control group dropped out. This resulted in a final sample size of 68 participants who completed the study. The trial flow of the participants is shown in Fig 1.

### Between-group comparison

The demographic and clinical characteristics of the 72 participants included in the trial are shown in Table 1. A one-way ANOVA was conducted for the three groups at each time point. No statistically significant differences were observed in demographic factors such as age, smoking status, alcohol consumption, and exercise habits between the groups. However, a ststistically significant differecne was found in the baseline hand coldness VAS scores. As this score is one of the primary outcome measures, this basline difference was taken into account during the interpretation of treatment outcome. The results indicated significant differences in the coldness VAS scores of the hands and feet immediately after treatment (T1) and at follow-up (T2), as well as in the temperature difference of the feet at follow-up (T2). Additionally, significant differences were found in all domains of the WHOQOL-BREF except for the social domain at follow-up (T2).

**Table 1:** Participants’ characteristics.

	EA group	AC group	Control group	p-value
	(N = 24)	(N = 24)	(N = 24)	
Demographic characteristics				
Age (years)	35.3 ± 12.0	38.3 ± 13.9	42.2 ± 12.2	0.274
Height (cm)	160.0 ± 5.37	158.0 ± 6.79	159.0 ± 4.98	0.900
Weight (kg)	54.5 ± 8.57	54.0 ± 5.77	51.5 ± 5.29	0.189
BMI (kg/m^2^)	21.3 ± 2.89	21.5 ± 1.66	20.4 ± 2.18	0.135
Exercise (%)	62.5	79.2	54.2	0.180
Smoking (%)	0	4.2	0	0.391
Alcohol (%)	29.2	33.3	25.0	0.817
Clinical characteristics				
Hand VAS score (T0)	5.83 ± 1.05	6.21 ± 1.38	7.08 ± 1.25	0.003
Hand VAS score (T1)	4.08 ± 1.67	4.33 ± 1.63	6.75 ± 1.39	<0.001
Hand VAS score (T2)	4.42 ± 1.84	4.71 ± 2.10	6.13 ± 1.68	0.005
Feet VAS score (T0)	6.79 ± 1.02	7.17 ± 1.40	7.46 ± 1.14	0.163
Feet VAS score (T1)	5.00 ± 1.82	5.67 ± 1.79	7.13 ± 1.36	<0.001
Feet VAS score (T2)	4.50 ± 1.69	5.29 ± 2.10	6.46 ± 1.61	0.002
ΔT _hands_ (°C) (T0)	1.56 ± 1.42	1.25 ± 1.26	2.30 ± 1.98	0.068
ΔT _hands_ (°C) (T1)	0.70 ± 1.49	0.67 ± 1.97	1.51 ± 2.80	0.319
ΔT _hands_ (°C) (T2)	0.38 ± 2.17	0.90 ± 1.81	1.34 ± 2.36	0.299
ΔT _feet_ (°C) (T0)	2.31 ± 2.41	2.99 ± 1.58	2.54 ± 1.95	0.489
ΔT _feet_ (°C) (T1)	2.72 ± 2.26	2.25 ± 2.30	2.34 ± 2.16	0.745
ΔT _feet_ (°C) (T2)	0.84 ± 1.75	2.38 ± 1.74	2.22 ± 2.11	0.011
WHOQOL-BREF Total score (T0)	79.33 ± 7.84	78.75 ± 9.03	75.96 ± 8.46	0.341
WHOQOL-BREF Total score (T1)	88.83 ± 8.81	87.29 ± 10.82	82.83 ± 9.16	0.089
WHOQOL-BREF Total score (T2)	91.00 ± 10.84	86.96 ± 10.83	81.21 ± 11.10	0.011
WHOQOL-BREF Physical domain (T0)	23.67 ± 2.96	23.63 ± 3.03	22.67 ± 3.25	0.450
WHOQOL-BREF Physical domain (T1)	24.50 ± 3.22	24.54 ± 3.31	23.17 ± 3.17	0.252
WHOQOL-BREF Physical domain (T2)	25.54 ± 3.71	24.21 ± 3.19	22.63 ± 3.99	0.026
WHOQOL-BREF Psychological domain (T0)	18.92 ± 2.99	19.00 ± 3.11	18.50 ± 2.62	0.817
WHOQOL-BREF Psychological domain (T1)	19.83 ± 2.82	19.46 ± 2.75	18.46 ± 2.34	0.185
WHOQOL-BREF Psychological domain (T2)	20.75 ± 2.92	19.33 ± 2.82	22.63 ± 3.99	0.015
WHOQOL-BREF Social domain (T0)	10.54 ± 1.25	10.08 ± 1.59	10.21 ± 1.28	0.497
WHOQOL-BREF Social domain (T1)	10.33 ± 1.09	10.33 ± 1.49	10.21 ± 1.47	0.935
WHOQOL-BREF Social domain (T2)	10.42 ± 1.50	10.13 ± 1.62	10.04 ± 1.33	0.660
WHOQOL-BREF Environmental domain (T0)	26.21 ± 3.12	26.04 ± 3.26	24.58 ± 2.99	0.148
WHOQOL-BREF Environmental domain (T1)	27.42 ± 3.28	26.21 ± 3.95	24.63 ± 2.89	0.021
WHOQOL-BREF Environmental domain (T2)	27.54 ± 3.79	26.42 ± 4.05	23.83 ± 3.37	0.003

EA, electroacupuncture; AC, acupuncture; VAS, visual analog scale; BMI, body mass index; WHOQOL-BREF, World Health Organization Quality of Life-BREF

Repeated measures ANOVA was conducted to compare group differences over time (Table 2). The results indicated significant between-group differences in the coldness VAS scores for both hands and across time points. However, no significant differences were found for the temperature difference in hands and feet. Regarding the WHOQOL-BREF domains, the environmental domain showed significant group differences, while no significant differences were found in other domains.

**Table 2:** Repeated measures ANOVA results between group comparison.

	df	SS	MS	F	p-value
Hand VAS score	2	145.731	72.866	17.897	0.000
Feet VAS score	2	91.815	45.907	9.574	0.000
ΔT _hands_ (°C)	2	31.468	15.734	2.079	0.133
ΔT _feet_ (°C)	2	12.977	6.489	0.955	0.390
WHOQOL-BREF Total score	2	1531.704	765.852	3.095	0.052
WHOQOL-BREF Physical domain	2	119.148	59.574	2.160	0.123
WHOQOL-BREF Psychological domain	2	76.231	38.116	1.931	0.153
WHOQOL-BREF Social domain	2	3.370	1.685	0.345	0.709
WHOQOL-BREF Environmental domain	2	277.083	138.542	4.733	0.012

### Within-group comparison

A repeated measures ANOVA was performed to assess changes over time (T0, T1, T2) within and between the groups for all outcome measures (Table 3). Significant group-by-time interactions were observed in the hand VAS scores and feet VAS scores, indicating that the effects of EA and AC treatments on cold hypersensitivity differed significantly from the control group across time points. The feet temperature difference also showed a significant group-by-time interaction, further supporting the efficacy of EA and AC in modulating temperature regulation in the feet compared to the control group.

**Table 3:** Repeated measures ANOVA results (within-group comparison).

		df	SS	MS	F	p-value
Hand VAS score	Time	2	81.843	40.921	23.800	<0.001
	Time x Group	4	18.213	4.553	2.648	0.036
Feet VAS score	Time	2	112.565	56.282	41.473	<0.001
	Time x Group	4	16.824	4.206	3.099	0.018
ΔT _hands_ (°C)	Time	2	30.096	15.048	7.325	0.001
	Time x Group	4	4.520	1.130	0.550	0.699
ΔT _feet_ (°C)	Time	2	25.428	12.714	4.393	0.014
	Time x Group	4	30.064	7.516	2.597	0.039
WHOQOL-BREF Total score	Time	1.842	3339.065	1812.707	92.199	<0.001
	Time x Group	3.684	252.713	68.596	3.489	0.012
WHOQOL-BREF Physical domain	Time	2	29.148	14.574	5.153	0.007
	Time x Group	4	27.907	6.977	2.467	0.048
WHOQOL-BREF Psychological domain	Time	2	14.898	7.449	3.369	0.037
	Time x Group	4	29.324	7.331	3.316	0.013
WHOQOL-BREF Social domain	Time	2	0.398	0.199	0.358	0.700
	Time x Group	4	1.435	0.359	0.644	0.632
WHOQOL-BREF Environmental domain	Time	1.794	8.361	4.661	1.369	0.258
	Time x Group	3.588	28.889	8.052	2.365	0.063

For the WHOQOL-BREF total score, a significant interaction between time and group was found, with notable improvements in quality of life in the EA and AC groups compared to the control group. Significant interactions were also observed in the psychological domain and physical domain, indicating that the EA and AC treatments had a sustained positive impact on psychological and physical well-being over time.

Paired t-tests were conducted to compare the differences between pretreatment (T0) and posttreatment (T1) values, as well as pretreatment (T0) and follow-up (T2) values within each group (Table 4). The results showed that both the EA group and AC group exhibited significant improvements in various variables compared with those of their pretreatment values, whereas the control group did not show any significant effects. Specifically, in the treatment groups, the cold VAS scores were significantly lower, whereas the temperature and quality of life scores were significantly higher. In the EA group, these significant differences were maintained even at follow-up (T2).

**Table 4:** Change scores at T1 and T2.

		EA group	AC group	Control group
		(N = 24)	(N = 24)	(N = 24)
Change score (T1-T0)				
Hand VAS score	Mean difference (95% CI)	-1.75 (-2.48, -1.02)	-1.88 (-2.66, -1.09)	-0.33 (-0.93, 0.26)
	p-value	<0.001	<0.001	0.257
Feet VAS score	Mean difference (95% CI)	-1.79 (-2.43, -1.16)	-1.50 (-2.27, -0.73)	-0.33 (-0.78, 0.11)
	p-value	<0.001	<0.001	0.133
ΔT _hands_ (°C)	Mean difference (95% CI)	-0.86 (-1.53, -0.19)	-0.58 (-1.20, 0.05)	-0.80 (-1.86, 0.27)
	p-value	0.014	0.068	0.137
ΔT _feet_ (°C)	Mean difference (95% CI)	0.41 (-0.83, 1.65)	-0.74 (-1.67, 0.18)	-0.20 (-1.11, 0.70)
	p-value	0.500	0.110	0.645
WHOQOL-BREF Total score	Mean difference (95% CI)	9.50 (7.58, 11.42)	8.54 (6.51, 10.57)	6.88 (4.17, 9.58)
	p-value	0.000	0.000	0.000
WHOQOL-BREF Physical domain	Mean difference (95% CI)	0.83 (-0.14, 1.80)	0.92 (0.19, 1.64)	0.50 (-0.41, 1.41)
	p-value	0.089	0.015	0.266
WHOQOL-BREF Psychological domain	Mean difference (95% CI)	0.92 (0.06, 1.77)	0.46 (-0.29, 1.21)	-0.04 (-1.00, 0.92)
	p-value	0.036	0.217	0.929
WHOQOL-BREF Social domain	Mean difference (95% CI)	-0.21 (-0.66, 0.24)	0.25 (-0.20, 0.70)	0.00 (-0.41, 0.41)
	p-value	0.347	0.266	1.000
WHOQOL-BREF Environmental domain	Mean difference (95% CI)	1.21 (0.34, 2.08)	0.17 (-1.05, 1.38)	0.04 (-0.93, 1.02)
	p-value	0.009	0.780	0.930
Change score (T2-T0)				
Hand VAS score	Mean difference (95% CI)	-1.42 (-2.18, -0.65)	-1.50 (-2.39, -0.61)	-0.96 (-1.78, -0.14)
	p-value	0.001	0.002	0.024
Feet VAS score	Mean difference (95% CI)	-2.29 (-2.94, -1.64)	-1.88 (-2.78, -0.97)	-1.00 (-1.66, -0.34)
	p-value	0.000	0.000	0.005
ΔT _hands_ (°C)	Mean difference (95% CI)	-1.18 (-2.03, -0.33)	-0.74 (-1.67, 0.18)	-0.96 (-2.07, 0.15)
	p-value	0.009	0.110	0.087
ΔT _feet_ (°C)	Mean difference (95% CI)	-1.46 (-2.77, -0.16)	-0.61 (-1.24, 0.01)	-0.32 (-1.35, 0.70)
	p-value	0.029	0.054	0.516
WHOQOL-BREF Total score	Mean difference (95% CI)	11.67 (8.91, 14.42)	8.21 (5.38, 11.04)	5.25 (2.23, 8.27)
	p-value	0.000	0.000	0.002
WHOQOL-BREF Physical domain	Mean difference (95% CI)	1.88 (0.86, 2.89)	0.58 (-0.40, 1.56)	-0.04 (-1.33, 1.25)
	p-value	0.001	0.231	0.947
WHOQOL-BREF Psychological domain	Mean difference (95% CI)	1.83 (0.90, 2.76)	0.33 (-0.64, 1.31)	-0.29 (-1.25, 0.67)
	p-value	0.000	0.488	0.536
WHOQOL-BREF Social domain	Mean difference (95% CI)	-0.13 (-0.61, 0.36)	0.04 (-0.54, 0.62)	-0.17 (-0.53, 0.20)
	p-value	0.601	0.883	0.357
WHOQOL-BREF Environmental domain	Mean difference (95% CI)	1.33 (0.23, 2.44)	0.38 (-0.97, 1.72)	-0.75 (-1.87, 0.37)
	p-value	0.020	0.568	0.178

EA, electroacupuncture; AC, acupuncture; VAS, visual analog scale; CI, confidence interval; WHOQOL-BREF, World Health Organization Quality of Life-BREF

### Effect sizes

Table 5 shows the effect sizes of the comparisons among the EA, AC, and control groups. Immediately after treatment (T1), both the EA and AC groups had significantly negative effect sizes in the hand and feet VAS scores compared with those of the control group. At follow-up (T2), the EA group continued to show significantly negative effect sizes in the feet VAS scores and positive effect sizes in several WHOQOL-BREF domains compared with those of the control group. Additionally, significant differences were observed between the EA and AC groups.

**Table 5:** Effect size.

Effect size	T1-T0			T2-T0		
	EA vs. Control	AC vs. Control	EA vs. AC	EA vs. Control	AC vs. Control	EA vs. AC
Hand VAS score	-0.89 (-1.48, -0.29)	-0.93 (-1.52, -0.33)	0.07 (-0.49, 0.64)	-0.24 (-0.81, 0.33)	-0.26 (-0.83, 0.31)	0.04 (-0.53, 0.61)
Feet VAS score	-1.11 (-1.72, -0.50)	-0.77 (-1.36, -0.19)	-0.17 (-0.74, 0.40)	-0.82 (-1.40, -0.23)	-0.46 (-1.03, 0.11)	-0.22 (0.78, 0.35)
ΔT _hands_ (°C)	-0.03 (-0.59, 0.54)	0.10 (-0.46, 0.67)	-0.18 (-0.75, 0.39)	-0.09 (-0.66, 0.47)	0.28 (-0.29, 0.84)	-0.45 (-1.02, 0.12)
ΔT _feet_ (°C)	0.23 (-0.33, 0.80)	-0.25 (-0.81, 0.32)	0.44 (-0.14, 1.01)	-0.40 (-0.98, 0.17)	-0.14 (-0.71, 0.42)	-0.35 (-0.92, 0.22)
WHOQOL-BREF Total score	0.46 (-0.11, 1.04)	0.29 (-0.28, 0.86)	0.20 (-0.37, 0.77)	0.92 (0.33, 1.52)	0.42 (-0.15, 0.99)	0.51 (-0.06, 1.09)
WHOQOL-BREF Physical domain	0.15 (-0.42, 0.71)	0.21 (-0.36, 0.78)	-0.04 (-0.61, 0.52)	0.69 (0.10, 1.27)	0.22 (-0.34, 0.79)	0.54 (-0.03, 1.12)
WHOQOL-BREF Psychological domain	0.44 (-0.13, 1.01)	0.24 (-0.33, 0.81)	0.24 (-0.33, 0.81)	0.93 (0.34, 1.53)	0.27 (-0.30, 0.83)	0.65 (0.07, 1.23)
WHOQOL-BREF Social domain	-0.20 (-0.77, 0.36)	0.24 (-0.33, 0.81)	-0.42 (-1.00, 0.15)	0.04 (-0.53, 0.60)	0.18 (-0.39, 0.75)	-0.13 (-0.70, 0.43)
WHOQOL-BREF Environmental domain	0.53 (-0.05, 1.10)	0.05 (-0.52, 0.61)	0.41 (-0.16, 0.98)	0.78 (0.19, 1.36)	0.38 (-0.19, 0.95)	0.32 (-0.25, 0.89)

EA, electroacupuncture; AC, acupuncture; VAS, visual analog scale; WHOQOL-BREF, World Health Organization Quality of Life-BREF

### AEs

There were six AEs in this study: three in the EA group, two in the AC group, and one in the control group. The reported AEs included tonsillitis, knee pain, corn, and runny nose, all of which were mild and none were serious. No statistically significant differences in the rate of AEs were observed among the groups. Laboratory test results were all within normal limits, and no abnormal values were identified as a result of the intervention.

## Discussion

This study was a three-group randomized controlled trial comparing the efficacy and safety of EA, AC, and no treatment (control) in women with CHHF. The inclusion criteria were stringent and required participants to exhibit both subjective symptoms and objective temperature measurements of the cold extremities. Despite these strict criteria, 72 participants were successfully recruited and randomly assigned to one of three groups. The dropout rate was low, with only four participants (5.56%) withdrawing, and adherence to the treatment protocol was high among the remaining participants.

Within-group comparisons between posttreatment (T1) and pretreatment (T0), as well as between follow-up (T2) and pretreatment (T0), showed a tendency to alleviate cold symptoms and improve quality of life across all groups, including the control group. However, statistically significant improvements were observed primarily in both EA and AC groups. Interestingly, the control group also exhibited a statistically significant reduction in VAS scores at follow-up (T2) despite not receiving any intervention. This improvement could be attributed to the natural variability in cold extremity symptoms, which are highly influenced by changes in stress and lifestyle [17]. However, the improvements seen in the control group were limited to the natural course of symptom fluctuations, likely influenced by psychological factors and the placebo effect, rather than specific physiological changes induced by an intervention.

On the other hand, the EA and AC groups showed significantly greater improvements in VAS scores and quality of life measures compared to the control group, even when accounting for these same psychological and lifestyle variables. The 2-way mixed-model ANOVA results demonstrated that significant interaction effects between group and time were present, highlighting that the observed changes over time in the EA and AC groups were meaningfully different from the control group. This suggests that while all groups may have been subject to stress and lifestyle fluctuations, the physiological effects of EA and AC, such as improved autonomic regulation and increased microcirculation, contributed to the additional benefits seen in the treatment groups.The effect sizes of the comparisons among the EA, AC, and control groups revealed that the EA and AC groups had significantly negative effect sizes in hand and feet VAS scores compared with those of the control group immediately posttreatment (T1). At follow-up (T2), the EA group continued to show significantly negative effect sizes in the feet VAS scores and positive effect sizes in several WHOQOL-BREF domains compared with those of the control group. Notably, effective outcomes were observed for the feet VAS scores and the WHOQOL-BREF psychological domain in the EA group compared with those in the control group.

AC point stimulation attempts to normalize the autonomic nervous system under pathological conditions [18]. This is achieved by the release of opioids and neuropeptides from the central nervous system and increased microcirculation to the muscles during low-frequency EA stimulation [19]. Therefore, AC and EA interventions are effective in relieving self-reported cold feet compared to the effect of no treatment, and this effect is thought to be due to changes in autonomic nerve activity [20]. In particular, EA interventions are believed to enhance the efficacy of AC interventions by regulating the autonomic nervous system and the hypothalamic-pituitary-adrenal axis [21]. This study, similar to previous research, demonstrated that AC can alleviate symptoms of cold extremities, a condition associated with autonomic dysregulation, and improve quality of life. By specifically focusing on cold extremities and providing a comparative analysis of EA and traditional AC, this study contributes to the growing body of evidence supporting the efficacy of AC.

Regarding the sample size calculation, the effect size used in this study was derived from a previous randomized trial addressing cold hypersensitivity using oxygen chamber therapy. Although this provided a reasonable basis for estimating participant numbers, we acknowledge that the effect size may not perfectly represent the clinical significance of acupuncture and electroacupuncture for CHHF. We have discussed this limitation in the revised manuscript and believe that while our approach allowed us to conduct a robust and adequately powered study, future research could benefit from more specific data tailored to the particular interventions used in this study. Furthermore, CHHF is often treated with herbal medications in clinical practice, but few RCTs have examined non-pharmacological interventions like acupuncture or electroacupuncture. As such, this study represents an improtant step in evaluating these treatment modalities, potentially serving as a foundation for future clinical trials assessing medical devices and non-drug therapies for CHHF. This could also refine sample size calculations by providing more specific effect size data relevant to these interventions, thereby improving the precision and clinical relevance of future research.

Other limitations include the relatively short follow-up period and reliance on self-reported measures, which may be biased. Although the sample sizewas sufficient to detect significant effects in this study, increasing the sample size in future studies could enhance the generalizability of the results.

The findings of the present study have important clinical implications. EA and AC can be considered effective treatment options for patients with cold extremities, offering a non-pharmacological approach to symptom management. The observed improvements in quality of life further highlight the potential benefits of these treatments in holistic patient care. Clinicians should consider incorporating these modalities into their practice, particularly for patients who are unresponsive to conventional treatments.

Future studies should focus on longer follow-up periods to assess the sustainability of the treatment effects observed in this study. Additionally, larger sample sizes and more diverse patient populations are needed to validate these findings and explore the underlying mechanisms of action in greater detail. Future studies should investigate the specific components of EA and AC that contribute to their efficacy and cost-effectiveness in clinical practice.

## Conclusion

This randomized controlled trial demonstrated that both EA and AC are effective in alleviating symptoms of CHHF and improving the quality of life in women. Notably, the EA group showed sustained benefits at follow-up, underscoring the potential long-term efficacy of EA. The 2-way mixed-model ANOVA confirmed significant interaction effects between time and group, indicating that the observed improvements in the EA and AC groups were not solely due to natural varibaility or phychological factros, but rather the result of physiological changes such as autonomic nervnous system regulation. These findings underscore the utility of EA and AC as non-pharmacological interventions for CHHF, expanding the scope of treatment options in clinical practice. Furthermore, this study provides a foundation for future clinical trials, particularly those exploring the use of non-drug therapies and medical devices in managing cold hypersensitivity, potentially leading to more personilized treatment strategies for patients with CHHF.

## Supporting information

S1 File STRICTA-2010-Checklist_EACHHF. (DOCX)

S2 File CONSORT-2010-Checklist_EACHHF. (DOC)

S3 File Study protocol_EACHHF (Korean). (PDF)

S4 File Study protocol_EACHHF (English). (DOCX)

S5 File (EAACHHF) raw data. (XLSX)
