# Supplementary material for: Effectiveness of electroacupuncture and acupuncture in alleviating cold hypersensitivity in the hands and feet: A randomized controlled trial
Source: PLoS One. 2024 Nov 13;19(11):e0313789. doi: 10.1371/journal.pone.0313789 (PMC11559979; doi:10.1371/journal.pone.0313789)
Supplement: S3 File — (PDF) [file pone.0313789.s003.pdf]

수족냉증에 대한 전침 치료의 유효성 안전성에 대한 다기관 임상연구

Ver 2.1

수족냉증에 대한 전침 치료의
유효성 안전성에 대한 다기관 임상연구
- 무작위배정, 대조군 임상시험-

Protocol Number : Acu 2019_05

연구의뢰처 :	상지대학교 산학협력단
임상시험 총책임자:	유준상
공동 연구 책임자:	이동녕, 김동일
문서유형:	임상시험계획서
작성일:	2019년 5월 15일
Ver:	2.1

수족냉증 한의표준진료지침 개발팀

CONFIDENTIAL
본 임상시험계획서와 관련된 모든 정보는 기밀사항이며,
수족냉증 한의표준진료지침 개발팀의 동의 없이 제 3자에게 공개될 수 없습니다.

〈임상시험 계획서 개요〉

제 목	**수족냉증에 대한 전침 치료의 유효성 안전성에 대한 다기관 임상연구<br>- 무작위배정, 대조군 임상시험-**
목 적	수족냉증 환자에 대한 침치료 또는 전침치료가 수족냉증 완화의 유효성과 안전성을 다기관, 무작위 배정, 대조군 임상시험을 통해 객관적으로 비교 평가하기 위함이다.
임상시험 실시기관	상지대학교 부속한방병원<br>세명대학교 충주한방병원<br>동국대학교 일산한방병원
임상시험 책임자	상지대학교 부속한방병원 유준상 교수<br>세명대학교 충주한방병원 이동녕 교수<br>동국대학교 일산한방병원 김동일 교수
임상시험 의뢰자	의뢰자명 : 상지대학교 산학협력단<br>주 소 : 강원도 원주시 상지대길 80
시험설계	무작위배정, 다기관, 대조군
임상시험 기간	각 기관의 IRB 승인일로부터 24개월
연구 대상자 수	총 **72** 명 (전침치료군 24명, 침치료군 24명, 무처치대조군 24명)<br>상지대학교 부속한방병원: 18명<br>세명대학교 충주한방병원: 24명<br>동국대학교 일산한방병원: 30명
대상질환	수족냉증
임상시험 중재	전침치료군 : 경혈점 자침(좌우 합곡, 외관, 태충 삼음교) + 전침치료<br>침치료군 : 경혈점 자침(좌우 합곡, 외관, 태충 삼음교)<br>무처치대조군

중재기간	침치료군 : 주 2회, 5주간 총 10회, 1회 당 15분 유침<br>전침치료군 : 주 2회, 5주간 총 10회, 1회 당 15분 전침 자극<br>무처치대조군 : Vist 1, Weeks 5, Weeks 9에 방문, 총 3회방문
연구대상자<br>선정기준	1. 수족냉증을 호소하는 만 19세 이상 만 59세 이하의 여성<br>2. 아래 정의에 해당하고, 범위항목 중 한 가지 이상 해당하는 자<br>(1) 정의 : 손과 발이 차갑게 느껴져 불편함을 호소하는 자<br>(2) 범위 항목<br>첫째, 추위를 느끼지 않을 만한 온도에서 수족냉증을 느끼는 경우<br>둘째, 추위를 느낄 만한 온도에서 주변 사람보다 과도하게 수족냉증을 느끼는 경우<br>셋째, 추운 환경에서 따뜻한 환경으로 이동 시 증상이 쉽게 회복되지 않는 경우<br>3. 스크리닝 방문 당시, 수부 또는 족부냉증의 시각적 사상척도(Visual Analogue Scale : VAS)가 4 이상인 자.<br>4. 스크리닝 방문 당시, 상하지부 상온 노출 상태에서 10분간 24℃(±2)의 실온 적응 후 써모미터로 측정시 수장부위(PC8)와 전완부 중심부(LU4)의 온도차가 0.3℃ 이상 또는 전대퇴중심부(ST32)와 족배부 전면부(LR3)의 온도차가 2℃ 이상 낮은 자<br>5. 임상시험 수행 중 추적관찰이 가능한 자<br>6. 본 임상연구의 목적 및 특성에 대한 명백한 설명을 들은 후 참여에 동의하고 동의서에 서명한 자.
연구대상자<br>제외기준	1. 수족냉증 치료 목적으로 칼슘길항제 혹은 베타차단제를 복용하고 있는 자.<br>2. 손가락에 궤양 또는 괴저가 1개 이상 있는 자<br>3. 갑상선기능저하증을 진단받았거나, 갑상선 관련 약물을 복용하는 자<br>4. 자가면역질환을 진단받은자<br>5. 수근관 또는 족근관 증후군을 진단 받았거나, 팔렌 티넬 검사에서 양성 결과 나온 자<br>6. 경추 또는 요추 추간판 탈출증을 진단받은 자<br>7. 당뇨병을 진단 받은 자<br>8. 수부 족부냉증에 영향을 줄 수 있는 약물 복용자 (예, 항응고제 등)<br>9. 중증의 간질환자(AST·ALT 각 100 IU/L 이상) 또는 신부전 환자(Creatinine 2.0mg/dL 이상)<br>10. 행동장애 또는 우울증, 불안신경증 및 심각한 정신장애를 가진 자<br>11. 성인여자(비임신) Hemoglobin 7g/dL 미만, WBC 11,000/mm3 이상,<br>12. 2회 측정하여 평균 수축기 혈압 180mmHg 또는 이완기 혈압 100mmHg 이상<br>13. 심전도 상 치료가 필요한 부정맥이거나, 허혈성심질환 등 심장질환 자<br>14. 알콜이나 약물 남용자<br>15. 임신부(요중 hCG 양성), 수유부, 임신계획이 있거나 적절한 피임방법 선택에 동의하지 않는 가임여성<br>16. 악성종양을 진단받았거나 치료 경험자<br>17. 다른 임상연구에 참가 중이거나 임상시험 종료한지 2개월 이내인 자.<br>18. 임상시험에 참여 혹은 동의서에 제공을 거부한 자<br>19. 한국어를 이해하거나 표현할 능력이 부족한 자<br>20. 시험자의 판단에 의거 임상시험에 부적합하다고 판단된 자

<table>
<tr><td>병용금지
약물/요법</td><td>1. 시험기간 중 새로운 약물은 투여하지 않는 것을 원칙으로 한다.
2. 다만, 필요한 경우에는 시험담당자의 판단에 따라 투약할 수 있다. 연구담당자가 판단하기에 본 임상시험의 평가항목에 영향을 줄 수 있다고 예상되는 약물을 투약한 경우, 이 연구대상자는 탈락하게 된다 (예 : 항혈전제제(항혈소판제제, 항응고제제 포함), 향정신성 약물, 항우울약물, 갑상선기능항진증 관련 약물, 건강기능식품(홍삼), OTC 중 혈류개선제, 수부 족부냉증에 영향을 줄 가능성이 있는 약물 등).
3. 시험기간 중 다음 아래 약물을 추가 복용하지 말하야 함
예) 항혈전제제(항혈소판제제, 항응고제제 포함), 향정신성 약물, 항우울약물, 갑상선기능항진증 관련 약물, 건강기능식품(홍삼), OTC 중 혈류개선제, 수부 족부냉증에 영향을 줄 가능성이 있는 약물 등</td></tr>
<tr><td>시험방법</td><td>

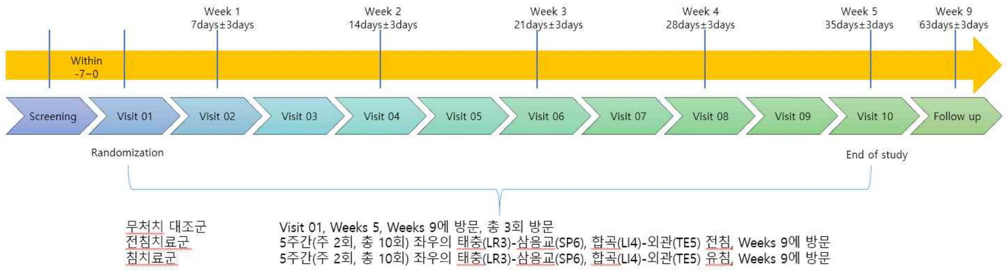

본 연구는 무작위배정, 다기관, 평행설계, 임상시험으로 진행한다. 연구대상자 선정기준에 따라 본 시험에 참여한 모든 연구대상자는 충분한 설명을 듣고 동의서에 서명한 후 연구대상자 식별코드를 부여받고 세 개 군인, 무처치대조군, 침치료군, 전침치료군으로 무작위 배정 후 실시한다. 각 연구대상자는 Screening 방문, 무작위배정 후, 주 2회 간격으로 총 10회 각 시험실시 기관을 방문한다. 스크리닝 포함 총 12회 방문한다. 시험기간은 5주이며, 종료 4주 후 추적 조사를 실시한다.</td></tr>
<tr><td>치료순응도</td><td>총 10회 방문 중 침치료 순응도가 70% 미만인 경우 탈락함.</td></tr>
<tr><td>유효성
평가방법</td><td>1. Primary outcome variable :
수부냉증의 시각적 상사척도 (Visual Analogue Scale ; VAS)
족부냉증의 시각적 상사척도 (Visual Analogue Scale ; VAS)
2. Secodary outcome variable :
(1) Body temperature : 써모미터 (Thermometer) 측정
(2) 변증설문지, 냉증 진단 설문지
(3) 삶의 질 설문지(WHOQOL-BREF)</td></tr>
<tr><td>안전성
평가방법</td><td>Vital Sign(매 방문시), 시험 종료시 혈액학적 검사(WBC, RBC, Hg, platelet), 혈액생화학적 검사 (BUN, Creatinine, AST, ALT, r-GTP)
시험담당자의 검진결과 및 이상반응 모니터링의 결과, 임상병리검사 결과를 통해 안전성을 평가한다.</td></tr>
</table>

통계분석	1. 유효성 평가 변수에 대한 분석<br>1) 기저선의 인구학적 및 평가 변수 비교<br>시험군과 대조군의 변수 분포가 동질한지 검정하기 위하여 연속형 변수에 대해 분산분석(ANOVA) 또는 비모수 방법을 이용하며, 범주형 변수에 대하여 Chi-square test 등을 실시한다.<br>2) 1차 유효성 평가 변수 수부·족부 냉증의 시각적 상사척도 (Visual Analogue Scale; VAS)<br>일차 유효성 평가 변수 분석은 ITT군을 주분석군으로 하며, 추가적으로 PP군 분석결과를 제시한다. 중재 이후 무처치대조군, 침치료군, 전침치료군간 VAS 점수 변화에 차이가 있는 지를 검정하기 위하여 ANOVA, repeated ANOVA, paired t-test 등을 실시한다. 각 군 내의 VAS 점수의 개선 정도의 비교는 중재 전후의 VAS 점수의 차이 (Weeks 5 ± 3days VAS 점수 – 방문 1의 VAS 점수)를 이용하여 paired t-test를 실시한다.<br>3) 2차 유효성 평가 변수<br>(1) Body Temperature(BT; 써모미터 측정) : 침치료군과 전침치료군간 BT 변화에 차이가 있는 지를 검정하기 위하여 repeated ANOVA 등을 실시한다. 각 군 내의 BT의 개선 정도의 비교는 침치료 전후의 BT의 차이 (Weeks 5±3days시의 BT – Visit 1 BT) 를 이용하여 paired t-test를 실시한다.<br>(2) WHOQOL-BREF 삶의 질 평가 설문지 : 각 군의 차이를 검정하기 위해 ANOVA 등과 사후검정을 이용하여 분석한다. 각 군 내의 비교는 전침치료 전후의 값 차이 (Weeks 5±3days 시의 값 – Visit 1의 값)를 이용하여 paired t-test를 실시한다.<br>(3) 변증설문지:<br>○ 변증을 위한 관찰대상(변증지표)가 수부·족부냉증을 가진 대상자들에서 어떤 분포를 보이는지 도시한다. 변증지표와 증상 VAS의 변화사이의 관계를 파악하기 위하여 각 지표마다 증상VAS의 변화량을 종속변수로 하여 ANOVA test(정규분포를 따르지 않는 경우 비모수 검사) 및 사후검정을 시행한다.<br>○ 기본변증도구의 타당도와 신뢰도 평가를 실시함. 타당도 평가는 a. 구인타당도평가(construct validity) : 기본변증도구 문항들의 요인분석을 시행하여 구인타당도를 평가함. 기본변증도구의 각 문항점수를 얻은 후, 문항들 간의 상관계수 행렬을 구함. 요인 고유값(eigen value)에 의하여 요인 수를 결정하고, 구인들을 확인한 후 요인을 회전시키고, 회전된 요인과 관계있는 요인 부하량이 큰 문항들의 문항내용에 근거하여 해석하고 이름을 부여함. b. 예측타당도(predictive validity) : 수족냉증 시각상사척도 값과 수족냉증을 설명하는 요인의 점수의 상관관계를 평가함. 수족냉증 환자의 시각상사척도 값과 구인타당도 평가결과 수족냉증을 잘 설명할 수 있는 문항의 점수 합과의 상관 정도를 추정함. 설문지의 평가를 위해 크롬바흐 알파를 측정한다. 이 외에 추가적으로, 치료 전후 해당 변증 항목의 변화와 각 항목의 정도의 변화, 특정한 변증 유형별 치료 효과의 크기 등을 분석 검토한다. 또한, 수족냉증에 관련된 변증항목과의 연관성과 변증 관련도를 파악하기 위해 군집분석 또는 요인분석을 활용하여 검토한다.

통계분석	(4) 냉증 진단 설문지:<br>○ 신뢰도 분석을 위해 크롬바흐 알파 분석을 실시한다. 또한, Item-Total Correlation의 통계 방법을 이용하여, 한 항목(item)이 잔존하는 나머지 항목의 전체 점수간의 피어슨 상관계수를 측정하여, 항목의 상관성을 분석한다.<br>○ 군집분석(cluster analysis)과 요인분석(factor analysis)를 추가적으로 실시한다.<br><br>2. 안전성 평가 변수에 대한 분석<br>1) 임상실험실 검사 자료에 대해서는 변수의 특성에 따라 치료 전, 후의 군내 비교 등 적절한 통계적 방법을 이용하여 분석하고, 이상반응의 빈도, 발현율, 각각의 목록, 상세한 발현시간, 심각한 정도 및 침 치료와의 인과관계 등을 제시하며, 필요한 경우 그래프 형태로 보고한다. 이에 대한 통계적 분석은 변수의 특성에 따라 ANOVA, t-test, chi-square 혹은 Fisher's exact test 등을 실시한다.<br>2) 자·타각 증상 등 이상반응: 발현된 모든 이상반응은 각 치료군별 및 신체기관별로 분류하여 자세한 설명과 함께 table 화 한다. 각 군 별로 침치료와의 인과관계가 있는 이상반응과 인과관계가 없는 이상반응의 빈도를 기록하다. 이상반응 발현건수와 1 회 이상의 이상반응을 경험한 연구대상자의 비율에 대하여 군내에서 95% 신뢰구간을 구하고 군간 비교를 실시한다.<br>3) 임상병리검사 : 혈액학 및 혈액생화학 검사 결과, 활력징후(vital signs) 등과 같은 연속형 자료에 대해서는 각 군별 및 방문별로 기술통계량을 제시하며 방문 시점간의 차이에 대하여 paired t-test 또는 비모수 방법을 이용하여 분석한다.<br><br>3. 결측치 분석<br>1) 통계분석에서 유효성 평가 변수 중, 계획된 시험에서 얻을 수 있는 관측값 중에 어떠한 이유로 관측되지 않은 측정값, 즉 결측값(missing value)인 경우에는 이전 방분의 측정값으로 대체하는 LOCF(Last Observation Carried Forwar)방법을 사용하여 분석한다. 안전성 평가 변수의 경우 결측치는 결측으로 처리하여 분석한다.

목 차

<임상연구 일정 요약> 1
1. 임상시험의 명칭 및 단계 2
2. 임상시험실시기관명 및 주소 2
3. 인체적용 시험 실시기관별 임상 시험 관련 담당자 성명 및 수탁기관의 업무요약 2
4. 임상시험의뢰자명 및 주소 3
5. 배경 및 목적 3
6. 침 치료에 대한 정보 6
7. 연구대상자 선정기준/제외기준 및 목표한 연구대상자의 수 및 그 근거 9
8. 임상시험기간 11
9. 시험방법 11
10. 관찰 및 검사 항목 18
11. 침치료의 예측 이상반응 및 사용상의 주의사항 28
12. 임상시험 중지, 연구대상자 탈락기준 29
13. 안전성의 평가기준, 평가방법 및 보고방법 29
14. 유효성 평가방법 및 해석방법 34
15. 통계분석방법 36
16. 임상시험의 순응도와 프로토콜 위반에 대한 처리 38
17. 자료관리 38
18. 임상시험참가자 설명문 및 동의서 39
19. 피해자 보상에 대한 규약 39
20. 임상시험 후 연구대상자의 진료 및 치료기준(방안) 39
21. 연구대상자의 안전 보호에 관한 대책 및 기타 39
22. 기타 임상시험을 안전하고 과학적으로 실시하기 위하여 필요한 사항 40
23. 참고문헌 42
[별첨1] 피해자 보상에 대한 규약 43
[별첨] 헬싱키 선언 44

<임상연구 일정 요약>

	스크리닝	Treatment period			
방문	(D -7~0)	방문 1<br>(D 1)	방문 2~9	방문 10<br>(D 35±3)	방문11<br>(D 63±3)
서면동의서 취득	●				
스크리닝 번호 부여	●				
Chest X-ray & EKG1)	●				
선정/제외기준2)	●				
무작위 배정		●			
무처치대조군		■		■	■
치료순응도 평가			○	○	○
연구대상자의 중도탈락 여부			○	○	○
활력징후	●	●	○	●	●
신장, 체중, 신체질량지수(BMI)	●			●(체중만)	●(체중만)
연구 참여 경로 및 동기	●				
인구학적 사회학적 조사, 산부인과적 정보력3)	●				
병력 및 약물 투여력 조사4) 및 변화 확인	●	●	○	●	●
체질진단 설문(KS-15)	●				
검사					
일반신체검사	●	●	○	●	●
써모미터 측정5)	●	●	○	●	●
수부냉증 및 족부냉증 VAS	●	●	○6)	●	●
이상반응 검사 및 유무 확인			○	●	●
변증 설문지	●				
냉증진단 설문지		●		●	●
삶의 질 평가 설문지(WHOQOL-BREF)		●		●	●
삶의 질 평가 EQ-5D		●		●	●
실험실적 검사	●7)			○	
경혈침 또는 전침치료		○	○	○	
다음 방문일 지정		●	○	●	
시험자 서명	●	●	●	●	●

● 세 군 모두 실시
○ 침치료군, 전침치료군만 실시
■ 무처치대조군 방문일

1) Screening 시에는 흉부 X-ray, 심전도 실시함
2) Screening 시에 실시함
3) 연령, 직업, 소화력, 운동여부, 흡연, 음주, 수면, 시험참여 동기 등을 조사함.
4) 수족냉증 관련 병력 및 약물력 포함
5) 방문시마다 양쪽 전대퇴 중심부(ST32)과 족배부 전면부(LR3) 및 수장부위(PC8)와 전완부 중심부(LU4) 온도 측정함
6) 침치료군과 전치치료군은 방문 4, 방문 8에 수부냉증 및 족부냉증 VAS를 추가로 측정함.
7) Screening 시에는 혈액학적검사(WBC, RBC, Hemoglobin, platelet), 혈액생화학적검사(BUN, creatinine, AST, ALT, r-GTP, glucose), 갑상선기능검사(free T4, TSH), 소변 및 요침사검사, 임신검사(Urine HCG)를 실시함. D35(방문10)방문 시에는 혈액학적 검사(WBC, RBC, Hg, platelet), 혈액생화학적검사 (BUN, Creatinine, AST, ALT, r-GTP) 시행함.

1. 임상시험의 명칭 및 단계

가. 명칭: **수족냉증에 대한 전침 치료의 유효성 안전성에 대한 다기관 임상연구**

- 무작위배정, 대조군 임상시험 -

나. 단계 : 연구자임상시험용

2. 임상시험실시기관명 및 주소

가. 세명대학교 충주한방병원
주소 : 충청북도 충주시 상방4길 63(봉방동)
전화 043-841-1733, 팩스 043-856-1731

나. 상지대학교 부속한방병원
주소 : 강원도 원주시 상지대길 80
전화 033-732-2111, 팩스 033-732-2124

다. 동국대학교 일산한방병원
주소 : 경기도 고양시 일산동구 동국로 27
전화 : 031-961-9126, 031-961-9009

3. 인체적용 시험 실시기관별 임상 시험 관련 담당자 성명 및 수탁기관 업무요약

가. 임상시험 실시기관별 시험 책임자, 공동연구자, 임상시험 담당자 및 의료기기 관리자

각 실시기관별 연구책임, 공동연구자, 코디네이터, 시험담당자, 임상시험용 의료기기 관리자 및 모니터 요원 등은 각 실시기관별 심의 신청서 또는 별지로 제출함.

나. 인체적용시험 수탁기관 및 업무 요약

경희대학교 한방병원 한의약 임상시험 센터(서울특별시 동대문구 경희대로 23)

역할: 대상자의 안전 및 권리의 보호 ,데이터의 완결성, 신뢰성 및 질 보장, 임상시험이 수행되는 임상시험 실시기관(Site) 간 데이터의 일관성 보장, 임상시험을 수행하는 모니터 요원(Monitor)에게 모니터링의 최소 요구사항에 대한 가이드라인 제시, 임상시험이 국제조화회의 임상시험관리기준(ICH-GCP), 헬싱키 선언, 관계 당국 규정, 의뢰자 와 경희대학교한방병원 한의약임상시험센터(K-CTC)가 따르기로 합의한 SOP 에 따라 적절하게 수행되는지의 확인

4. 임상시험의뢰자명 및 주소

가. 임상시험의뢰자 : 상지대학교 산학협력단

나. 주소 : 강원도 원주시 상지대길 80 상지대학교 산학협력단

5. 배경 및 목적

가. 배경

□ 냉증의 개념

○ 냉증이라고 하는 것은 '신체의 다른 부분은 전혀 냉감을 느끼지 않는 실온에도 불구하고 신체의 특정 부위만이 차가움을 느끼는 경우'로 정의하고 있으며 의학적으로 '냉각과민증'이라고 한다. (이수림, 이경섭, 송병기. 부인냉증에 관한 문헌적 고찰. 대한한방부인과학회지 9(1):55-80, 1996)

○ 냉증은 일반적으로 추위를 느끼지 않을 만한 온도에서 신체의 특정부위가 차고 시려서 일상생활을 유지하기 곤란한 상태를 일컫는다(현대의 한방치료 한성출판사 1989).

○ 냉증은 특히 여성에게서 많이 나타나는데 빈도는 전신, 수족, 소복, 음부, 요부, 배부의 순이며 대하, 산후풍, 자연유산 등과 관련이 깊다(부인과 환자의 냉증과의 관계에 대한 조사 연구 대한한방부인과학회지 15(2); 2002)

○ 냉증이란 개념은 ICD-10에 없는 증상명으로, 환자 개인에게는 손발 시림, 남과 악수하기 꺼려짐, 여름에도 두꺼운 양말을 신어야 하는 등의 삶의 질을 떨어뜨리는 질환으로서 이에 대해 효과적인 치료가 필요하지만, 이에 대애 양의학적 뚜렷한 치료방법은 없이 단순하게 혈액순환개선제 등을 사용하나 그 효과에 대해 미지수이며, 한의학에서는 침, 뜸, 한약 등을 이용하여 치료하고 있기에 한의학이 우선 치료의 목적이 될 수 있음.(조기호. 동서의학 진료 가이드 북. 서울: 고려의학; 2001, p. 284-6.)

□ 기존의 냉증에 관한 연구들(냉증 진단 방법 등)

○ 적외선 피부온도 촬영기를 이용한 수족냉증 진단의 표준화

: 수족냉증을 호소하는 환자 50명과 수족냉증을 호소하지 않는 환자 50명의 두 그룹으로 나누어 각각의 적외선 체열촬영상 수족의 온도차를 구한 결과 수냉증의 경우 수장부위(PC8)와 전상완 중심부(LU4)의 적외선 체열촬영상 온도차가 0.3℃이상일 때 민감도는 94.0%, 특이도는 90.0%로 높게 측정되어 냉증 진단의 타당성이 인정되었다. **족냉증의 경우 족배부 전면(LR3)과 전대퇴중심부**

(ST32)의 적외선 체열 촬영상 온도차가 2.0℃ 이상일 때 민감도는 94%, 특이도는 76%로 높게 측정되었다. (김동환 외. DITI를 이용한 수족 냉증 진단의 표준화. 대한한방부인과학회지. 2001;14(2): 120-134)

○ 냉부하 검사를 통한 수족냉증의 진단
: 손의 냉부하검사는 1976년부터 시도되어 왔으며, 주로 레이노이드 증후군, 당뇨환자의 말초순환장애, 흡연이나 진동노출에 따른 혈관장애, RDS증후군 등을 진단하는데 활용되어 왔다. 이들은 주로 체열촬영방법으로 이루어 졌지만 그 외 고빈도 초음파를 사용하거나 디지털혈량계를 사용하여 냉부하검사를 시행하기도 하였다. (한지영 외.냉부하검사를 통한 수부냉증의 진단. 2003;2(1):17-23)

○ 냉증진단을 위한 적외선 피부온도 촬영기와 말초신경검사의 임상적 평가
: 경한 냉증군과 심한 냉증군 사이에 손에서의 정중신경, 척골신경, 요골신경에서 측정한 전기적 감각인지 실험에서 냉증이 심한군에서 더 둔한 전기감각이 나왔으며, 경한 냉증군과 심한 냉증과 사이에 발에서 측정한 비골신경, 비복신경, 경골신경, 복재신경 부위에서의 각 진동수에 따른 평균 전기감각인지수치도 심한 냉증군에서 더 둔하게 나왔다.(이경섭. 냉증 진단을 위한 DITI와 말초신경검사의 임상적 평가.한방체열의학회지. 2004;3(1):60-66)

○ 냉증을 호소하는 환자의 Heart Rate Variability 특성 연구
: 한의학적으로 정상체온을 유지하는 것은 기의 체온조절작용이 있기 때문인데 만약 칠정, 외감, 노상 등의 원인으로 기의 온후(溫厚)작용이 실조되면 체온조절작용을 상실하게 되어 냉증이 발생하게 된다. 그러므로 냉증환자군의 특성을 HRV를 통해 확인하고 HRV검사를 통해 냉증 환자의 진단 및 예후 판단에 도움이 될 수 있음을 알 수 있었다.(이미주 외. 강남경희한방병원에 내원한 냉증을 호소하는 여성의 Heart Rate Variability 특성 연구. 대한한방부인과학회지.2011;24(3):109-115)

○ 적외선 체열촬영을 이용한 배변습관과 하복 및 수족냉증의 관련성 고찰
: 수족과 복부의 온도차와 비교하여 수족냉증 및 복부냉증이 배변습관과 상관성을 가지는지를 조사한 결과 수냉증이 변비군, 일반군, 설사군의 순으로 심해지는 것을 알 수 있었으며, 족냉증의 경우도 변비군, 일반군, 설사군의 순으로 심해져서 수족냉증과 설사가 관련성을 가진다는 임상적 근거를 확보할 수 있었으며 수족냉증과 기능성장질환과의 관계성 연구에 도움이 되었다.(윤성우 외. 적외선 체열촬영을 이용한 배변습관과 하복 및 수족 냉증의 관련성 고찰. 한방체열의학회지. 2004;3(1):36-42)

○ 산후풍 환자의 적외선 체열 영상 특성에 대한 연구
: 신체부위의 시린감과 추위를 잘 느끼게 되는 산후풍 환자에 대하여 실제로 신체의 체온이 차가운지를 적외선 체열진단기로 측정하여 일반인과의 차이를 객관적으로 살펴보았다.(박경선 외. 산후풍 환자의 적외선 체열 영상 특성에 대한 연구. 대한한방부인과학회지. 2010;23(2):116-123)

○ 적외선 촬영기를 통한 불임여성의 체열에 대한 연구
: 불임 환자군에서 적외선촬영기로 체표면의 온도를 측정한 결과 다른 부위보다도 하복부에서 불임 환자군이 일반인에 비하여 온도가 낮은 것으로 측정되었으며 무릎과 허리 쪽의 온도도 일반

인에 비하여 온도가 유의하게 낮은 것으로 나타났다.(김혜원 외. DITI를 통한 불임여성의 체열에 대한 연구. 대한한방체열의학회지. 2002;1(1):52-56)

○ 냉증에 혈액순환개선제 투여 : 수족냉증을 접한 의사의 60%가 혈액순환개선제를 처방하고 있음.(김철민 외. 수족냉증과 레이노병에 대한 일부 가정의의 인식도. 대한가정의학회지. 2007;28:339-45.)

○ 수족냉증과 혈액순환장애의 상관성을 연구한 결과, 미세혈류 순환장애의 진단으로 적혈구 변형능을 조사한 결과 수족냉증 환자에서 적혈구 변형능이 낮게 나타남을 확인.(이에 따라 적혈구의 산소 전달 능력 및 미세 혈관으로의 산소 전달능이 떨어질 것으로 사료됨.)(박주영 외. 중풍환자에서 수족냉증과 적혈구변형능의 관련성 연구. 대한한방내과학회지. 2010;31(3):578-85),

○ 말초신경염에 대한 온침구치료 : 말초신경염 환자를 대상으로 온침구를 치료시 치료군에서는 治愈26例, 好转6例, 未愈2例이며, 对照组에서는 治愈5例, 好转7例, 未愈19例로 나왔다. 。.(张欣, 李永凯. 温针灸治疗末梢神经炎之感觉障碍临床观察. 新疆中医药2007 年第25 卷第6 期(总第112 期)

○ 레이노병은 차가운 자극에 의해 손발의 혈관이 수축되어 혈액순환장애를 일으키는 질환으로, 이러한 레이노병에 침치료가 효과적이며, 이는 침치료가 말초혈관 혈류개선 효과에 기인한다고 하였으며, 치료시 사용하는 혈위로 상지에는 합곡(LI4), 내관(PC6)외관(TE5), 양지(TE4), 팔사(EX-UE9), 극천(HT1), 비중(EX-23), 수오리(LI13), 척택(LU5) 등이, 하지에는 태충(LR3), 삼음교(SP6), 족삼리(ST36), 팔풍(EX-LE10), 해계(ST41), 음릉천(SP9) 등이 사용되었음. (전상우 외. 레이노병의 침치료에 대한 연구동향 대한한방내과학회지 2017;38(4):433-442)

나. 목적

1) 일반적인 목적

본 임상시험은 수족냉증 환자에게 경혈치료 또는 전침 치료 후 수족냉증 완화에 대한 유효성과 안전성을 다기관, 무작위배정, 대조군 임상시험을 통해 객관적으로 비교 평가하기 위함이다.

2) 일차 목적

경혈(합곡-태충, 태충-삼음교)에 15분간 유침, 주 2회, 총 10회 침 치료 또는 같은 경혈에 전침을 추가한 이후, 수족냉감(수족부냉증)의 시각적 상사척도(Visual Analogue Scale : VAS) 점수의 변화를 비교하여, 무처치대조군, 침치료군 및 전침치료군간의 유효성을 비교 평가한다.

3) 이차 목적

수족부냉감의 시각적 상사척도, 체온 (써모미터 측정), 삶의 질 평가 설문지(WHOQOL-BREF), 삶의 질 평가 EQ-5D, 변증 설문지, 냉증 진단 설문지 등의 점수변화를 비교하여 무처치대조군, 침치료군 및 전침치료군간의 유효성을 부가적으로 비교 평가한다.

4) 안전성 평가

이상반응, 임상실험실 검사, 이학적 검사를 통해 안전성을 평가한다.

6. 침 치료에 대한 정보

가. 혈위

1. 침치료군 및 전침치료군

(1) 합곡혈(LI4)

위치 : 손등, 둘째 손허리뼈(the 2nd metacarpal bone) 중점의 노쪽. 直刺 0.5 ~ 1寸

효능 : 發表解熱, 疏散風邪, 淸泄肺氣, 通降腸胃, 鎭痛安神

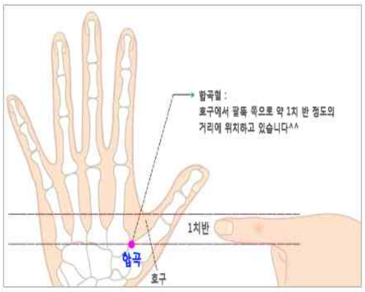

(2) 외관혈(TE5)

위치 : 陽池(TE4)에서 몸 쪽으로 2寸, 자뼈와 노뼈 사이의 오목한 곳에서 취혈한다. 外關(TE5)에 상응하는 앞쪽 經穴은 內關(PC6)이다. 直刺 0.5 ~ 1寸 (혹은 內關(PC6)을 향해 透刺하기도 한다)

효능 : 淸熱解毒, 通經活絡, 祛六淫表邪, 疎三焦壅熱

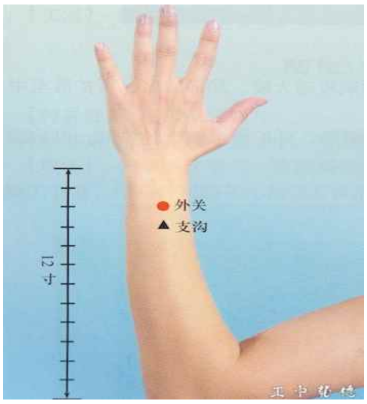

(3) 태충혈(LR3)

위치 : 손가락을 行間(LR2)에서부터 첫째와 둘째 발허리뼈 사이로 그 바닥을 향하여 몸 쪽으로 움직일 때 촉지되는 오목한 곳에서 취혈한다. 直刺 0.3 ~ 0.5寸

효능 : 淸熄肝火肝陽, 疎泄下焦濕熱, 舒肝理氣, 通絡活血

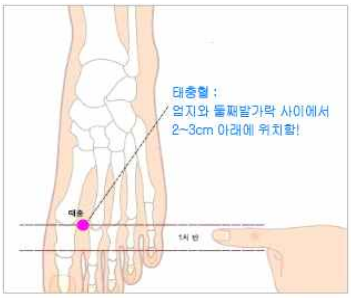

(4) 삼음교혈(SP6)

위치 : 종아리 정강뼈면, 정강뼈 안쪽모서리 뒤쪽에 해당되며, 안쪽복사 융기에서 음릉천까지 13촌으로 정했을 때 안쪽복사융기 위 3寸 부위에 취혈함. 바로 1촌 아래지점에 족소음신경의 交信(KI8)이 위치한다. 1~1.5寸 直刺

효능 : 扶脾土, 助運化, 通氣滯, 疎下焦, 調血室精宮, 祛經絡風濕

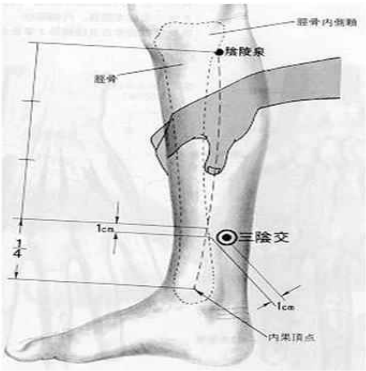

나. 임상시험에 사용되는 침, 전침기 및 처치 방법

1. 침종류

0.20 × 30 mm 의 멸균침 (대한민국, 동방메디컬)

2. 포장, 라벨 및 보관방법

1회용, 밀봉, 임상시험센터내 실온 보관

3. 임상시험용 전침기

전침기는 STN-330(스트라텍, 대한민국)을 이용한다. 상세자료 별첨.

4. **전침기 사용방법**

- 준비

1) 본체 뒷면의 전원 코드를 전원에 연결하고, 메인 스위치를 켠다.

2) 모든 출력다이얼이 「0」으로 설정되고, 기기의 설정모드가 정상작동중임을 확인한다.

- 침전기자극을 행할 경우

1) 전침기 연결에 사용되는 전용 연결선이 외피가 벗겨지는 외관상 손상이 없음을 확인하고, 각각의 연결선에 적색과 검은색의 전용클립이 연결되어 있음을 확인한다.

2) 전용 연결선을 전침기의 출력구(전극코드)에 확실히 접속한다.

3) 전용 연결선의 적색 및 검은색의 전용클립으로, 멸균침에 접속한다.

4) 전원스위치를 누른 후, 원하는 Hz, 시간, 또는 program을 설정한다.

5) 밸브 스위치를 천천히 돌리며 환자에게 적절한 수준의 강도로 조절하여 시술한다.

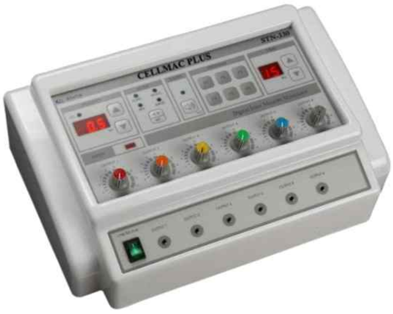

Specifications	
Items	Specification
정격 입력 전압	220V/60Hz
정격 소비 전력	20VA
최대 출력 전류	HIGH(13.0mA) / LOW(7.3mA)
최대 출력 전압	HIGH(90Vp-p)<br>LOW(60Vp-p) – 500Ω LOAD시
출력 파형	쌍극성 대칭파
펄스 폭	55㎲
자극 주파수	1~240㎐
기능 모드	4종 [CONT. / MODU. / MIX / MASS.]
내장 프로그램	6종
최대 자극 시간	1~99분[1분 단위]
채널 수	6채널
전기 충격 보호 형식	1급 기기, BF형 장착부
크기	30 x 220 x 126 mm
중량	약 2.04Kg

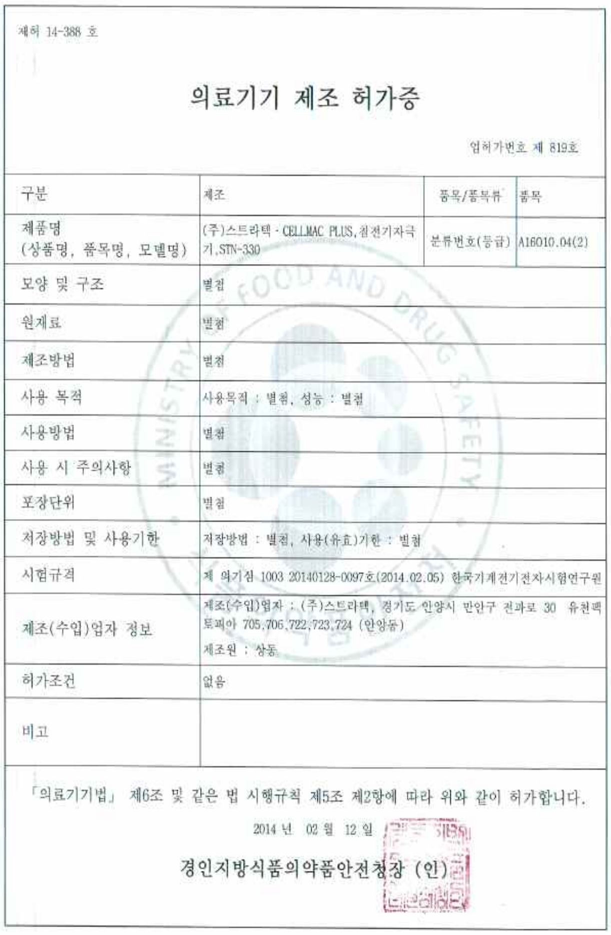
제허 14-388 호

의료기기 제조 허가증

업허가번호 제 819호

구분	제조	품목/품목류	품목
제품명<br>(상품명, 품목명, 모델명)	(주)스트라텍 - CELLMAC PLUS,침전기자극기,STN-330	분류번호(등급)	A16010.04(2)
모양 및 구조	별첨		
원재료	별첨		
제조방법	별첨		
사용 목적	사용목적 : 별첨, 성능 : 별첨		
사용방법	별첨		
사용 시 주의사항	별첨		
포장단위	별첨		
저장방법 및 사용기한	저장방법 : 별첨, 사용(유효)기한 : 별첨		
시험규격	제 의기심 1003 20140128-0097호(2014.02.05) 한국기계전기전자시험연구원		
제조(수입)업자 정보	제조(수입)업자 : (주)스트라텍, 경기도 안양시 만안구 전파로 30 유천팩토피아 705,706,722,723,724 (안양동)<br>제조원 : 상동		
허가조건	없음		
비고			

「의료기기법」 제6조 및 같은 법 시행규칙 제5조 제2항에 따라 위와 같이 허가합니다.

2014 년 02 월 12 일

경인지방식품의약품안전청장 (인)

그림 1. 침전기 자극기(STN 330) 제원 및 제조 허가증

5. **전침 자극방법**

1) 전침자극 :

- 침치료군은 좌우의 태충(LR3), 삼음교(SP6), 합곡(LI4), 외관(TE5) 총 8곳에, 피하 10~25 mm의 깊이로 자침하고, 15분간 유침후 발침한다. 전침기를 사용하지 않는다.
- 전침치료군은 좌우의 태충(LR3), 삼음교(SP6), 합곡(LI4), 외관(TE5) 총 8곳에, 피하 10~25 mm의 깊이로 자침하고, 태충(LR3)은 삼음교(SP6)에, 합곡(LI4)은 외관(TE5)에 전침기에 연결된 전용클립으로 각각 연결한다(적색, 검은 색 구분없음).
- 전침기의 전원스위치를 누르고, 화면 설정에서 주파수는 2Hz, 자극시간은 15분으로 설정하고, 개별 연결선의 스위치를 우측으로 천천히 돌리면서 환자에게 통증을 느끼지 않는 적절한 수준의 강도로 조절하여 시술한다.
- 치료빈도는 주 2회, 총 5주간 총 10회 침시술과 함께 전침치료를 병행한다.

다. 침 치료상의 주의방법

침 치료시 통증, 어혈, 저린 감 등을 유발 할 수 있으므로 주의 해야한다.

7. 연구대상자 선정기준/제외기준 및 목표한 연구대상자의 수 및 그 근거

가. 선정기준

: 다음의 모든 기준에 적합하여야 한다.

1. 수족냉감을 호소하는 만 19세 이상 만 59세 이하의 여성
2. 아래 정의에 해당하고, 범위항목 중 한 가지 이상 해당하는 자
   (1) 정의 : 손과 발이 차갑게 느껴져 불편함을 호소하는 자
   (2) 범위 항목
   첫째, 추위를 느끼지 않을 만한 온도에서 수부 족부냉증을 느끼는 경우
   둘째, 추위를 느낄 만한 온도에서 주변 사람보다 과도하게 수부 족부냉증을 느끼는 경우
   셋째, 추운 환경에서 따뜻한 환경으로 이동 시 증상이 쉽게 회복되지 않는 경우
3. 스크리닝 방문 당시, 수부 또는 족부냉증의 시각적 사상척도(Visual Analogue Scale : VAS)가 4 이상인 자.
4. 스크리닝 당시, 상온 노출 상태에서 10분간 24℃(±2)의 실온 적응 후 수장부위(PC8)와 전완부 중심부(LU4)의 온도차가 0.3℃ 이상이거나, 전대퇴부중심부(ST32)와 족배부전면(LR3)의 온도차가 2.0℃ 이상인 자
5. 임상시험 수행 중 추적관찰이 가능한 자
6. 본 임상 연구의 목적 및 특성에 대한 명백한 설명을 들은 후
   참여에 동의하고 동의서에 서명한 자.

나. 제외기준

: 다음 조건의 하나라도 해당되는 자는 본 연구에 참여할 수 없다.

1. 수족냉증 치료 목적으로 칼슘길항제 혹은 베타차단제를 복용하고 있는 자.
2. 손가락에 궤양 또는 괴저가 1개 이상 있는 자
3. 갑상선기능저하증을 진단받았거나, 갑상선 관련 약물을 복용하는 자
4. 자가면역질환을 진단받은자
5. 수근관 또는 족근관증후군을 진단 받았거나, 팔렌 티넬 검사에서 양성 결과 나온 자
6. 경추 또는 요추 추간판 탈출증을 진단받은 자
7. 당뇨병을 진단 받은 자
8. 수부 족부냉증에 영향을 줄 수 있는 약물 복용자 (예, 항응고제 등)
9. 중증의 간 질환자(AST·ALT 각 100 IU/L 이상,), 신부전 환자(Creatinine 2.0mg/dL 이상)
10. 행동장애 또는 우울증, 불안신경증 및 심각한 정신장애를 가진 자
11. 성인여자(비임신) Hemoglobin 7g/dL 미만, WBC 11,000/mm3 이상,
12. 2회 측정하여 평균 수축기 혈압 180mmHg 또는 이완기 혈압 100mmHg 이상
13. 심전도 상 치료가 필요한 부정맥이거나, 허혈성심질환 등 심장질환 자
14. 알콜이나 약물 남용자
15. 임신부(요중 hCG 양성), 수유부, 임신계획이 있거나 적절한 피임방법 선택에 동의하지 않는 가임여성
16. 악성종양을 진단받았거나 치료 경험자

17. 다른 임상연구에 참가 중이거나 연구 종료후 2개월 이내인 자.
18. 임상시험에 참여 혹은 동의서에 제공을 거부한 자
19. 한국어를 이해하거나 표현할 능력이 부족한 자
20. 시험자의 판단에 의거 임상시험에 부적합하다고 판단된 자

다. 연구대상자 수

1. 목표한 시험대상자수

	침치료군	전침치료군	무처치<br>대조군	총<br>대상자수
유효평가 대상자 수	18	18	18	54
탈락률 25%를 고려한 대상자 수	24	24	24	72

2. 산출근거

본 시험은 수족냉증 환자를 대상으로 침치료를 진행했을 경우, 침치료군과 전침치료군이 무처치 대조군에 비하여 증상이 개선될 것이라는 가정하에 설계되었으며, 상기 가정에 근거한 가설은 다음과 같다.

이 연구의 가설은 다음과 같다.

H0 : $\delta = \Delta 1 - \Delta 2 = 0$
H1 : $\delta = \Delta 1 - \Delta 2 \neq 0$

H0 : $\delta = \Delta 1 - \Delta 3 = 0$
H1 : $\delta = \Delta 1 - \Delta 3 \neq 0$

Δ1 : 침치료군에서 치료전후의 VAS 점수 평균변화량
침치료군에서 무작위 배정후 10주차(±3일 이내)에 수족냉증의 VAS 점수의 평균 변화량
Δ2 : 전침치료군에서 치료전후의 VAS 점수 평균변화량
전침치료군에서 무작위 배정 후 10주차(±3일 이내)에 수족냉증의 VAS 점수의 평균 변화량
Δ3 : 무처치 대조군에서 치료전후의 VAS 점수 평균변화량
무처치 대조군에서 무작위 배정 후 10주차(±3일 이내)에 수족냉증의 VAS 점수의 평균 변화량

의료기기인 침과 전침으로 실시한 수족냉증 임상시험은 산소챔버기기의 임상시험의 VAS 정보를 근거로 시험대상자수를 산출하고자 한다.

참조한 하 헌용 등의 임상시험의 결과[20]에서, placebo 처치군 VAS score의 VAS difference 평균은 -0.48 이며, 표준편차 1.030이며, 산소챔버처지군 VAS score의 VAS

difference 평균은 -1.520이며, 표준편차 1.17 이다. 이것으로 예상되는 수족냉증 침치료 difference로 예상되는 차이는 1.04며, 합동표준편차 공식을 이용하여 표준편차는 1.102로 추정되었다.

$$s_p = \sqrt{\frac{(n_c-1)s_c^2+(n_t-1)s_t^2}{n_c+n_t-2}}$$

그림 9 합동표준편차 공식

제 1종 오류를 범할 확률인 유의수준(α)은 0.05을 적용하였으며, 제 2종 오류를 범할 확률(β)는 0.2로 가정하였다. 시험군(전침치료군)과 무처치대조군의 대상자수를 동일하게 설정하고, 두 집단의 평균비교를 통한 시험대상자 추정 공식은 다음과 같다.

$$n = \frac{2(z_{\alpha/2}+z_\beta)^2\sigma^2}{(\mu_c-\mu_t)^2}$$

n=[2*(1.96+0.84)^2*1.17^2]/(1.04)^2=17.625 ≒ 18

상기내용을 바탕으로 시험대상자 수를 산출하면 각 군당 18명이며, 탈락율 25%을 고려하여, 군당 24명이 등록되어야 한다.

18÷(1-0.25)=24

따라서 본 임상시험은 침치료군, 전침치료군, 무처치대조군 각각 24명씩 총 72명을 등록하여, 분석하도록 계획한다.

8. 임상시험기간

기관생명윤리위원회(Institute of Review Board, IRB) 승인일 이후 24개월. 단, 시험 대상자 등록 속도에 따라 연장될 수 있다.

9. 시험방법

가. 시험 설계

본 연구는 무작위배정, 다기관, 평행설계, 임상시험으로 진행한다. 본 연구 관련자로부터 충분한 설명을 듣고, 본인의 자발적 의지로 동의서에 서명한 대상자는 임상연구 계획서에 따라 선정기준 및 제외기준을 검토하여 임상시험에 적합하다고 판단되면, 연구대상자 식별코드를 부여받고, 혈액검사를 실시하고, 추후 선별 방문일에 방문하여, 침치료군, 전침치료군, 무처치대조군 등으로 무작위 배정하여 임상시험을 실시한다

각 연구대상자는 Screening 방문, 무작위 배정 후, 침치료군, 전침치료군으로 배정된 경우에는 주 2회 간격으로 총 5주 10회 방문하여 침치료 또는 전침치료를 각각 실시한다. 이후 4주후 추적

조사를 실시한다. 무처치대조군인 경우에는 Vist 1. 5주후, 9주후 총 3회 방문한다.

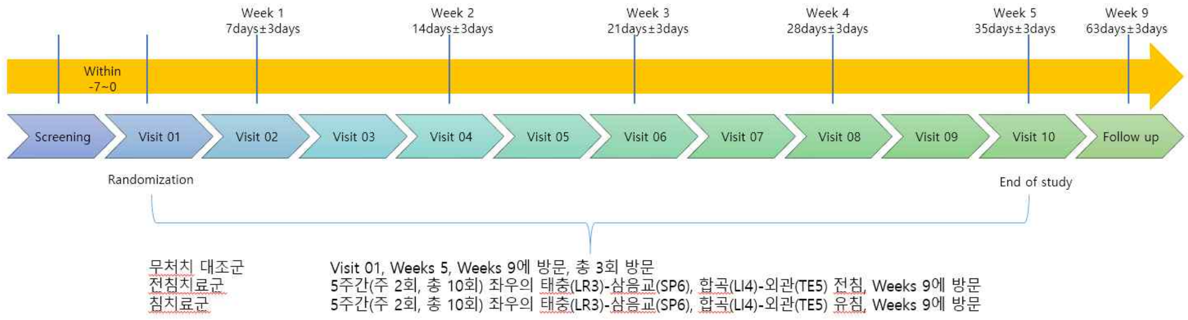

나. 무작위배정방법과 눈가림

1) 개요

자의에 의해 임상시험 동의서에 서명한 대상자는 임상연구 계획서에 따라 필요한 검진 및 검사를 실시한 후, 선정기준 및 제외기준을 검토하여 본 임상시험에 적합하다고 판단되면, 방문 1에 침치료군, 전침치료군, 무처치대조군에 무작위 배정한다. 대상자는 시술방법 및 방문일 등을 교육받고 임상연구 일정에 따라 5주(±3일) 주 2회, 총 10회의 시술을 받거나 일상생활을 하게 된다. 세 군 모두 무작위 배정 5주(±3일)후에 유효성을 측정하고, 9주(±3일)후에 추적관찰을 한다.

2) 무작위배정법 및 층화방법

본 임상시험에 참여하기로 한 시험대상자는 서면 동의 순서에 따라 스크리닝 번호를 부여 받으며, 만일 하루에 2인 이상 스크리닝을 실시하는 경우에는 동의한 순서대로 스크리닝 번호를 부여한다. 무작위 배정 코드는 침치료군, 전침치료군, 무처치 대조군의의 비를 1:1:1 로 하여 독립된 통계학자 또는 제3의 독립된 연구자에 의해 제공되며, 무작위 배정 번호의 생성은 R 3.4.2 또는 이후 버전의 blockrand package를 이용하되, 무작위 배정시 기관에 따라 층화하며, 군별 대상자 숫자가 거의 동일하도록 블록 무작위배정을 사용한다. 각 시험기관에서는 스크리닝 완료 순서에 따라 대상자 번호를 부여한다.

3) 배정은폐

본 임상시험에서는 sealed envelope method를 사용한다. 무작위 배정 코드를 생성한 독립된 통계학자 또는 제3의 독립된 연구자는 무작위 배정 코드가 적힌 불투명한 봉투 안에 배정군이 기재된 종이를 넣고 밀봉 후 각 기관별 허가된 담당자에게 제공한다. 서면 동의를 실시한 대상자가 스크리닝 평가를 통과할 경우, 담당자는 스크리닝 평가 완료 순서에 따라 봉투를 열어 해당 대상자의 무작위 배정 코드 및 할당군을 확인한 후 기록한다.

4) 눈가림

본 임상시험은 모든 군 배정결과에 대한 눈가림이 불가능한 공개(open-label) 시험에 해당한다. 단, 대상자의 증상을 평가하는 평가자를 시술자와 분리하여 눈가림함으로써, 평가시 발생할 수 있는 비뚫림을 최소화하고자 한다.

다. 시험일정

1) 연구대상자모집

각 시험실시 기관의 방문환자 혹은 옥외 광고를 통한 공개모집 등의 경로로 자원자를 모집한다.

2) 시험동의 및 연구대상자식별코드 부여

임상시험에 대해 충분히 설명한 후 서면으로 작성된 동의서를 받으며 이 순서에 따라 연구대상자 식별 코드를 부여한다.

임상연구 실시기관의 직원이나 소속 대학의 학생이 참여를 원할 경우, 본 연구와 무관한 대상에 한해 시행 될 것이며, 자발적인 참여 동기가 있는 직원 또는 학생을 대상으로 연구에 대해 충분한 설명을 시행하며, 이에 동의한 경우에만 자필 동의서 작성을 안내하고 서명함. (예시: "병원의 직원 또는 대학의 학생이며, 연구에 대한 충분한 설명을 들었고 자발적 의지로 참여하여 동의서에 서명함")

3) 대상자 선정

(1) 인구학적, 사회학적 정보 / 기타 치료 전 특성

Screening 번호를 부여한 후 인구학적, 사회학적 정보를 조사한다. 기록사항은 아래와 같다.

- 서면 동의 여부 및 동의 일자
- 대상자 이니셜
- 연령
- 혈압, 신장, 체중
- 직업
- 소화력
- 운동 여부 및 관련 정보
- 흡연 여부 및 관련 정보
- 음주 여부 및 관련 정보
- 수면 여부 및 관련 정보
- 산부인과 관련 정보
- 연구 참여 경로 및 동기

(2) 수족냉증 / 기타 신체기관 병력 및 약물력 조사

수족냉증 관련 병력 및 약물력 조사: 방문0에 실시

수족냉증 관련 항목 (해당항목)

- 수족냉증 진단 여부
- 수족냉증 이환 기간 (개월)
- 수족냉증 관련 기존 치료력
  (약물치료 / 한방치료(침/뜸/한약 등) / 운동요법 / 정신치료 / 기타 보조 약물)
- 수족냉증 관련 복용 중인 약물 및 치료
- 기타 신체기관 관련 병력 및 약물력 항목
- 일반신체검사 항목: 매 방문마다 실시

감각기관, 근골격계, 내분비계, 다발계, 비뇨기계, 생식기계, 소화기계, 심혈관계, 신경계, 피부계, 정

신장애, 혈액 및 림프계, 호흡기계 등의 병력 및 약물력에 대해 문진한다. 1차 방문 시 검사 상 발견된 유의할 만한 사항은 증례기록표의 기타 신체기관 관련 항목 란에 기록하고, 연구 시술의 개시 후 이상반응의 정의에 부합되는 이학적 소견상의 유의할 만한 사항이 발견된 경우에는 이상반응 증례기록지에 기록한다.

(3) 시각적 상사척도(Visual Analogue Scale : VAS)

매 방문마다 평가한다. 방문 시 수부냉증 및 족부냉증 정도에 대한 VAS를 연구자의 도움 하에 연구대상자가 작성한다.

(4) 변증설문지 및 체질진단 설문지(KS-15)

방문0에 실시한다.

연구 책임자나 시험담당자(한의사) 2인이 평가를 한다.

① 평가자는 변증 해당 항목을 확인 후, 사진을 통해서 연구대상자가 가지고 있는 상태항목의 정도를 가장 적당한 항목에 ○ 표기한다.

② 평가자는 해당하는 변증유형에 ○ 표기한다.

(5) 활력징후 및 신장 체중 측정

활력징후에 대한 평가는 매 방문마다 실시한다. 활력징후는 혈압, 맥박수, 체온을 측정한다. 신장은 방문0에만 측정하고, 체중은 방문0, 10, 11에 실시한다.

(6) 선정/배제기준 적합성 판정

방문0에서 대상자 동의, 인구학적•사회학적 정보, 병력 및 약물력 조사, 실험실 검사, 임신검사 및 흉부 X-ray 촬영, 심전도 검사 및 냉증 진단기준 부합 여부 확인을 위한 써모미터 측정을 시행한다. 결과를 종합하여 적합성 여부를 최종 판단하고, 이를 참여자에게 전화로 통보하여 Visit 1 일정을 정한다.

(7) 병력 및 치료력 변화 여부 확인

Visit 0 (Screening) 방문 이후 조사되었던 사항 이외에 병력 및 치료력에 변화가 있는지 여부에 대하여 방문시마다 조사한다. 방문0의 약물력 검사에서 확인된 내용과 비교하여 변화된 사항에 대하여 조사하고 만일 병용약물에 변화가 있다면 이를 증례기록지에 자세하게 기록한다.

(8) 임상검사항목

서면동의서를 취득한 대상자를 대상으로 방문0에서 12-lead EKG, 흉부 X-ray와 다음 사항 검사를 실시한다

① 혈액학적 검사 : WBC, RBC, Hemoglobin, Platelet

② 혈액생화학적 검사 : BUN, Creatinine, AST, ALT, r-GTP, Glucose

③ 갑상선기능검사: free T4, TSH

④ 소변 및 요침사 검사 : Blood, Urobilinogen, Bilirubin, Ketone, Protein, Nitrate, Glucose, pH, S.G, LEU, WBC, RBC

⑤ 임신검사(가임기 여성) : Urine HCG

단, Visit 10에는 다음과 같은 검사를 실시한다.
① 혈액학적 검사 ; WBC, RBC, Hemoglobin, platelet
② 혈액생화학적검사 : BUN, Creatinine, AST, ALT, r-GTP
Visit 0의 Screening 중 선정/제외기준에서 측정하여 평균한 수축기 혈압 180mmHg 또는 이완기 혈압 100mmHg 이상, 성인여자(비임신) Hemoglobin 7g/dL 미만, WBC 11,000/mm^3 이상, Random plasma glucose 50mg/dL미만 혹은 250mg/dL 이상, AST·ALT 각 100 IU/L 이상, Creatinine 2.0mg/dL 이상, 요중 hCG 양성, 흉부 X선 사진상 비활동성 결핵 이외의 폐결핵 병변이나 기흉이 있는 경우, 심전도상 치료가 필요한 부정맥, 허혈성 심질환 등 심장질환이 있는 경우에 제외한다.
상기 검사상 이상소견이 없으며, 환자가 호소하는 증상 등을 고려하여 수부 족부냉증으로 판단될 경우 연구 참여를 결정한다.
상기 검사는 임상시험에 참여하는 각 의료기관의 검사실에서 시행하며, 한방병원에서 상기 검사를 양방의료기관의 검사실에 의뢰하여 검사를 실시한다.

4) 무작위 배정
침치료군, 전침치료군 및 무처치대조군으로 무작위 배정한다.

5) 기저치 검사
무작위 배정된 연구대상자를 대상으로 시각적상사척도(Visual Analogue Scale : VAS), 체온 (써모미터 측정), 삶의 질 평가 설문지(WHOQOL-BREF), 삶의 질 평가 EQ-5D, 냉증진단 설문지, 체중, 활력증후에 대한 기저치를 측정한다. 피험자의 설문조사는 참여 연구원중 1인을 지정하고, 이 연구원은 침치료군이나 전침치료군에 대한 침시술에 관여하지 않도록 하여, 평가자 bias를 최소화하도록 한다. 평가자는 피험자의 시술여부에 대해 문진하지 않도록 한다.

6) 자침 및 전침치료 방법 :
침치료군과 전침치료군에 대한 시술은 설문 및 평가에 참여하지 않은 연구자나 시험담당자가 시술한다.

(1) 침치료군

침치료군은 치료기간 5주 동안 주 2회, 총 10회의 침 시술을 받는 것을 표준으로 한다. 0.25 x 30 mm 규격의 멸균침(동방메디컬, 대한민국)을 사용하여, 피험자의 좌우의 태충(LR3), 삼음교(SP6), 합곡(LI4), 외관(TE5) 총 8곳에, 피하 10~25 mm의 깊이로 자침한다.

(2) 전침치료군

전침치료군은 치료기간 5주 동안 주 2회, 총 10회의 전침 시술을 받는 것을 표준으로 한다. 0.25 x 30 mm 규격의 멸균침(동방메디컬, 대한민국)을 사용하여, 피험자의 좌우의 태충(LR3), 삼음교(SP6), 합곡(LI4), 외관(TE5) 총 8곳에, 피하 10~25 mm의 깊이로 자침하고, 태충(LR3)은 삼음교(SP6)에, 합곡(LI4)은 외관(TE5)에 전침기(STN-330, 스트라텍)에 연결된 전용클립으로 각각 연결한다(적색, 검은 색 구분없음). 전기 자극 빈도는 2 Hz, 자극시간은 15분으로 설정하고, 강도는 피험자가 자극을 인지하되 통증을 느끼지 않고, 편안하게 느끼는 정도로 한다. 전기자극이 끝나면 발침하고, 발침 부위는 소독한다.

(3) 무처치대조군

침치료와 전침 시술을 제공하지 않는다.

7) 연구종료

침치료 시작 5주후 이상반응 발현여부와 순응도를 평가하고, 시각적 상사척도(VAS), 체온 (써모미터 측정), 삶의 질 평가 설문지(WHOQOL-BREF), 삶의 질 평가 EQ-5D, 냉증진단 설문지, 활력징후, 체중측정, 실험실 검사, 병력 및 약물 투여력 변화 확인, 맹검 유지 조사 등을 실시한다.

8) 추적조사

임상시험 종료 4주 후 필요시 이상반응 발현여부를 평가하고, 시각적 상사척도(VAS), 체온 (써모미터 측정), 삶의 질 평가 설문지(WHOQOL-BREF), 삶의 질 평가 EQ-5D, 냉증진단 설문지, 활력징후, 체중측정, 병력 및 약물 투여력 변화를 확인한다.

라. 병용요법 및 주의 사항

1) 시험기간 중 침 치료 이외의 약물 및 기타요법은 실시하지 않는 것을 원칙으로 한다.

2) 시험기간 중 다음 아래 약물을 추가 복용하지 말아야 한다.
예) 항응고제, 향정신성 약물, 항우울 약물, 갑상선기능항진증 관련 약물, 건강기능식품 (홍삼), OTC 중 혈류개선제, 그 외에 수부 족부냉증에 영향을 줄 가능성이 있는 약물 등.

3) 다만, 필요한 경우에는 다음의 기준에 따른 시험담당자의 판단에 의해 실시될 수 있다. 시험담당자의 판단 없이 투약한 약물 및 요법이 본 임상시험의 평가항목에 영향을 줄 수 있다고 예상되는 경우 이 연구대상자는 탈락하게 된다. (중지 탈락 기준 참조).
예) 항응고제, 향정신성 약물, 항우울 약물, 갑상선 기능 항진증 관련 약물, 건강기능식품 (홍삼), OTC 중 혈류개선제, 그 외에 수부 족부냉증에 영향을 줄 가능성이 있는 약물 등.

4) 투여한 모든 약물 및 투여 사유를 반드시 CRF에 기재하고 연구담당자가 서명한다.

〈임상시험 흐름도〉

연구대상자 모집 및 서면동의서 작성 (방문0)

↓

선별검사
활력징후, 신장, 체중, 연구 참여 경로 및 동기, 인구학적 사회학적 조사, 산부인과적 정보력, 병력 및 약물 투여력 조사, 일반신체검사, 써모미터 측정, 수부냉증 및 족부냉증 VAS, 변증 설문지, 실험실적 검사, Chest X-ray, ECG, 선정 제외기준 판단

↓

무작위 배정
침치료군, 전침치료군, 무처치대조군 무작위 배정

↓

Baseline (0주) 평가 (방문1)
활력징후, 체중, 병력 및 약물 투여력 조사 및 변화확인, 체질진단 설문(KS-15) 일반신체검사, 써모미터 측정, 수부냉증 및 족부냉증 VAS, 냉증진단 설문지, 삶의 질 평가 설문지, 삶의 질 평가 EQ-5D, 침치료군 및 전침치료군 치료, 다음 방문일 지정

↓

침 또는 전침 치료 (방문 2 ~9)
5주간 침치료군 및 전침치료군 치료, 활력징후, 병력 및 약물투여력 조사, 일반신체검사, 써모미터 측정, 수부냉증 및 족부냉증 VAS, 이상반응 검사 및 유무확인, 다음 방문일 지정

↓

5주째 평가 (방문 10)
활력징후, 체중, 병력 및 약물 투여력 조사 및 변화 확인, 일반신체검사, 써모미터 측정, 수부냉증 및 족부냉증 VAS, 이상반응 검사 및 유무 확인, 삶의 질 평가 설문지, 삶의 질 평가 EQ-5D,, 냉증진단 설문지, 실험실적 검사, 다음 방문일 지정

↓

4주간 일상 생활

↓

9주째 평가 (방문 11)
활력징후, 체중, 병력 및 약물 투여력 조사 및 변화 확인, 일반신체검사, 써모미터 측정, 수부냉증 및 족부냉증 VAS, 삶의 질 평가 설문지(WHOQOL-BREF), 삶의 질 평가 EQ-5D,, 냉증진단 설문지, 이상반응 검사 및 유무 확인

↓

데이터 정리 및 통계처리

10. 관찰 및 검사 항목

방문	스크리닝 (D -7~0)	Treatment period 방문 1 (D 1)	방문 2~9	방문 10 (D 35±3)	방문11 (D 63±3)
서면동의서 취득	●				
스크리닝 번호 부여	●				
Chest X-ray & EKG[8]	●				
선정/제외기준[9]	●				
무작위 배정		●			
무처치대조군		■		■	■
치료순응도 평가			○	○	○
연구대상자의 중도탈락 여부			○	○	○
활력징후	●	●	○	●	●
신장, 체중, 신체질량지수(BMI)	●			●(체중만)	●(체중만)
연구 참여 경로 및 동기	●				
인구학적 사회학적 조사, 산부인과적 정보력[10]	●				
병력 및 약물 투여력 조사[11] 및 변화 확인	●	●	○	●	●
체질진단 설문(KS-15)	●				
검사					
일반신체검사	●	●	○	●	●
써모미터 측정[12]	●	●	○	●	●
수부냉증 및 족부냉증 VAS	●	●	○[13]	●	●
이상반응 검사 및 유무 확인			○	●	●
변증 설문지	●				●
냉증진단 설문지		●		●	●
삶의 질 평가 설문지(WHOQOL-BREF)		●		●	●
삶의 질 평가 EQ-5D		●		●	●
실험실적 검사	●[14]			○	
경혈침 치료 또는 전침치료		○	○	○	
다음 방문일 지정		●	○	●	
시험자 서명	●	●	●	●	●

● 세 군 모두 실시
○ 침치료군, 전침치료군만 실시
■ 무처치대조군 방문일

8) Screening 시에는 흉부 X-ray, 심전도 실시함
9) Screening 시에 실시함
10) 연령, 직업, 소화력, 운동여부, 흡연, 음주, 수면, 시험참여 동기 등을 조사함.
11) 수족냉증 관련 병력 및 약물력 포함
12) 방문시마다 전대퇴 중심부(ST32)과 족배부 전면부(LR3) 및 수장부위(PC8)와 전완부 중심부(LU4)의 양측 온도 측정함
13) 침치료군과 전치치료군은 방문 4, 방문 8에 수부냉증 및 족부냉증 VAS를 추가로 측정함.
14) Screening 시에는 혈액학적검사(WBC, RBC, Hemoglobin, platelet), 혈액생화학적검사(BUN, creatinine, AST, ALT, r-GTP, glucose), 갑상선기능검사(free T4, TSH), 소변 및 요침사검사, 임신검사(Urine HCG)를 실시함. D35(방문10)방문 시에는 혈액학적 검사(WBC, RBC, Hg, platelet), 혈액생화학적검사 (BUN, Creatinine, AST, ALT, r-GTP) 시행함.

가. 방문 0 (Screening)

1) 연구대상자모집

각 시험실시 기관의 방문환자 혹은 옥외 광고를 통한 공개모집 등의 경로로 자원자를 모집한다. 모집 인원은 경쟁적으로 모집한다.

2) 시험동의 및 연구대상자식별코드 부여

(1) 연구책임자나 연구담당자는 연구대상자에게 연구대상자 동의 설명서 및 개인정보에 관하여 IRB에 승인된 설명서로 임상시험에 대해 충분히 설명한다.

(2) 연구대상자는 충분히 설명을 듣고, 시험동의여부에 대하여 고민하게 한 후 동의할 경우 본인 자필로 동의서에 동의한 연월일, 성명을 기입하고 서명을 한다. 동의 설명을 담당한 연구책임자나 담당자도 연월일, 성명을 기입하고 서명을 한다.

※ 주의점

○ 연구 책임자나 연구담당자는 연구대상자의 동의서 서명, 성명, 연월일이 일치하는지 복사 동의서 제공 전에 꼭 확인해야 한다.

○ 연구대상자가 잘못 기입하여 동의서를 수정하려고 하면, 새 동의서에 다시 작성을 우선 원칙으로 합니다. 그렇지 않은 경우 검은색으로 한줄 긋고, 옆에 오기, 수정날짜, 본인 이름을 기입한다.

overwrite 또는 수정액 사용은 절대 금기입니다.

a. 정정방법 : 한 줄 긋고, '사유/수정일/서명(혹은 이니셜)'를 기록합니다.

오기 08-03-24 JBH<br>146/~~86~~ 88

○ 동의서 기입날짜가 의무기록상 내원일과 동일한지 꼭 확인하며, 동의서 날짜는 절대로 임상시험의 실제 연구대상자 식별코드 부여한 날짜의 이후가 될 수가 없습니다.

(3) 동의 설명서와 동의서 사본1부을 연구대상자에게 주고, 자필로 "동의서 사본을 받았다"는 내용과 당일 연월일, 성명, 사인을 받는다.

(4) Screening용 연구대상자식별코드를 부여한다.

S[|]-[| |]

① S : Screening

② 첫 두칸

: 세명대학교 충주한방병원에서 연구대상자 모집시, 대문자로 SC 각각 한칸씩 표기

S[S | C]

③ 해당 의료기관별 약어는 다음과 같다.
□ 세명대학교 충주한방병원 (SC)
□ 상지대학교 부속한방병원 (SJ)
□ 동국대학교 일산한방병원 (DG)

④ 하이픈 다음 세칸
각 의료기관별 내원하여 동의서에 서명한 순서에 따라 번호를 붙인다.
예) 첫 번째 : 001
예) 세명대학교 충주 한방병원에서 15번째 동의서에 서명한 한 연구대상자의 Screening 번호는 다음과 같다

S	S	C	-	0	1	5

3) 대상자 선정

대상자가 임상시험에 참여하기를 희망하면, 자세히 임상시험에 대해 설명후 자발적으로서면 동의서에 서명하도록 하고, 스크리닝 번호를 부여한다.

(1) 활력징후와 신장, 체중 기록
활력징후에 대한 평가는 매 방문마다 실시한다. 활력징후는 혈압, 맥박수, 체온을 측정한다.

(2) 인구학적, 사회학적 정보
연령 직업 소화 운동 흡연 음주 수면

(3) 연구 참여 경로 및 동기

(4) 산부인과적정보
월경력
초경시기, 평균 월경주기, 평균 월경지속기간, 월경 중 덩어리 유무, 월경색, 월경통 유무
대하력
대하 유무, 대하색
임신력
과거 임신 횟수, 가임 여부, 하복부 냉감 여부

(5) 수족냉증 / 기타 신체기관 병력 및 약물력 조사
○ 수족냉증 관련 병력 및 약물력 조사: 방문 1에 실시
수족냉증 관련 항목 (해당항목)
- 수족냉증 진단 여부
- 수족냉증 이환 기간 (개월)
- 수족냉증 관련 기존 치료력
  (약물치료 / 한방치료(침/뜸/한약 등) / 운동요법 / 정신치료 / 기타 보조 약물)
- 수족냉증 관련 복용 중인 약물 및 치료

(6) 기타 신체기관 관련 병력 및 약물력 항목- 방문 1에 실시

일반신체검사 항목: 매 방문마다 실시

감각기관, 근골격계, 내분비계, 다발계, 비뇨기계, 생식기계, 소화기계, 심혈관계, 신경계, 피부계, 정신장애, 혈액 및 림프계, 호흡기계 등의 병력 및 약물력에 대해 문진한다. 1차 방문 시 검사 상 발견된 유의할 만한 사항은 증례기록표의 기타 신체기관 관련 항목 란에 기록하고, 연구 시술의 개시 후 이상반응의 정의에 부합되는 이학적 소견상의 유의할 만한 사항이 발견된 경우에는 이상반응 증례기록지에 기록한다.

(7) 써모미터 측정

24±℃, 습도 40~60%의 방안에서 양쪽 팔꿈치 위쪽으로 10cm 이상을 노출시키고, 하지의 의복을 무릎까지 올린 상태에서 10분간 앉아서 안정을 취한 후, 전대퇴 중심부(ST32)과 족배부 전면부(LR3), 수장부위(PC8)와 전완부 중심부(LU4)에서 약 3 ㎝ 거리에서 비접촉식 적외선 써모미터를 사용하여 3회 연속 연속 측정하고, 최고값과 최저값을 제외한 중간값을 기록한다.

(8) 수부냉증 및 족부냉증 시각적 상사척도(Visual Analogue Scale : VAS)

수부냉증 및 족부냉증 정도에 대한 VAS를 연구자의 도움 하에 연구대상자가 작성한다.

VAS에 연구대상자는 본인의 수부냉증 및 족부냉증 정도를 I로 표시를 한다.

O, V, 구두로 표시하지 않는다.(정확히 표현이 안 됨)

침치료 시작 전과 치료 종료 후 0부터 10까지의 숫자가 표시된 가로방향 직선을 사용하여 대상자가 자신의 피로 정도에 해당하는 숫자를 주어진 숫자 중에 선택하도록 한다. 직선의 왼쪽 끝에는 '냉증 없음'이라는 설명을, 오른쪽 끝에는 '상상할 수 있는 가장 심한 냉증'라는 설명을 추가한다. 점수는 환자가 선택한 숫자 그대로를 점수로 삼는다.

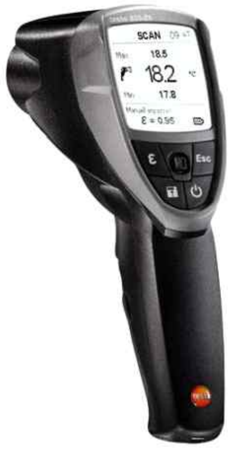

	testo 835-T1	testo 835-T2	testo 835-H1
적외선			
초점비	50:1(측정 대상과의 거리가 표준 2.0m 일 때) + 센서의 개구부 지름(24mm)		
측정점	4 포인트 레이저		
광학 범위	8 ~ 14 μm		
측정 범위	-30 ~ +600 ℃	-10 ~ +1500 ℃	-30 ~ +600 ℃
정확도<br>±1 digit	±2.5 ℃(-30 ~ -20.1 ℃)<br>±1.5 ℃(-20 ~ -0.1 ℃)<br>±1 ℃(+0 ~ +99.9 ℃)<br>측정값의 ±1%(나머지 범위)	±2 ℃ 또는 측정값의 ±1%	±2.5 ℃(-30 ~ -20.1 ℃)<br>±1.5 ℃(-20 ~ -0.1 ℃)<br>±1 ℃(+0 ~ +99.9 ℃)<br>측정값의 ±1%(나머지 범위)
분해능	0.1 ℃	0.1 ℃ (-10 ~ +999.9 ℃)<br>1 ℃ (+1000 ~ +1500 ℃)	0.1 ℃
K타입(NiCr-Ni)			
측정범위	-50 ~ +600 ℃	-50 ~ +1000 ℃	-50 ~ +600 ℃
정확도<br>±1 digit	±(-0.5 ℃ 측정값의 +0.5%)		
분해능	0.1 ℃		
공통 기술데이터			
방사율	0.10 ~ 1.00 (0.01씩 범위조절 가능)		
방사율값 저장개수	20개 저장 가능		
레이저 측정점	On / Off		
메모리	200개 저장 가능		
알람(한계값)	IR 온도, TC 온도		
알람 신호	시각, 청각		
작동온도	-20 ~ +50 ℃		
보관온도	-30 ~ +50 ℃		
재질/하우징	ABS + PC		
크기	193 x 166 x 63 mm		
무게	514 g		
배터리 타입	AA타입 배터리 3개(또는 PC에서 USB로 충전 가능)		
배터리 수명	25시간(25 ℃에서 레이저 및 백라이트 없을 시)<br>10시간(25 ℃에서 백라이트 없을 시)		
디스플레이	도트 매트릭스		
자동꺼짐<br>(측정중/USB연결중일 때)	백라이트: 30초<br>측정기: 120초		
표준	EN 61326-1:2006		
보증기간	2년		

Screening 방문 당시, 써모미터(Thermometer)로 측정한 수장부위(PC8)와 전완부 중심부(LU4)의 온도차가 0.3℃ 이상 또는 전대퇴 중심부(ST32)과 족배부 전면부(LR3)의 온도차가 2℃ 이상이며 수부 또는 족부 냉증의 시각적 상사척도(Visual Analogue Scale : VAS)가 4이상인 자에 한하여 등록을 진행한다.

(9) 변증설문지
방문0에 실시한다.
방문0에서 연구 책임자나 담당자(한의사) 2인이 평가를 한다.
1) 평가자는 변증 해당 항목을 확인 후 연구대상자가 가지고 있는 상태항목의 정도를 가장 적당한 번호에만 ○ 표기한다.
2) 평가자는 해당하는 변증유형에 ○ 표기한다.

(10) 체질진단 설문(KS-15) 실시
방문 0에 실시한다.
방문 0에서 연구 책임자나 담당자가 실시한다.

(11) 실험실적 검사
방문0에서 12-lead EKG, 흉부 X-ray와 다음 사항 검사를 실시한다.
① 혈액학적 검사 : WBC, RBC, Hemoglobin, Platelet;
② 혈액생화학적 검사 : BUN, creatinine, AST, ALT, r-GTP, glucose
③ 소변 및 요침사 검사 : Blood, Urobilinogen, Bilirubin, Ketone, Protein, Nitrate, Glucose, pH, S.G, LEU, WBC, RBC
④ 임신검사(가임기 여성) : Urine HCG;
⑤ 갑상선기능검사: free T4, TSH;

(12) 선정/배제기준 적합성 판정
방문0에서 대상자 동의, 인구학적•사회학적 정보, 병력 및 약물력 조사, 검사실 검사, 임신검사 및 흉부 X-ray 촬영, 심전도 검사 및 냉증 진단기준 부합 여부 확인을 위한 써모미터 측정을 시행한다. 결과를 종합하여 적합성 여부를 최종 판단하고, 검사 14일 이내에 참여자에게 전화로 통보하여 Visit 1 일정을 정한다.

나. 방문 1 (D1)

(1) 활력징후 및 체중 측정

(2) 질환/의약용품/치료 확인(병력 및 약물 투여력 변화 확인)
Screening 이후 방문1까지 새롭게 발생한 질환, 의약품 추가 복용, 추가 치료 여부를 조사한다.
예라고 기록한 경우 질환/의약용품/치료 CRF 서식에 기록을 남긴다.

(3) 일반신체 검사

내원 당시 CRF의 일반 신체검사 서식에 있는 질환이 있는지 파악한다.
있으면 유□에 V표하고, 아래 이상소견에 기록한다.
없으면 무□에 V표한다.

(4) 써모미터 측정
방문시마다 치료 전후 전대퇴 중심부(ST32)과 족배부 전면부(LR3), 수장부위(PC8)와 전완부 중심부(LU4) 의 양측 온도를 ,3 ㎝ 거리에서 비접촉식 적외선 써모미터를 사용하여 3회 연속 연속 측정하고, 최고값과 최저값을 제외한 중간값을 기록한다.

(5) 수부냉증 및 족부냉증 시각적 상사척도(Visual Analogue Scale : VAS)
수부냉증 및 족부냉증 정도에 대한 VAS를 연구자의 도움 하에 연구대상자가 작성한다.
VAS에 연구대상자는 본인의 수부냉증 및 족부냉증 정도를 I로 표시를 한다.
O, V, 구두로 표시하지 않는다.(정확히 표현이 안 됨)

(6) 냉증 진단 설문지
환자가 기입할 항목은 기입하게 한다.
한의사가 측정할 항목은 반드시 한의사가 진행한다.

(7) 한국판 세계보건 기구 삶의 질 척도(단축형) (WHOQOL-BREF)와 삶의 질 평가 EQ-5D
가장 적당한 번호에만 ○ 표기한다.
의무기록상 문서 의무기록은 설문지를 보관하며, 전자 의무기록인 경우는 그 점수나 해당내용 또는 항목별 번호를 기록한다. 어렵다면 임상시험용 서면의무기록파일을 만들어 저장한다.

(8) 무작위 배정
무처치대조군, 침치료군 및 전침치료군으로 무작위 배정한다.
미리 정해진 무작위배정표의 번호를 의무기록과 CRF에 기록한다.

(9) 침 또는 전침치료
설문조사에 참여하지 않은 연구원이 무작위 배정된 침치료군 또는 전침치료군에 각각에 해당하는 방법으로 시술한다.

(10) 다음 방문일 지정
침치료군과 전침치료군은 1주간 2회 치료를 원칙으로, 시술후 4±3일 이내에 방문하도록 다음 방문 일을 지정하여 연구대상자에게 알려준다. 무처치대조군은 별도의 치료없이 35±3일(방문 10)되는 날을 다음 방문 일로 지정하여 연구대상자에게 알려준다.

다. 방문 2 ~ 방문 9

(1) 연구대상자의 중도탈락

연구대상자의 protocol 위반사유가 있을시 임상시험참여를 중단하고 임상시험종결란(End of

Study)에 기록한다. 위반사유가 없을시 임상시험을 진행한다.
 임상연구의 중도탈락은 다음을 기준으로 한다. 다음 조건의 하나라도 해당될 때 담당주치의의 판단에 의해 시험에서 제외한다.

가. 예측되는 치료 순응도가 70%미만인 경우 연구대상자는 탈락하는 것을 원칙으로 한다.
 방문 지정일로부터 ±3일 이내에 방문하지 않으면 방문하지 않은 것으로 간주한다.
나. 연구기간 중 임신했을 때
다. 사고 등의 재해나 타 질환의 발병으로 수술 또는 입원치료가 필요한 경우
라. 임상시험연구대상자가 거부한 경우
마. 금지 병용 약물 및 요법을 실시했을 때
 예) 항응고제, 향정신성 약물, 항우울 약물, 갑상선기능항진증 관련 약물, 건강기능식품 (홍삼), OTC 중 혈류개선제, 그 외에 수부 족부냉증에 영향을 줄 가능성이 있는 약물 등.
바. 수부 또는 족부냉증 증상의 악화로 표준 치료가 필요한 경우.
사. 중대한 이상반응으로 임상시험을 지속하기 곤란하다고 판단되는 경우
아. 기타 연구를 지속하기 힘든 부득이한 사유가 발생했거나 임상시험 결과에 영향을 끼쳤을 것으로 임상시험책임자가 판단한 경우

(2) 활력징후 측정
 활력 징후를 측정 기록한다.
 활력 징후로 체온, 수축기혈압, 이완기 혈압, 맥박수를 확인 기록한다.

(3) 질환/의약용품/치료 확인(병력 및 약물 투여력 변화 확인)
 방문2에서 방문9까지 기간 동안 새롭게 발생한 질환이 있는지, 의약품 등을 추가로 복용하였는지, 다른 치료를 한 적이 있는지를 확인한다.
 있다면 질환/의약용품/치료 CRF 서식에 기록을 남긴다.

(4) 일반 신체검사
 내원 당시 CRF의 일반 신체검사 서식에 있는 질환이 있는지 파악한다.
 있으면 유□에 V표하고, 아래 이상소견에 기록한다.
 없으면 무□에 V표한다.

(5) 침 또는 전침치료
 설문조사에 참여하지 않은 연구원이 무작위 배정된 침치료 또는 전침치료군에 대해 적합한 치료를 실시한다.

(6) 써모미터 측정
 방문시마다 치료 전후 전대퇴 중심부(ST32)과 족배부 전면부(LR3), 수장부위(PC8)와 전완부 중심부(LU4) 의 양측 온도를, 3 ㎝ 거리에서 비접촉식 적외선 써모미터를 사용하여 3회 연속 연속 측정하고, 최고값과 최저값을 제외한 중간값을 기록한다.

(7) 수부냉증 및 족부냉증 시각적 상사척도(Visual Analogue Scale : VAS)

수부냉증 및 족부냉증 정도에 대한 VAS를 연구자의 도움 하에 연구대상자가 작성한다.
VAS에 연구대상자는 본인의 수부냉증 및 족부냉증 정도를 I로 표시를 한다.
O, V, 구두로 표시하지 않는다.(정확히 표현이 안 됨)

(8) 이상반응 검사 및 유무 확인
침치료 기간 중에 발생한 이상반응 여부를 확인한다.
이상반응이 있으면, CRF의 부작용 보고서 작성한다.
이상반응 보고 절차는 이상반응 보고 SOP 및 침치료 관리방법에 관한 표준작업지침서에 따른다.

(9) 다음 방문일 지정
1주간 2회 침치료를 원칙으로 하며, 4±3일 이내에 방문하도록 다음 방문 일을 지정하여 연구대상자에게 알려준다.

라. 방문 10 (D35±3)

(1) 연구대상자의 중도탈락
치료 순응도를 계산한다.
예) 치료 순응도 (%) = 연구대상자의 실제 침 치료 / 10회 침 치료
침치료 순응도 : □□.□%

(2) 활력징후 및 체중 측정
활력 징후 및 체중을 측정 기록한다.
활력 징후로 체온, 수축기혈압, 이완기 혈압, 맥박수를 확인 기록한다.

(3) 질환/의약용품/치료 확인(병력 및 약물투여력 변화 확인)
방문9 이후 새롭게 발생한 질환이 있는지, 의약품 등을 추가로 복용하였는지, 다른 치료를 한 적이 있는지를 확인한다. 있다면 질환/의약용품/치료 CRF 서식에 기록을 남긴다.

(4) 일반 신체검사
내원 당시 CRF의 일반 신체검사 서식에 있는 질환이 있는지 파악한다.
있으면 유□에 V표하고, 아래 이상소견에 기록한다.
없으면 무□에 V표한다.

(5) 침 또는 전침치료
설문조사에 참여하지 않은 연구원이 무작위 배정된 침치료 또는 전침치료군에 대해 적합한 치료를 실시한다.

(6) 써모미터 측정
치료 전후 전대퇴 중심부(ST32)과 족배부 전면부(LR3), 수장부위(PC8)와 전완부 중심부(LU4)의 양측 온도를, 3 ㎝ 거리에서 비접촉식 적외선 써모미터를 사용하여 3회 연속 연속 측정하고, 최고값과 최저값을 제외한 중간값을 기록한다.

(7) 수부냉증 및 족부냉증 시각적 상사척도(Visual Analogue Scale : VAS)
수부냉증 및 족부냉증 정도에 대한 VAS를 연구자의 도움 하에 연구대상자가 작성한다.
VAS에 연구대상자는 본인의 수부냉증 및 족부냉증 정도를 I로 표시를 한다.
O, V, 구두로 표시하지 않는다.(정확히 표현이 안 됨)

(8) 이상반응 검사 및 유무 확인
임상시험 기간 중에 발생한 이상반응 여부를 확인한다.
이상반응이 있으면, CRF의 부작용 보고서 작성한다.
이상반응 보고 절차는 이상반응 보고 SOP 및 침치료 관리방법에 관한 표준작업지침서에 따른다.

(9) 한국판 세계보건 기구 삶의 질 척도(단축형) (WHOQOL-BREF)와 삶의 질 평가 EQ-5D
가장 적당한 번호에만 ○ 표기한다.
의무기록 상 문서 의무기록은 설문지를 보관하며, 전자 의무기록인 경우는 그 점수나 해당내용 또는 항목별 번호를 기록한다. 어렵다면 임상시험용 서면의무기록파일을 만들어 저장한다.

(10) 냉증 진단 설문지
환자가 기입할 항목은 기입하게 한다.
한의사가 측정할 항목은 반드시 한의사가 진행한다.

(11) 실험실적 검사
일반혈액학 검사로, WBC, RBC, Hemoglobin, Platelet을 검사한다.
혈액화학검사로 BUN, Creatinine, AST, ALT, r-GTP를 검사 기록한다.
임상시험에 참여하는 각 의료기관의 검사실에서 시행한다.
한방병원에서 상기 검사를 양방의료기관의 검사실에 의뢰하여 검사를 실시한다.

(12) 다음 방문일 지정
4주±7일 이내에 다음 방문일을 지정하여 연구대상자에게 알려준다.

마. 방문 11 (D63±7)

(1) 활력징후 및 체중 측정
활력 징후 및 체중을 측정 기록한다.
활력 징후로 체온, 수축기혈압, 이완기 혈압, 맥박수를 확인 기록한다.

(2) 질환/의약용품/치료 확인(병력 및 약물투여력 변화 확인)
방문10에서 방문11까지 기간 동안 새롭게 발생한 질환이 있는지, 의약품 등을 추가로 복용하였는지, 다른 치료를 한 적이 있는지를 확인한다.
있다면 질환/의약용품/치료 CRF 서식에 기록을 남긴다.

(3) 일반 신체검사
내원 당시 CRF의 일반 신체검사 서식에 있는 질환이 있는지 파악한다.
있으면 유□에 V표하고, 아래 이상소견에 기록한다.
없으면 무□에 V표한다.

(4) 써모미터 측정
방문시 전대퇴 중심부(ST32)과 족배부 전면부(LR3), 수장부위(PC8)와 전완부 중심부(LU4) 의 양측 온도를, 3 ㎝ 거리에서 비접촉식 적외선 써모미터를 사용하여 3회 연속 연속 측정하고, 최고값과 최저값을 제외한 중간값을 기록한다.

(5) 수부냉증 및 족부냉증 시각적 상사척도(Visual Analogue Scale : VAS)
수부냉증 및 족부냉증 정도에 대한 VAS를 연구자의 도움 하에 연구대상자가 작성한다.
VAS에 연구대상자는 본인의 수부냉증 및 족부냉증 정도를 I로 표시를 한다.
O, V, 구두로 표시하지 않는다.(정확히 표현이 안 됨)

(6) 이상반응 검사 및 유무 확인
4주 동안 발생한 이상반응 여부를 확인한다.
이상반응이 있으면, CRF의 부작용 보고서 작성한다.
이상반응 보고 절차는 이상반응 보고 SOP 및 침치료 관리방법에 관한 표준작업지침서에 따른다.

(7) 한국판 세계보건 기구 삶의 질 척도(단축형) (WHOQOL-BREF)와 삶의 질 평가 EQ-5D
가장 적당한 번호에만 ○ 표기한다.
의무기록 상 문서 의무기록은 설문지를 보관하며, 전자 의무기록인 경우는 그 점수나 해당내용 또는 항목별 번호를 기록한다. 어렵다면 임상시험용 서면의무기록파일을 만들어 저장한다.

(8) 냉증 진단 설문지
환자가 기입할 항목은 기입하게 한다.
한의사가 측정할 항목은 반드시 한의사가 진행한다.

바. 임상시험 종결 End of Study

(1) 임상시험을 최종 완료했을 경우 예에 V표 하고, 종료일자를 기록한다.
예) ▶ 임상시험이 Protocol 에 맞게 완료되었습니까?
□ 예 → 종료일자 20□□년 □□월 □□일

(2) 임상시험 중간에 중지했거나 순응도에서 탈락했을 경우에 CRF 서식 [임상시험 종결]에 아니오로 V표 하고 중지 또는 탈락일자를 작성하고, 중지 또는 탈락원인 항목에 V 표로 표시한다.

예) □ 아니오 → 중지 또는 탈락일자 20□□년 □□월 □□일

▶ 임상시험 중지 또는 탈락원인

□ 선정/제외기준 위배 Violation of Inclusion/Exclusion Criteria

□ 임상시험전 미발견 전신 질환
Unidentified systemic disease before clinical trial

□ 이상반응 Adverse reaction

□ 병발증 (중재와 관련 없음) Intercurrent illness (not intervention related)

□ 대상자의 중지 요청(부작용 외 이유)
Subject request for withdrawal (other than adverse reaction)

□ 담당자가 종료 지시 Investigator indicated termination

□ 임상시험계획 비준수로 제외됨
Exclusion from study due to non-adherence to protocol

□ 추적실패 Pursuit failure

□ 기타 other( )

(3) 경과 또는 종합의견에 기록할 사항이 있으면 기록한다.
(4) 연구책임자는 CRF를 최종적으로 확인 후에 확인한 날짜를 기입하고, 서명한다.

사. CRF 완료 및 증례결론 최종 서명

(1) CRF가 최종 작성되면, 연구책임자는 근거문서(의무기록)와 일치하는 지 확인하고, 일치하지 않는 부분과 기록 누락, CRF 체크 누락여부를 확인한다.
(2) 모니터링 등을 통해 누락여부를 확인한다.
(3) 근거문서와 CRF가 일치함을 확인하고, 동의서 서명일, 성명, 사인이 오기 없이 작성되었는지를 확인한다.
(4) 시험자와 임상시험 책임자는 증례기록지의 적합성 등을 보증하기 위해 증례결론에 최종 서명을 하여 CRF 작성을 최종적으로 완료한다.

11. 침 치료의 예측 이상반응 및 사용상의 주의사항

가. 예측 이상반응

체침치료 및 전침치료는 일반적으로 발생할 가능성이 있는 내용을 근거로 하여 발적, 종창, 부종, 통증, 자반 등 이상반응이 발생할 수 있을 것으로 사료됨.

나. 사용상의 주의사항

체침 치료 및 전침 치료시 주의사항은 다음과 같다
침치료 보관시 위생에 주의할 것
자침시 위생 및 감염에 주의할 것
환자가 심한 통증을 호소시 중단할 것

12. 임상시험 중지, 연구대상자 탈락기준

다음 조건의 하나라도 해당될 때 담당주치의의 판단에 의해 시험에서 제외한다.

가. 시험종료일 기준으로 예측되는 치료 순응도가 70%미만인 경우 연구대상자는 탈락하는 것을 원칙으로 한다. 방문 지정일로부터 ±3일 이내에 방문하지 않으면 방문하지 않은 것으로 간주한다.
나. 연구기간 중 임신했을 때
다. 사고 등의 재해나 타 질환의 발병으로 수술 또는 입원치료가 필요한 경우
라. 임상시험연구대상자가 거부한 경우
마. 금지 병용 약물 및 요법을 실시했을 때
예) 항응고제, 향정신성 약물, 항우울 약물, 갑상선기능항진증 관련 약물, 건강기능식품 (홍삼), OTC 중 혈류개선제, 그 외에 수부 족부냉증에 영향을 줄 가능성이 있는 약물 등.
바. 수부 또는 족부냉증 증상의 악화로 표준 치료가 필요한 경우.
사. 중대한 이상반응으로 임상시험을 지속하기 곤란하다고 판단되는 경우
아. 기타 연구를 지속하기 힘든 부득이한 사유가 발생했거나 임상시험 결과에 영향을 끼쳤을 것으로 임상시험책임자가 판단한 경우

탈락한 연구대상자에 대해서는 연구대상자 본인이 추가 치료를 원할 경우, 수족냉증의 기존 한의학적 치료를 권장할 예정이며, 유지치료를 원하는 경우에도 표준화된 치료를 권장할 예정이다. 이러한 탈락한 연구대상자에 대한 치료기간, 치료의 유/무상의 보상 문제 등은 탈락한 연구대상자의 상태, 침치료와의 인과관계에 따라 별도의 보상기준을 임상시험위원회 따른다.

13. 안전성의 평가기준, 평가방법 및 보고방법

가. 예측 가능한 또는 불가능 이상반응

이상반응이란 임상시험 진행 과정에서 발생한 바람직하지 않거나, 의도되지 않은 증후, 증상 또는 질병을 가진 사례를 말하며, 중재와 반드시 인과관계를 가져야 하는 것은 아니다.

예측 가능한 이상반응
체침 또는 전침 시술 관련하여 발생 가능한 이상반응의 종류는 다음과 같다.
- 국소적 이상
  - 피하출혈 및 출혈반 형성
  - 말초신경염/ 봉와직염
  - 국소 부위 통전감

자침 부위 경련 혹은 화상
침에 대한 알레르기 반응
자침 부위의 통증(2주 이상 지속되는 경우에 국한)

- 자율신경계 및 중추신경계 이상

혈압 강하 및 실신/ 과도한 발한
졸린 증세(1주 이상 지속되는 경우에 국한)
혼미(1일 이상 지속되는 경우에 국한)/ 혼수

- 위장관 이상

오심/ 구토

- 정신적 문제

불안, 공포(사건 당 60시간 이상 지속되는 경우에 국한)
기면

- 기타

두통(3일 이상 지속되는 경우에 국한)
저린 증상을 동반한 과민감각(3일 이상 지속되는 경우에 국한)
자침 후의 발작
불명료 언어

예측 불가능한 이상반응

이용 가능한 체침 및 전침 시술 관련 정보(침 시술 지침서, 침구의학 교과서, 관련 논문 등)에 비추어 양상이나 위해의 정도에서 차이가 나는 것을 말한다.

사용상의 주의사항

체침 또는 전침 치료가 예정된 당일 대상자가 지나친 피로감 혹은 건강상태 저하를 호소하거나, 시험 담당 한의사가 사진(四診)을 통해 대상자를 검진한 결과, 대상자의 상태가 전침 치료에 적합하지 않다고 판단될 경우 해당일 3일 전후 이내에서 치료 일자를 조정함으로써, 전침 치료와 연관된 잠재적 이상사례를 피할 수 있다.

나. 안전성 평가 대상

안전성 평가는 무작위배정을 받고 임상시험에 참여한 모든 대상자를 포함한다.

다. 안전성 평가

- 혈액검사 : 치료 전후 혈액검사를 시행하여 이상반응을 확인한다.
- 심혈관계 : 매 방문시 혈압 및 맥박수의 측정한다.

라. 이상반응 관찰

임상시험 기간 중 발생한 모든 이상사례에 대한 정보를 대상자가 자발적으로 보고하도록 교육하며, 시험 담당자는 임상실험실검사 상 수치 및 문진 등을 통하여 이상사례 발현 여부를 확인한다.

임상시험담당자는 이상반응을 평가하고 이전에 기록된 적이 없는 이상반응은 증례기록서의 해

당란에 기록하되 조치사항, 해당 이상반응의 성질, 발현일자 및 시간, 지속시간, 중중도 및 체침 또는 전침과의 관련성 등을 밝혀야 한다. 시험기간 중에 이상반응의 빈도나 중증도에 변화가 있을 경우에는 별도의 페이지에 기록한다.

1) 이상반응의 중증도 평가

이상반응의 임상적인 증상 및 징후에 따른 판정기준은 다음과 같다.

1 = 경증(Mild)	연구대상자가 거의 느끼지 못할 정도로 정상적인 일상생활(기능)을 저해치 않는 정도. 대부분 치료가 필요하지 않는 정도
2 = 중등도(Moderate)	연구대상자가 불편감을 느낄 수 있으며, 정상적인 일상생활(기능)을 저해하는 정도. 연구대상자가 시험을 계속할 수는 있으나 치료가 필요할 수도 있는 정도
3 = 중증(Sever)	연구대상자가 매우 불편하여 일상생활(기능)이 불가능하고, 시험의 계속적인 참여가 불가능한 정도, 치료나 입원이 필요할 수 있는 정도

2) 침치료와 인과관계 평가

이상반응이 체침 또는 전침치료 (또는 기초질환의 자연병력, 병용요법 등 다른 원인)에 어느 정도 기인하는지의 확실성 정도는 이상반응이 다음에 얼마나 부합되는가에 의해 결정되며 이상반응이 침 치료를 시작한 후 나타났다가 사용중단 후 소실되었거나 재사용 시 다시 나타남으로써 침치료와 관련 있음을 반증한다.

1 = 명확히 관련이 있음 Certain >90%
2 = 상당히 관련이 있음 Probable/likely >70%
3 = 관련이 있을 가능성이 있음 Possible 50%
4 = 관련이 없을 것으로 생각됨 Unlikely <30%
5 = 관련성 여부를 확인하기 어려움 Conditional/ unclassified <10%

3) 중대한 이상반응

"중대한 이상반응"이라 함은 다음 각목의 1에 해당하는 경우를 말한다.

가) 사망을 초래하거나 생명을 위협하는 경우
나) 입원 또는 입원기간의 연장이 필요한 경우
다) 지속적 또는 의미 있는 불구나 기능 저하를 초래하는 경우
라) 선천적 기형 또는 이상을 초래하는 경우

또한 임상시험담당자가 심각하다고 판단한 경우나 체침 또는 전침치료와 관련하여 중대한 위험, 금기사항, 부작용 또는 주의사항을 암시하는 경우에는 심각한 사례로 보고해야 한다. 임상시험담당자는 이상반응이 나타난 환자의 경우 해당 이상반응이 사라지거나 안정된 상태에 이를 때까지 추적관찰 해야 한다.

4) 이상반응과 관련하여 취해진 조치

0 = 취해진 조치 없음

1 = 일시적 중단
2 = 체침 또는 전침 중재 중단
3 = 약물 치료
4 = 비약물 치료
5 = 입원/입원 기간의 연장

마. 보고

시험책임자는 공동연구자 및 대상자에게 체침 또는 전침 중재 후 나타날 수 있는 모든 이상반응에 대하여 교육을 실시하고 중재 후 나타나는 모든 현상에 대하여 보고하도록 교육을 실시한다.

체침 또는 전침 중재 후 전신적 또는 임상병리학적으로 나타나는 제반증상에 대하여 종류, 발생시간, 정도, 처치, 치료약제, 경과, 침치료와의 인과관계 등에 대한 기록 및 보관은 임상시험 관리기준에 준하도록 증례기록표에 기입한다.

시험기간 중 "중대한 이상반응(serious adverse events)" 발생시에는 임상시험심사 위원회(IRB)에 보고하여 시험의 지속 또는 중단 여부를 결정한다.

1) 연구대상자가 시험기간 중 사망하였거나, 심각한 생명의 위험을 가져온 경우에는 이 사실을 보고 받은 일로부터 7일 이내, 다만, 이 경우 상세한 정보를 최초 보고일로부터 8일 이내에 추가로 보고한다.
2) 기타 다른 모든 중대하고 예상하지 못한 이상반응의 경우에는 의뢰자가 이 사실을 보고 받거나 알게 된 날로부터 15일 이내에 보고한다.
3) 위의 사항들에 대한 보고와 관련하여 추가적인 안전성 정보는 임상시험 종료 후 7일(washout period)까지 연구자의 전화 또는 외래방문으로 보고하도록 한다.
4) 시험책임자는 연구를 시행함에 있어서 모든 제반 사항을 Declaration of Helsinki에 준하여 시행한다.

□ 가임기 여성
① 가임기 여성은 임상연구 참여 이전에 소변 임신검사에서 음성반응을 보여야 한다.
② 임신할 가능성이 있는 여성은 적절한 피임법으로 연구 시작 최소 1개월 전 이상 확실한 피임을 하고 있어야 하며, 연구 기간 중에도 지속되어야 한다.
③ 임상연구 기간 동안 임신을 한 것으로 생각된다면 즉시 연구 담당자에게 알리며, 임신검사가 반복될 수 있다.

바. 이상반응 발생 시 조치사항

본 시험기간 중 임상시험 연구책임자 및 담당자는 대상자의 안전에 만전을 기하여야 하며, 중대한 이상반응 발생 시에는 시험을 중지하고 신속하고 적절한 조치를 취하여 이상반응을 최소화하여야 한다.

임상시험 중 "중대한 이상반응" 발생 시 각 담당자의 의무는 다음과 같다.

1) 임상시험책임자의 의무

임상시험책임자는 임상시험 중 중대한 이상반응이 발생한 때에는 즉시 기관윤리심의위원회 및 임상시험 의뢰자에게 보고하고 별도의 지시가 있을 때까지 해당 체침 및 전침치료에 대한 임상시험의 일부 또는 전부를 중지하여야 한다.

2) 임상시험담당자의 의무

임상시험담당자는 임상시험 실시 중에 중대한 이상반응 등이 발생한 경우에는 즉시 임상시험책임자 및 임상시험의뢰자에게 보고하여야 한다.

3) 기관윤리심의위원회의 의무

기관윤리심의위원회는 중대한 이상반응이 나타난 경우에는 임상시험의 일부 또는 전부에 대하여 중지명령 등 필요한 조치를 임상시험책임자에게 하여야 한다.

14. 유효성 평가방법 및 해석방법

가. 일차평가지표 Primary outcome variable

1) 수부 및 족부의 시각적 상사척도(Visual Analogue Scale : VAS) :

○ 매 방문 시 10cm길이의 선위에 대상자가 자신의 냉증 정도에 해당하는 숫자를 주어진 숫자 중에 선택하도록 한다. 수부의 시각적 상사척도는 직선의 왼쪽 끝에 '수부냉증 없음', 오른쪽 끝에는 '가장 심한 수부냉증'이라는 설명을 추가하고, 족부의 시각적 상사척도는 직선의 왼쪽 끝에 '족부냉증 없음', 오른쪽 끝에는 '가장 심한 족부냉증'이라는 설명을 추가한다. 점수는 대상자가 'I' 표시한 위치를 자로 잰 숫자를 점수로 삼는다.

○ 매 방문시 평가한 VAS 결과의 치료시작 전과 치료 종료 후의 결과 차이를 각 내원일별 살펴봄

[참고문헌]

류갑순 외. 급만성 허혈로 유발된 냉증 치험례. 대한한방부인과학회지. 2009;22(2):222-30.

이미주 외. 청리자감탕으로 호전된 갱년기 여성의 상열감과 복부냉증에 대한 DITI를 이용한 평가. 대한한방체열의학회. 2010;8(1):26-32.

조준영 외. 수족냉증 환자 치험 1례. 대한한방부인과학회지. 2011;24(3):195-202.

이윤재, 이경섭. 냉부하검사로 살펴본 원적외선 기능성 제품이 수부 족부냉증에 미치는 영향 A Pilot Study. 2008;6(1):69-75.

■ 시각적 상사척도 (Visual Analogue Scale : VAS)

최근 1주일 동안 하루 평균 수부 냉증의 정도를 아래의 그래프에 'I'자로 표시해주십시오.

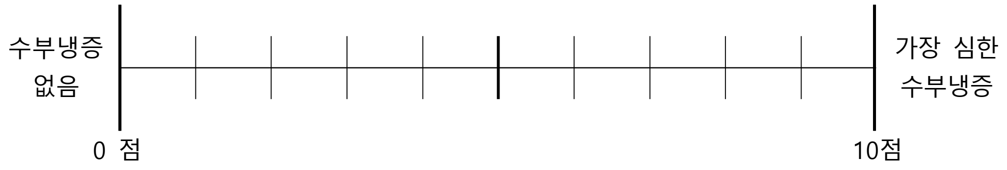

점수 :

■ 시각적 상사척도 (Visual Analogue Scale : VAS)

최근 1주일 동안 하루 평균 족부 냉증의 정도를 아래의 그래프에 'I'자로 표시해주십시오.

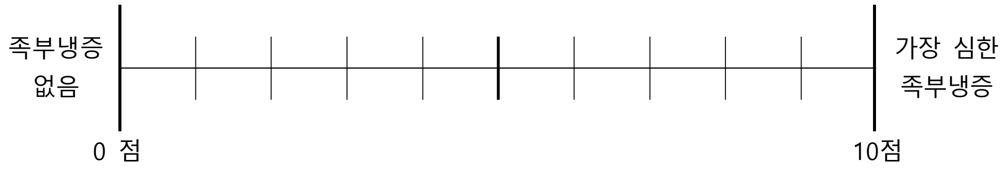

점수 :

나. 이차평가지표 Secondary outcome variable

1) Body temperature : 써모미터(Thermometer) 측정

○ 써모미터(Thermometer)로 치료시작 전과 치료 종료 후 각각 10분 이내에 측정한 양측 수장부위(PC8)와 전상완 중심부(LU4), 전대퇴 중심부(ST32)와 족배부 전면부(LR3)의 온도 차이를 각 내원별로 살펴봄

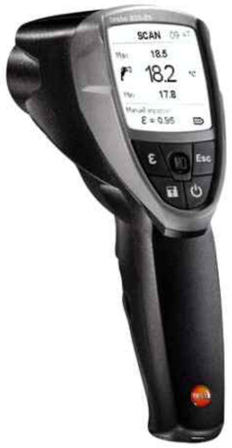

	testo 835-T1	testo 835-T2	testo 835-H1
적외선			
초점비	50:1(측정 대상과의 거리가 표준 2.0m 일 때) + 센서의 개구부 지름(24mm)		
측정점	4 포인트 레이저		
광학 범위	8 ~ 14 µm		
측정 범위	-30 ~ +600 ℃	-10 ~ +1500 ℃	-30 ~ +600 ℃
정확도<br>±1 digit	±2.5 ℃(-30 ~ -20.1 ℃)<br>±1.5 ℃(-20 ~ -0.1 ℃)<br>±1 ℃(+0 ~ +99.9 ℃)<br>측정값의 ±1%(나머지 범위)	±2 ℃ 또는 측정값의 ±1%	±2.5 ℃(-30 ~ -20.1 ℃)<br>±1.5 ℃(-20 ~ -0.1 ℃)<br>±1 ℃(+0 ~ +99.9 ℃)<br>측정값의 ±1%(나머지 범위)
분해능	0.1 ℃	0.1 ℃ (-10 ~ +999.9 ℃)<br>1 ℃ (+1000 ~ +1500 ℃)	0.1 ℃
K타입(NiCr-Ni)			
측정범위	-50 ~ +600 ℃	-50 ~ +1000 ℃	-50 ~ +600 ℃
정확도<br>±1 digit	±(-0.5 ℃ 측정값의 +0.5%)		
분해능	0.1 ℃		
공통 기술데이터			
방사율	0.10 ~ 1.00 (0.01씩 범위조절 가능)		
방사율값 저장개수	20개 저장 가능		
레이저 측정점	On / Off		
메모리	200개 저장 가능		
알람(한계값)	IR 온도, TC 온도		
알람 신호	시각, 청각		
작동온도	-20 ~ +50 ℃		
보관온도	-30 ~ +50 ℃		
재질/하우징	ABS + PC		
크기	193 x 166 x 63 mm		
무게	514 g		
배터리 타입	AA타입 배터리 3개(또는 PC에서 USB로 충전 가능)		
배터리 수명	25시간(25 ℃에서 레이저 및 백라이트 없을 시)<br>10시간(25 ℃에서 백라이트 없을 시)		
디스플레이	도트 매트릭스		
자동꺼짐<br>(측정중/USB연결중일 때)	백라이트: 30초<br>측정기: 120초		
표준	EN 61326-1:2006		
보증기간	2년		

[참고문헌]

이미주 외. 청리자감탕으로 호전된 갱년기 여성의 상열감과 복부냉증에 대한 DITI를 이용한 평가. 대한한방체열의학회. 2010;8(1):26-32.

김은경 외. 적외선 체열검사로 복부냉증의 진단과 치료 경과를 평가한 단일 임상례. 대한한방체열의학회지. 2009:7(1):55-65.

조준영 외. 수족냉증 환자 치험 1례. 대한한방부인과학회지. 2011;24(3):195-202.

이윤재, 이경섭. 냉부하검사로 살펴본 원적외선 기능성 기능성 제품이 족부냉증에 미치는 영향 A Pilot Study. 2008;6(1):69-75.

2) 삶의 질 평가 설문지(WHOQOL-BREF)과 삶의 질 평가 EQ-5D

○ 전반적인 삶의 질, 전반적인 건강상태, 신체적 건강, 심리적 건강, 사회적 관계, 환경 등 각 항목에 대한 검사를 방문1, 방문10, 방문11에 측정

3) 변증 설문지

○ 변증 설문지는 변증지표에 따른 수족냉증 환자의 증상 및 VAS 변화와의 상관관계를 분석하기 위해 방문 방문1에 측정

4) 냉증 진단 설문지

○ 냉증 진단 설문지를 통하여, 환자의 냉증에 대한 주관적 또는 객관적 증상에 증상을 설문검사하며, 방문1, 방문10, 방문11에 측정

15. 통계분석방법

가. 일반적인 원칙

기본적인 분석방법은 유효성평가지표들에 대하여 임상시험 전에 시행한 기초검사 결과와 임상시험 종료 시 방문하였을 때 시행한 검사결과간의 변화 정도이며 유의수준은 양측검정으로 0.05으로 한다.

이 시험의 유효성 평가 부분을 위해서는 임상시험의 연구대상자들로부터 얻어진 모든 자료는 선정/제외 기준에 적합하였고, 인체시험 참여에 자발적으로 동의한 모든 연구대상자 중 임상시험계획서의 위반이나 중도 탈락과는 상관없이 무작위배정을 받은 모든 연구대상자를 분석에 포함시키는 방법인 배정된 대로 분석(intention-to-treat analysis; ITT)과 불순응 또는 결측자료가 있는 연구대상자를 제외한 치료받은 대로 분석(per-protocol analysis: PP)을 모두 시행한다. 유효성 평가 변수의 결측치의 경우 LOCF(Last Observation Carried Forwar)방법을 사용하여 분석하고, 안전성 평가 변수의 경우 결측치는 결측으로 처리하여 분석을 시행한다.

배정된 대로 분석에는 기초검사를 받고 최종 검사까지 완료한 연구대상자를 모두 포함시키며, 이때 마지막 검사자료를 최종검사자료로 간주한다. 안전성 분석 방법도 배정된 대로 분석방법에 준한다. 치료받은 대로 분석에는 커다란 계획서의 위반내용이 없이 최종검사까지 수행한 환자가 분석대상이 된다.

두 가지 분석의 결과는 동일 임상시험 내에서는 이론적으로 일치하여야 하나 분석결과 간에 유의한 차이가 발생하는 경우에는 그러한 차이에 대한 타당한 설명이 제시되어야 한다.

본 임상시험계획서가 정하는 이외의 통계분석에 관한 사항은 임상시험통계지침을 참고한다.

나. 기저선의 인구학적 및 평가 변수 비교

치료군과 대조군의 변수 분포가 동질한지 검정하기 위하여 연속형 변수에 대해 분산분석(ANOVA) 또는 비모수 방법을 이용하며, 범주형 변수에 대하여 Chi-square test 등을 실시한다.

다. 유효성 변수의 분석방법

1) 1차 유효성 평가 변수 : 수부 및 족부냉증의 시각적 상사척도 (Visual Analogue Scale ; VAS)

일차 유효성 평가 변수 분석은 ITT군을 주분석군으로 하며, 추가적으로 PP군 분석결과를 제시한다. 무처치대조군과 침치료군 또는 전침치료군간 VAS 점수 변화에 차이가 있는 지를 검정하기 위하여 repeated ANOVA, paired t-test 등을 실시한다. 각 군 내의 VAS 점수의 개선 정도의 비교는 치료 전후의 VAS 점수의 차이 (Weeks 5±3days 시의 VAS 점수 – 방문 1의 VAS 점수) 를 이용하여 paired t-test를 실시한다.

2) 2차 유효성 평가 변수

(1) Body Temperature(BT; 써모미터 측정) : 침치료군과 전침치료군간 BT 변화에 차이가 있는 지를 검정하기 위하여 repeated ANOVA 등을 실시한다. 각 군 내의 BT의 개선 정도의 비교는 침치료 전후의 BT의 차이 (Weeks 5±3days 시의 BT – 방문 1의 BT) 를 이용하여 paired t-test를 실시한다.

(2) WHOQOL-BREF 삶의 질 평가 설문지 : 각 군의 차이를 검정하기 위해 ANOVA 등과 사후검정을 이용하여 분석한다. 각 군 내의 비교는 침치료 전후의 값 차이 (Weeks 5±3days 시의 값 – 방문 1의 값)를 이용하여 paired t-test 등을 실시한다.

(3) 변증설문지:

○ 변증을 위한 관찰대상(변증지표)가 수족냉증을 가진 대상자들에서 어떤 분포를 보이는 지 도시한다. 변증지표와 증상 VAS의 변화사이의 관계를 파악하기 위하여 각 지표마다 증상VAS의 변화량을 종속변수로 하여 ANOVA test(정규분포를 따르지 않는 경우 비모수검사) 및 사후검정을 시행한다.

○ 기본변증도구의 타당도와 신뢰도 평가를 실시함. 타당도 평가는 a. 구인타당도평가(construct validity) : 기본변증도구 문항들의 요인분석을 시행하여 구인타당도를 평가함. 기본변증도구의 각 문항점수를 얻은 후, 문항들 간의 상관계수 행렬을 구함. 요인 고유값(eigen value)에 의하여 요인 수를 결정하고, 구인들을 확인한 후 요인을 회전시키고, 회전된 요인과 관계있는 요인 부하량이 큰 문항들의 문항내용에 근거하여 해석하고 이름을 부여함. b. 예측타당도(predictive validity) : 수족냉증 시각상사척도 값과 수족냉증을 설명하는 요인의 점수의 상관관계를 평가함. 수족냉증 환자의 시각상사척도 값과 구인타당도 평가결과 수족냉증을 잘 설명할 수 있는 문항의 점수 합과의 상관 정도를 추정함. 설문지의 평가를 위해 크롬바흐 알파를 측정한다. 이 외에 추가적으로, 치료 전후 해당 변증 항목의 변화와 각 항목의 정도의 변화, 특정한 변증 유형별 치료 효과의 크기 등을 분석 검토한다. 또한, 수족냉증에 관련된 변증항목과의 연관성과 변증 관련도를 파악하기 위해 군집분석 또는 요인분석을 활용하여 검토한다.

(4) 냉증 진단 설문지:

○ 신뢰도 분석을 위해 크롬바흐 알파 분석을 실시한다. 또한, Item-Total Correlation의 통계 방법을 이용하여, 한 항목(item)이 잔존하는 나머지 항목의 전체 점수간의 피어슨 상관계수를 측정하여, 항목의 상관성을 분석한다.

○ 군집분석(cluster analysis)과 요인분석(factor analysis)를 추가적으로 실시한다.

(5) 삶의 질 평가 EQ-5D : EQ-5D index score를 통해 건강과 관련된 삶의 질에 지장이 있다고 평가할 수 있는 cutoff value가 공식적으로 설정되어 있지 않지만, 4분위, 5분위수를 이용하여 경제성 평가시 EQ-5D index score를 활용한다.

라. 안전성 평가 변수에 대한 분석

1) 임상실험실 검사 자료에 대해서는 변수의 특성에 따라 치료 전, 후의 군내 비교 등 적절한 통계적 방법을 이용하여 분석하고, 이상반응의 빈도, 발현율, 각각의 목록, 상세한 발현시간, 심각한 정도 및 침치료와의 인과관계 등을 제시하며, 필요한 경우 그래프 형태로 보고한다. 이에 대한 통계적 분석은 변수의 특성에 따라 ANOVA, t-test, chi-square 혹은 Fisher's exact test 등을 실시한다.
2) 자 · 타각 증상 등 이상반응: 발현된 모든 이상반응은 각 치료군별 및 신체기관별로 분류하여 자세한 설명과 함께 table 화 한다. 각 군 별로 침치료와의 인과관계가 있는 이상반응과 인과관계가 없는 이상반응의 빈도를 기록하다. 이상반응 발현건수와 1 회 이상의 이상반응을 경험한 연구대상자의 비율에 대하여 군내에서 95% 신뢰구간을 구하고 군간 비교를 실시한다.
3) 임상병리검사 : 혈액학 및 혈액생화학 검사 결과, 활력징후(vital signs) 등과 같은 연속형 자료에 대해서는 각 군별 및 방문별로 기술통계량을 제시하며 방문 시점간의 차이에 대하여 paired t-test 또는 비모수 방법을 이용하여 분석한다.

마. 결측치 분석

1) 통계분석에서 유효성 평가 변수 중, 계획된 시험에서 얻을 수 있는 관측값 중에 어떠한 이유로 관측되지 않은 측정값, 즉 결측값(missing value)인 경우에는 이전 방분의 측정값으로 대체하는 LOCF(Last Observation Carried Forwar)방법을 사용하여 분석한다. 안전성 평가 변수의 경우 결측치는 결측으로 처리하여 분석한다.

16. 임상시험의 순응도와 프로토콜 위반에 대한 처리

가. 본 연구의 연구책임자와 담당자는 임상시험 계획서의 위반이 발생하지 않도록 계획서에 대해 충분히 숙지하고 철저히 이행하여야 한다. 반면, 부득이하게 발생된 계획서 위반 사항에 대해서는 다음에 따라 처리한다.

나. 심각한 위반의 경우, 예컨대, 대상자 선정/제외기준 위반, 동의서 미취득 등 연구대상자의 안전과 시험결과에 중대한 영향을 미칠 수 있는 위반의 경우 해당 대상자를 연구에서 탈락 처리(drop-out)함을 원칙으로 한다.

다. 기타 경미한 위반 사항은 위반 또는 지연 정도와 사유를 정확히 기재하고 결과 분석 시 시험연구에 영향을 주었는지 고찰한다.

17. 자료관리

임상시험 총 책임자기관은 본 임상시험의 자료관리를 객관성, 안전성, 보완성을 유지할 수 있는 방법으로 아래의 절차에 따라 시행한다. 본 임상시험이 종료되면 본 임상시험만의 자료를 별도 파일로 3년간 보관한다.

가. 증례기록서 기록 및 원시자료 확인

증례기록지 (CRF)에는 기록해야 할 자료가 발생할 시 즉시 기록한다. 만약 증례 종료 시까지 기록되지 않은 경우 적절한 누락사유를 기록하여야 한다. 모든 수정사항은 먼저의 기록이 보이도록 한 줄로 그어 표시한 후 수정자료, 수정자, 수정사유, 수정일을 기록한다. 먼저의 기록이 보이지 않도록 하는 수정액 등을 사용하여서는 안 된다.

증례기록이 완료된 연구대상자의 CRF는 별도의 모니터 요원이 속해 있는 단체의 SOP에 정해진 대로 Source Document Verification 과정을 거쳐 전산시스템에 입력할 수 있도록 연구대상자 별로 경주관 임상시험 실시 기관 전산입력자에게 전달된다. 모든 자료가 전산 입력된 후에도 관련 정부기관, 의뢰자 등의 요구에 의해 확인될 수 있도록 원시자료 (Source document) 및 증례기록지 (CRF)를 보관한다.

연구자는 임상시험이 시작되기 전 CRF 등 적절한 위치에 정상범위 및 참고치를 제시하여 전산기록 (computerized record)을 확인 (Verification)이나 검증 (Validation)에 사용할 수 있도록 한다.

18. 임상시험참가자 설명문 및 동의서

본 시험의 실시에 있어서 연구대상자에게 본 시험의 내용 및 전침치료의 효과, 이상반응에 대해 사전에 충분히 설명한 후 환자의 동의를 얻어 동의서를 작성하고 증례기록서에 동의 취득연월일을 기재한다.

19. 피해자 보상에 대한 규약

별첨

20. 임상시험 후 연구대상자의 진료 및 치료기준(방안)

처치를 요하는 이상반응이 발생한 경우 혹은 지속적인 치료를 요하는 연구대상자의 경우에는 전문의의 진단 및 적절한 처치를 받도록 한다.

21. 연구대상자의 안전 보호에 관한 대책 및 기타

가. 치료 전 검사를 통하여 연구대상자가 본 임상시험에 적절한 지를 엄격히 평가한다.

나. 임상시험과 관련된 것으로 보이는 이상반응에 대해 임상시험책임자는 이를 즉시 기관윤리심의위원회에 알리고 지침을 따른다. 단, 응급처치를 요하는 경우는 시험책임자의 책임 하에 선처

치 후 보고를 할 수 있다.

다. 신체적 손상(injury)의 위험성을 수반(발진, 감염 등과 같이 신체에 영향을 주는 부작용은 모두 포함)할 수 있는 모든 가능성을 고려한다.

라. 연구대상자가 연구에 참여한 것의 직접적인 결과로 발생한 (통상적 진료에서 보통 예상되는 것이 아닌) 신체적 손상에 대한 치료를 받을 경우 연구대상자는 진료비를 부담하지 않는다.

마. 상지대학교 산학협력단이 의뢰한 연구에서 발생한 신체적 손상에 대한 치료비는 연구자의 고의 또는 부주의로 인한 중대한 과실이 아니면 본 연구를 위해 가입한 보험회사에서 부담한다.

바. 임상시험 도중 수족냉증 악화시 수족냉증에 대한 한의학적 치료를 실시한다. 수족냉증에 대한 표준화된 치료의 방법은 없으므로, 한의학에서 기존적으로 시행하고 있는 치료방법을 시행할 예정이다.

22. 기타 임상시험을 안전하고 과학적으로 실시하기 위하여 필요한 사항

가. 임상시험관리기준(KGCP)

본 임상시험을 실시함에 있어 KGCP 및 Helsinki 선언의 근본정신을 준수하여 윤리적이고 과학적인 배려 하에 연구를 실시하도록 한다. 본 임상시험계획서에서 제시하지 않는 내용은 KGCP와 식약청고시 및 Helsinki 선언의 내용을 따른다.

나. 연구대상자 동의

임상시험담당자는 임상시험에 참여하는 연구대상자에게 사전에 시험의 성격, 범위, 예상되는 결과 등에 대해 쉽게 이해할 수 있도록 설명하며 동의서에는 임상시험담당자 및 연구대상자가 서명한다.

다. 비밀보장

모든 연구대상자명은 비밀을 유지해야 한다. 서명을 받은 연구대상자 동의서는 시험책임자가 보관한다. 시험책임자는 연구대상자번호 및 연구대상자명이 기록된 리스트를 갖추어 놓아 나중에 기록을 찾을 수 있도록 한다.

라. 임상시험 모니터

1) 연구대상자의 권리와 복지보호, 보고된 임상시험 관련 자료가 근거문서와 대조하여 정확하고, 완전하며, 검증이 가능한지 여부 확인, 임상시험이 승인된 계획서, 임상시험관리기준 및 시행규칙의 규정에 따라 수행되는지의 여부 확인을 위하여 모니터링을 실시한다.

2) 임상시험 모니터요원은 필요한 경우에 전화, 전자우편 또는 직접방문 등의 방법으로 본 시험에 대한 모니터링을 실시한다. 임상시험 모니터요원과 시험자들은 모든 시험의 진행상황에 대해 검토하고 증례기록서의 모든 기록들을 확인한다. 또한 시험계획서에 따라 임상시험이 진행되는지에 대해 확인하고 발생하는 모든 문제점에 대해 연구자와 의견을 교환하고 조정한다.

3) 방문 시간은 시험책임자와 임상시험 모니터요원과 협의하여 정하여야 한다.

4) 시험책임자는 또한 임상시험관리기준에 정의된 것과 같이, 모니터가 증례기록서에 기입된 자료들을 확인할 수 있는 연구대상자의 원 자료들(source documents : 병원 또는 개인차트, 실험실 결과 기록, 예약 기록 등)을 볼 수 있도록 해주어야 한다.

23. 참고문헌

1) 이수림, 이경섭, 송병기. 부인냉증에 관한 문헌적 고찰. 대한한방부인과학회지 9(1):55-80, 1996
2) 현대의 한방치료. 한성출판사. 1989
3) 부인과 환자의 냉증과의 관계에 대한 조사 연구 대한한방부인과학회지 15(2); 2002
4) 조기호. 동서의학 진료 가이드 북. 서울: 고려의학; 2001, p. 284-6.
5) 이남헌 외. EBM 기반 구축을 위한 온경탕 연구논문 분석. 대한한의학방제학회지. 2008;16(2):1-9.
6) 조수원 외. 온경탕 보험 엑스산제의 품질평가 연구. 대한침구학회지. 2010;27(2):105-113
7) 김철민 외. 족부냉증과 레이노병에 대한 일부 가정의의 인식도. 대한가정의학회지. 2007;28:339-45
8) 박주영 외. 중풍환자에서 족부냉증과 적혈구변형능의 관련성 연구. 대한한방내과학회지. 2010;31(3):578-85
9) 한지영 외. 냉부하검사를 통한 수부냉증의 진단. 2003;2(1):17-23
10) 이경섭. 냉증 진단을 위한 DITI와 말초신경검사의 임상적 평가.한방체열의학회지. 2004;3(1):60-66
11) 김동환 외. DITI를 이용한 수족 냉증 진단의 표준화. 대한한방부인과학회지. 2001;14(2): 120-134
12) 이미주 외. 강남경희한방병원에 내원한 냉증을 호소하는 여성의 Heart Rate Variability 특성 연구. 대한한방부인과학회지.2011;24(3):109-115.
13) 윤성우 외. 적외선 체열촬영을 이용한 배변습관과 하복 및 수족 냉증의 관련성 고찰. 한방체열의학회지. 2004;3(1):36-42
14) 박경선 외. 산후풍 환자의 적외선 체열 영상 특성에 대한 연구. 대한한방부인과학회지. 2010;23(2):116-123
15) 김혜원 외. DITI를 통한 불임여성의 체열에 대한 연구. 대한한방체열의학회지. 2002;1(1):52-56
16) Park KS, Park KI, Kim JW, Yun YJ, Kim SH, Lee CH, Park JW, Lee MJ. Efficacy and safety of Korean red ginseng for cold hypersensitivity in the hands and feet : A randomized, double-blind, placebo-controlled trial. Journal of Ethnopharmacology 2014;158:25–32
17) 류갑순외. 급만성 허혈로 유발된 냉증 치험례. 대한한방부인과학회지. 2009;22(2):222-30.
18) 이미주 외. 청리자감탕으로 호전된 갱년기 여성의 상열감과 복부냉증에 대한 DITI를 이용한 평가. 대한한방체열의학회. 2010;8(1):26-32.
19) 조준영 외. 수족냉증 환자 치험 1례. 대한한방부인과학회지. 2011;24(3):195-202.
20) 하헌용 외. 냉증에 대한 산소챔버의 임상 효능 및 안전성 연구. 대한한방부인과학회지. 2013;26(4):123-139.
21) 이윤재, 이경섭. 냉부하검사로 살펴본 원적외선 기능성 제품이 족부냉증에 미치는 영향 A Pilot Study. 2008;6(1):69-75.
22) Nishida S, Eguchi E, Ohira T, Kitamura A, Kato YH, Hagihara K, Iso H. Effects of a traditional herbal medicine on peripheral blood flow in women experiencing peripheral coldness: a randomized controlled trial. BMC Complement Altern Med. 2015 Apr 2;15:105. doi: 10.1186/s12906-015-0617-4.
23) 张欣, 李永凯. 温针灸治疗末梢神经炎之感觉障碍临床观察. 新疆中医药2007 年第25 卷第6 期(总第 112 期
24) 전상우 외. 레이노병의 침치료에 대한 연구동향 대한한방내과학회지 2017;38(4):433-442

[별첨 1] 피해자 보상에 대한 규약

[별첨1] 피해자 보상에 대한 규약

피해자 보상에 대한 규약

1) 임상시험 연구제목 : **수족냉증에 대한 전침 치료의 유효성 안전성에 대한 다기관 임상연구 - 무작위배정, 대조군 임상시험 -**

2) 원칙

① 본 임상시험과 협약된 보험회사는 연구대상자의 신체적인 손상(사망포함)에 대해 보상한다.

② 손상의 원인이 임상연구의 침치료의 실시로 인해 발생하였을 때 본 임상시험과 협약된 보험회사는 임상연구 대상자에게 보상한다.

③ 일시적 통증 또는 쉽게 치료될 수 있는 정도의 손상이 아니라 지속적이고 불구가 될 수 있을 정도의 심각한 손상에 대해서만 보상한다.

④ 임상연구의 침치료에 의해 생긴 부작용이나 부작용처치 과정에서 발생된 손상이 있는 경우, 임상연구용 침치료가 직접적인 원인이 된 손상에 대하여 보상한다.

⑤ 본 임상연구로 인하여 대상자가 위해를 입은 경우 임상시험 책임자가 가입한 보험을 통하여 이를 보상한다.

3) 다음의 경우에는 보상하지 아니한다.

① 본 임상시험 후원 하에 집행되지 않았거나 제공되지 않은 처치나 의약품으로 발생한 부작용에 의한 손상

② 임상연구용 침치료의 적응증에 대한 효과 또는 혜택을 제공하지 못함에 대한 보상

③ 서로 합의한 시험계획서에 순응하지 않음으로써 야기된 손상

④ 연구 대상자 또는 보호자의 부주의에 의해서 초래된 손상

4) 보상평가기준

① 보상수준은 손상의 본질, 그 정도, 지속성 여부 등에 적절한 액수여야 하며, 한국의 법정에 의해 유사손상들에 대해 일반적으로 지급되는 것과 동일해야 한다.

② 보상수준에 대해서 연구대상자와 연구책임자 사이에 이견이 있을 경우, 양자가 수용할 수 있는 전문가의 자문을 구하여야 한다.

본 임상시험의 총책임자인 상지대학교 부속한방병원 유준상은 본 임상시험을 위해 임상보상보험에 가입하였으며, 앞에서 언급한 여러 제반내용을 참고하여, 연구 대상자가 본 시험에 의해 어떠한 불이익이라도 받지 않도록 주의하며, 만약 본 시험에 의해 문제점이 발생한 경우 피해자 보상규약에 의거하여 책임을 질 것을 서약합니다.

날 짜 : 2018년 12 월 30 일

연구 총책임자 소속 : 상지대학교 부속한방병원

서 명 : 유 준 상 교수 (인)

[별첨] 헬싱키 선언]

헬싱키 선언
# - 인간을 대상으로 하는 의학연구에 있어서의 윤리 원칙

A. 머리말

1. 세계 의학 회의는 헬싱키 선언을 통해 인체를 이용한 의학연구에 관여하는 의사 및 연구자들이 지침으로 삼을 윤리 원칙을 제시해 왔다. 인체를 이용한 의학 연구란 확인 동정이 가능한 인체 시료나 자료에 관한 연구를 포함한다.

2. 인류의 건강을 증진시키고 보호하는 것은 의사의 의무이다. 의사의 지식과 양심은 이러한 의무를 다하기 위해 바쳐져야 한다.

3. 세계 의학 회의<제네바선언>은 <환자의 건강을 나의 첫째가는 관심사로 여길 것이다.>라는 말로 의사의 의무를 촉구하고 있으며, 또<의료윤리에 관한 국제 협약>은 <환자의 신체 및 정신 상태를 약화시킬지도 모를 치료법을 시술할 때, 의사는 오직 환자의 이익만을 고려하여 실시해야 한다.>고 선언하고 있다.

4. 의학의 발전은 궁극적으로 인체를 이용한 실험에 일부분 그 근거를 두고 이루어진다.

5. 인체를 이용한 의학 연구에 있어서 연구대상자의 복지에 대한 고려가 과학적, 사회적인 면의 이익 보다 우선시되어야 한다.

6. 인체를 이용한 의학 연구의 주된 목적은 예방, 진단 및 치료법의 향상과 질병의 원인 및 발생 과정에 대한 이해를 증진시키는 데 있다. 잘 알려진 예방, 진단 및 치료법이라 할 지라도 그 효과와 효능, 유용성 및 질에 대한 지속적인 연구가 행해져야 한다.

7. 현재 행해지고 있는 대부분의 의료와 의학 연구는 예방, 진단 및 치료법들에 있어서 여러 가지 위험과 부담을 수반한다.

8. 의학 연구는 전 인류에 대한 존중심을 증진시키고 인류의 건강과 권리를 보호한다는 윤리 기준에 적합해야 한다. 일부 실험군은 위험에 노출될 수 있으므로 특별한 보호 조치가 필요하다. 경제적, 의학적으로 불우한 처지에 있는 연구대상자가 특히 필요로 하는 것들을 인식하고 있어야 한다. 스스로 동의서를 승인 또는 거부할 능력이 없거나 강제된 상황에서 동의했을 가능성이 있는 경우, 또는 연구를 통해 아무런 개인적 이익이 없거나 연구와 치료가 병행되는 연구대상자에 대해서는 특별한 주의가 필요하다.

9. 연구자들은 인체를 이용한 연구를 할 때 국제적 요구와 더불어 각기 자신의 나라에서의 윤리적, 법적 요구와 규제 사항을 숙지하고 있어야 한다. 그러나 그 어떤 국가의 윤리적, 법적 요구와 규제 사항도 연구대상자의 보호를 위해 이 선언문에서 제시된 사항을 축소하거나 배제할 수는 없다.

B. 모든 의학 연구에 관한 기본 원칙

10. 의학 연구에 있어서 연구대상자의 생명, 건강, 사생활, 존엄성을 보호하는 것은 의사의 의무이다.

11. 인체를 이용한 의학 연구는 일반적으로 공인된 과학적 원칙에 따라야 하고, 과학 문헌과 그 외 관련된 정보를 통한 충분한 지식이 바탕이 되어야 하며, 적절한 실험실적, 그리고 가능한 경우 동물 실험 결과가 근거가 되어야 한다.

12. 환경에 영향을 끼칠지도 모르는 연구를 수행하는데 있어서는 적절한 주의가 요구되며, 실험에 사용하는 동물의 복지가 고려되어야 한다.

13. 인체를 이용한 각 실험 과정의 계획과 수행은 연구 계획서에 분명히 문서화되어야 한다. 이 임상시험 계획서는 심의, 조언, 지도 혹은 필요한 경우 승인을 위해 특별히 구성된 임상시험 심사 위원회에 제출되어야 한다. 이 위원회는 시험자, 의뢰자, 또는 다른 어떤 종류의 부당한 영향력을 끼칠 후 있는 자로부터도 독립적이어야 한다. 이 독립적 위원회는 시험이 수행되는 나라의 법과 규제 사항을 따라야 하며 실험이 진행되는 과정을 조사할 권리가 있다. 시험자는 위원회에 조사 정보, 특히 모든 심각한 이상반응에 대해서 보고할 의무가 있다. 시험자는 또한 심의를 위해 위원회에 기금, 후원자, 관계 기관 그리고 다른 이해관계와 연구대상자 급여에 관한 모든 잠재적 분쟁에 대해서도 보고해야 한다.

14. 임상시험계획서에는 항상 윤리적 고려를 하였다는 사실이 포함되어야 하며 또한 이 헬싱키 선언에서 명시된 원칙에 따랐음을 밝혀야 한다.

15. 인체를 이용한 생체의학 시험은 유능한 임상의의 감독 하에 유자격 과학자에 의해서만 실시될 수 있다. 인체를 이용한 연구에 책임은 유자격 의학자에게 있는 것이지 비록 연구대상자가 동의하였다 하더라도 연구대상자에게 있는 것은 결코 아니다.

16. 인체를 이용한 모든 의학 연구는 연구대상자나 다른 이들에게 미칠 예상 가능한 모든 이익과 위험 및 부담을 세심히 비교 검토한 후에 진행시켜야 한다. 그러나 건강한 지원자가 의학 연구에 참여하는 것을 배제하지는 않는다. 모든 임상시험계획은 공개적이어야 한다.

17. 의사는 인체를 이용하는 시험을 할 때 그 위험성이 적절히 검토되고 충분히 관리될 수 있다고 확신 할 때에만 시험에 착수해야 한다. 잠재적 이익보다 위험이 더 크다고 판단되거나 긍정적이고 이익이 되는 결과에 대한 결정적 증거가 있을 경우엔 그 시험을 중단해야 한다.

18. 인체를 이용한 의학 시험은 그 중요성이 연구대상자가 받을 위험과 부담보다 월등할 때에만 수행되어야 한다. 이는 연구대상자가 건강한 지원자일 경우 특히 중요한 사항이다.

19. 의학 시험은 시험의 결과로써 그 시험이 행해지는 집단이 이익을 얻는다는 타당한 가능성이 있을 때에만 정당화 될 수 있다.

20. 연구대상자는 반드시 지원자이어야 하고 시험에 참여됨을 알아야 한다.

21. 자기 자신의 안전을 지키기 위한 연구대상자의 권리는 존중되어져야 한다. 연구대상자의 사생활을 지키고 개인 정보의 비밀을 보장하며 시험으로 오는 육체적 정신적 충격과 인격에 미치는 영향을 줄이기 위한 모든 주의가 기울여져야 한다.

22. 인체를 이용하는 시험에 있어서는 그 시험 자체의 목적과 방법, 기금의 출처, 모든 가능한 이해 분쟁, 시험자가 속한 기관, 예견되는 이익과 내재하는 위험성, 그리고 그에 따르는 고통 등에 관하여 연구대상자에게 사전에 충분히 알려주어야 한다. 또한 연구대상자에게는 언제든지 아무런 불이익 없이 시험 참여를 그만 둘 자유가 있다는 것을 알려주어야 하고 또 언제든지 그 동의를 철회할 자유가 있음을 주지시켜야 한다. 의사는 연구대상자가 모든 사항을 이해했음을 확인한 뒤에 연구대상자의 자유 의사에 의한 시험 동의를 가능하면 문서화하여 얻어야 한다. 만일 동의서를 문서화하여 얻을 수 없다면 증인 입회 하에 구두로 동의를 얻어 정식 서류화 하여야 한다.

23. 시험 수행에 대한 동의를 얻을 때 의사는 연구대상자가 자기에게 어떤 기대를 거는 관계가 아닌지 또는 그 동의가 어떤 강제된 상황에서 이루어진 것은 아닌지에 대하여 특별한 주의를 기울일 필요가 있다. 만일 그러한 경우라면 동의는 그 연구에 참여하지 않고 연구대상자와 아무런 관계가 없으며 연구에 대한 모든 정보를 알고 있는 의사가 얻도록 하여야 한다.

24. 연구대상자가 법적 금치산자이거나 육체적 또는 정신적 무능력자로 직접 동의를 얻을 수 없을 때, 또는 미성년자일 때는 법에 따라 그 권한을 대행할 수 있는 친족의 동의를 얻어야 한다. 이런 집단의 경우 시험이 그 시험 집단의 건강 증진에 필요한 경우 또는 법적 자격이 있는 이들에게 행해질 수 없는 경우가 아니면 연구 대상이 되어서는 안 된다.

25. 미성년자의 경우처럼 법적 능력이 없는 것으로 간주되나 사실상 시험 참여 의사를 밝힐 수 있는 경우 시험자는 법적 권한을 대행할 수 있는 친족의 동의와 더불어 본인의 동의도 함께 얻어야 한다.

26. 대리 동의와 사전 동의를 포함하여 동의서를 받을 수 없는 개인에 대한 시험은 동의서를 받을 수 없는 육체적/정신적 상황이 실험에 필요 사항일 경우에만 행해져야 한다. 동의를 받을 수 없는 상황에 있는 연구대상자를 시험 대상으로 하는 이유에 대해서는 심사 위원회의 심사와 승인을 위해 제출된 임상시험계획서에 명시되어야 한다. 계획서에는 각 개인이나 법적 대리인으로부터 시험에 계속 참여한다는 동의를 가능한 빨리 받아야 함도 명시해야 한다.

27. 저자와 출판업자 모두에게는 윤리적 책임이 있다. 연구 결과를 잡지에 발표할 때 시험자는 정확성을 기해야 한다. 긍정적 결과뿐만 아니라 부정적 결과 역시 잡지에 발표되거나 공개되어야 한다. 기금의 출처, 관계 기관 그리고 모든 가능한 이해 분쟁도 잡지 발표 시에 공표되어야 한다. 이 선언에서 규정된 원칙에 어긋나는 시험은 잡지 게재가 수락되지 않아야 한다.

C. 치료를 겸한 의학 연구에 관한 부가 원칙

28. 의사는 그 시험이 누가 보아도 질병의 예방, 진단 및 치료에 도움이 된다고 인정되는 범위 내에서만 진료를 겸한 의학 시험을 할 수 있다. 진료를 겸한 의학 시험을 할 때에는 연구대상자가 되는 환자를 보호하기 위한 부가 규정을 따라야 한다.

29. 새로운 방법을 채택할 때 얻어질 수 있는 이익과 위험, 부담 그리고 효과 등은 현재 적용하고 있는 가장 좋은 예방, 진단 및 치료법과 잘 비교 되어야 한다. 이 사항은 알려진 예방, 진단 및 치료법이 없는 경우에 위약을 사용하거나 아무 처치를 하지 않는 경우에도 해당된다.

30. 시험의 결론 단계에 있어서, 시험에 참여한 모든 환자는 그 연구를 통해 가장 좋은 예방, 진단 및 치료법에 접근했음을 확신할 수 있어야 한다.

31. 의사는 환자에게 행해지는 치료 중 어떤 부분이 시험과 관계되는지를 충분히 알려줘야 한다. 환자가 시험에 참여하는 것을 거절하였다 하여 의사와 환자와의 관계에 지장이 생겨서는 안

된다.

32. 환자를 치료함에 있어 알려진 예방, 진단 및 치료법이 없거나 효과적이지 않은 경우, 그것이 생명을 구하고 건강을 증진시키며 고통을 경감시키는데 도움이 된다고 판단될 경우 의사는 환자의 동의하에 증명되지 않았거나 새로운 예방, 진단 및 치료법을 자유로이 사용할 수 있어야 한다. 가능하면 이 새로운 방법들은 시험 대상이 되어야 하며 그 안전성과 유효성을 밝힐 수 있도록 계획되어져야 한다. 모든 경우에 있어서 새로운 정보는 기록으로 남겨져야 하며 가능하면 논문으로 발표되어야 한다. 그 외 이 선언문에서 제시한 다른 관계 지침들도 따라야 한다.
