# Supplementary material for: Effectiveness of electroacupuncture and acupuncture in alleviating cold hypersensitivity in the hands and feet: A randomized controlled trial
Source: PLoS One. 2024 Nov 13;19(11):e0313789. doi: 10.1371/journal.pone.0313789 (PMC11559979; doi:10.1371/journal.pone.0313789)
Supplement: S4 File — (DOCX) [file pone.0313789.s004.docx]

**Multicenter Clinical Study of the Efficacy and Safety of Electroacupuncture for Cold Hypersensitivity in the Hands and Feet**

**Ver 2.1**

**A multicenter clinical study of the efficacy and safety of electroacupuncture treatment for cold hypersensitivity in the hands and feet**

**- Randomized, controlled clinical trials -**

**Protocol Number: Acu 2019_05**

Researcher: Sangji University Industry Academy Cooperation Foundation

Principal Investigator: Jun-Sang Yu

Co-Principal Investigator: Dong-Nyung Lee, Dong-Il Kim

Document type: Clinical trial protocol

Protocol Created: May 15, 2019

Ver: 2.1

Cold Hypersensitivity in the Hands and Feet Clinical Practice Guideline or Korean Medicine Development Team

**CONFIDENTIAL**

All information related to this protocol is confidential.

It may not be disclosed to any third party without the consent of the Guideline Development

Team

## **Clinical Protocol Overview**

| Title | A multicenter clinical study of the efficacy and safety of electroacupuncture treatment for cold hypersensitivity in the hands and feet  - Randomized, controlled clinical trials - |
| --- | --- |
| Objective | To objectively compare and evaluate the efficacy and safety of acupuncture or electroacupuncture in relieving cold feet in patients with cold feet in a multicenter, randomized, controlled trial |
| Clinical trial sites | Sangji university korean medical hospital  Semyung university korean medical hospital at Chungju  Dongguk university korean medical hospital at Ilsan |
| Principal Investigator | Prof. Jun-Sang Yu, Sangji university korean medical hospital  Prof. Dong-Nyung Lee, Semyung university korean medical hospital at Chungju  Prof. Dong-Il Kim, Dongguk university korean medical hospital at Ilsan |
| Clinical trial sponsor | Sangji University Industry Academy Cooperation Foundation  Address: 80, Sangjidae-gil, Wonju-si, Gangwon-do, Republic of Korea |
| Test Design | Randomized, multicenter, controlled trial |
| Trial  duration | 24 months from each institution's IRB approval date |
| Number of study participants | Total 72 participants (24 in the electroacupuncture treatment group, 24 in the acupuncture treatment group, and 24 in the no-treatment control group)  Sangji university korean medical hospital: 18 participants  Semyung university korean medical hospital at Chungju: 24 participants  Dongguk university korean medical hospital at Ilsan: 30 participants |
| Target disease | Cold hypersensitivity in the hands and feet |
| Clinical trial interve ntions | Electroacupuncture atment group: acupuncture points (bilateral TE5, LI4, LR3, SP6) + electroacupuncture treatment  Acupuncture group: acupuncture points (bilateral TE5, LI4, LR3, SP6)  No treatment control group |

| Intervention Period | Acupuncture treatment group: Twice a week for 5 weeks, 10 sessions, 15 minutes  per session  Electroacupuncture treatment group: Electroacupuncture stimulation for 15 minutes per session, twice a week, for a total of 10 sessions for 5 weeks  No treatment control group: Week 1, Week 5, Week 9, for a total of 3 visits |
| --- | --- |
| Study inclusion criteria | 1. Women between the ages of 19 and 59 with coldhypersensitivity in the hands and feet 2. Women who meet the definition below, and at least one of the scope items   (1) Definition: A person who complains of discomfort due to cold hands and feet  (2) Scope items  First, experience coldhypersensitivity in the hands and feet at temperatures where you would not normally feel cold  Second, experience excessive cold extremities compared to others around you at temperatures that make you feel cold  Third, symptoms do not recover easily when moving from a cold to a warm environment   1. Visual Analog Scale (VAS) of 4 or greater for coldhypersensitivity in the hands and feet at the time of the screening visit 2. At the time of the screening visit, after 10 minutes of room temperature acclimatization to 24°C (±2) in the upper and lower extremities, measured by a thermometer, the temperature difference between the palm (PC8) and arm (LU4) is greater than or equal to 0.3°C, or the temperature difference between the anterior thigh (ST32) and dorsum of the foot (LR3) is greater than or equal to 2°C 3. Who can be followed during clinical trials 4. Agree to participate and sign an informed consent form after receiving a clear explanation of the purpose and nature of this study |
| Study exclusion criteria | 1. Taking calcium antagonists or beta-blockers for the treatment of coldhypersensitivity 2. Having one or more ulcers or gangrene on fingers 3. Diagnosed with hypothyroidism or taking thyroid medications 4. Diagnosed with an autoimmune disease 5. Having been diagnosed with carpal tunnel or tarsal tunnel syndrome, or have a positive phalen-tinel test 6. Diagnosed with a herniated disc in the cervical or lumbar spine 7. Diagnosed with diabetes 8. Taking medications that may affect hand and foot coldness (e.g., anticoagulants) 9. Patients with severe hepatic disease (AST-ALT >100 IU/L each) or renal failure (Creatinine >2.0 mg/dL) 10. Behavioral disorders or depression, anxiety, and serious psychiatric disorders 11. Adult women (non-pregnant) Hemoglobin less than 7 g/dL, WBC greater than 11,000/mm^3^ 12. Average systolic blood pressure of 180 mm Hg or diastolic blood pressure of 100 mm Hg or higher, measured twice 13. Having a heart condition, such as arrhythmia or ischemic heart disease, that requires treatment on an electrocardiogram 14. Alcohol or drug abusers 15. Pregnant (positive urinary hCG), nursing mothers, women of childbearing potential who are planning to become pregnant or who do not agree to use adequate contraception 16. Having been diagnosed with or treated for a malignancy 17. Participating in another clinical study or within 2 months of completing a clinical study 18. Refused to participate in a clinical trial or provide informed consent 19. Those with limited ability to understand or express Korean 20. Anyone who, in the judgment of the investigator, is deemed unsuitable for the study |

| Concomitant Medications /Therapi es | 1. No new medications are administered during the trial 2. However, they may be administered as needed at the discretion of the investigator. Participants will be excluded if they are taking any medications that, in the opinion of the investigator, may affect the endpoints of this study (e.g., antithrombotic agents (including antiplatelet agents and anticoagulants), psychotropic medications, antidepressant medications, medications related to hyperthyroidism, dietary supplements (red ginseng), OTC blood flow enhancers, medications that may affect tarsal tunnel, etc.) 3. Participants must not take any additional medications during the study period, including Example) Antithrombotic medications (including antiplatelet and anticoagulant medications), psychotropic medications, antidepressant medications, medications related to hyperthyroidism, dietary supplements (red ginseng), OTC blood flow enhancers, and medications that have the potential to affect tingling feet. |
| --- | --- |
| Methods | 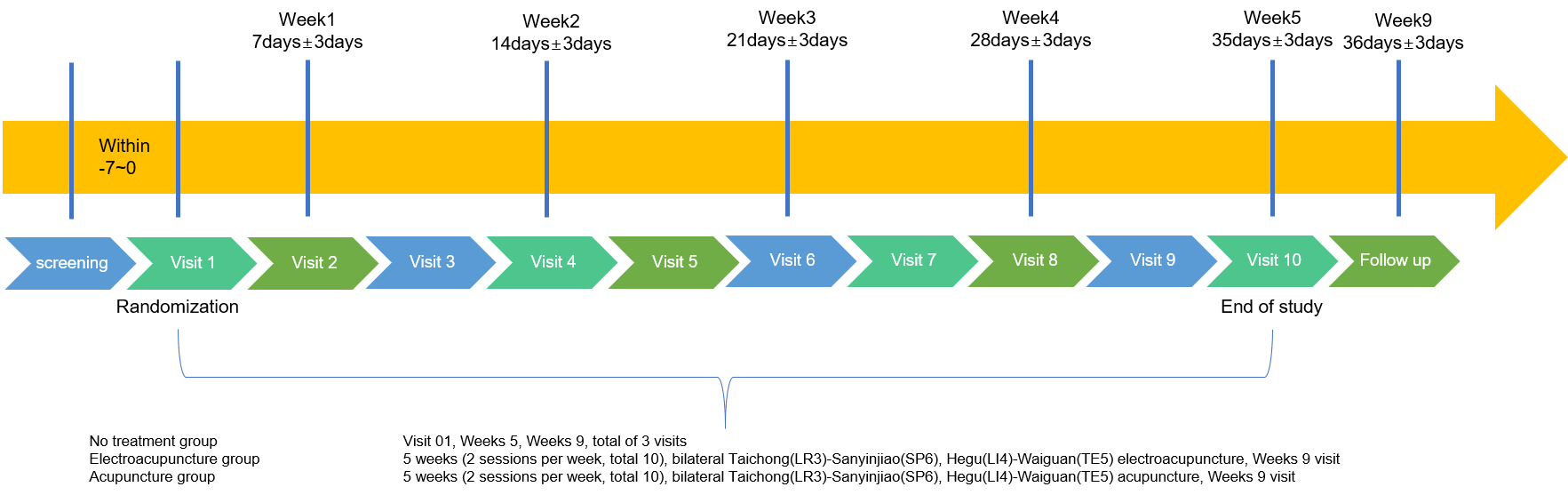  This study is a randomized, multicenter, parallel design, clinical trial. All participants in this study will be fully informed, signed informed consent, assigned a study box identification code, and randomized to one of three arms: no-treatment control, acupuncture treatment, or electroacupuncture treatment. Each participant will visit each study site 10 times, including a screening visit, randomization, and then twice a week for a total of 10 visits. This is a total of 12 visits including screening. The study duration is 5 weeks, with a follow-up visit 4 weeks after the end of the study. |
| Treatment adherence | Drop out if adherence to acupuncture treatment is less than 70% out of 10 visits. |
| Validation methods | 1. Primary outcome variable:   Visual Analog Scale (VAS) of coldhypersensitivity in the hands  Visual Analog Scale (VAS) of coldhypersensitivity in the feet   1. Secodary outcome variable:   (1) Body temperature: Thermometer measurement  (2) Pattern Identification Questionnaire, Coldness Diagnostic Questionnaire  (3) Quality of Life Questionnaire (WHOQOL-BREF) |
| Evaluation of safety | Vital Signs (at every visit), Hematology (WBC, RBC, Hemoglobin, platelet), Blood Biochemistry (BUN, Creatinine, AST, ALT, r-GTP) (at the end of trial)  Safety will be evaluated based on the results of the investigator's examination, adverse event monitoring, and clinicopathologic examination |

| Statistical analysis | | 1. Analytics for validation variables 2. Comparing baseline demographic and assessment variables   To test whether the distributions of variables in the test and control groups are homogeneous, analysis of variance (ANOVA) or nonparametric methods are used for continuous variables, and the Chi-square test is used for categorical variables.   1. Primary efficacy evaluation variable 2. Visual Analogue Scale (VAS) of coldhypersensitivity in the hands and feet   The analysis of the primary efficacy evaluation variable is presented in the ITT group as the main analysis group, and the results of the PP group are presented in addition. ANOVA, repeated ANOVA, paired t-test, etc. will be conducted to test whether there is a difference in the change in VAS score between the no-treatment control group, acupuncture treatment group, and electroacupuncture treatment group after the intervention. Comparison of the degree of improvement of VAS scores within each group will be performed by paired t-test using the difference between pre- and post-intervention VAS scores (Weeks 5 ± 3days VAS score - VAS score at Visit 1).   1. Secondary Validation Variables 2. Body Temperature (BT; thermometer measurement): To test whether there is a difference in BT change between the acupuncture treatment group and the electroacupuncture treatment group, repeated measures ANOVA will be performed. To compare the degree of improvement in BT within each group, a paired t-test will be conducted using the difference between BT before and after acupuncture treatment (BT at Weeks 5±3 days - Visit 1 BT). 3. WHOQOL-BREF Quality of Life Assessment Questionnaire: ANOVA and post hoc tests will be used to test the differencse between each group. Intra-group compartisons will be performed by paired t-test using the differences in values before and after electroacupuncture treatment (value at Weeks 5±3 days - value at Visit 1). 4. Pattern Identification Questionnaire: Figure 2 shows the distribution of the dialectic indicators in participants with coldhypersensitivitiy in the hands and feet. To determine the relationship between the dialectical indicators and the change in symptom VAS, ANOVA tests (or non-parametric tests if not normally distributed) and post hoc tests will be performed with the change in symptom VAS as the dependent variable for each indicator.   Validity and reliability of the basic instrument were evaluated. The validity evaluation is  a. Construct validity: The construct validity is evaluated by conducting a factor analysis of the items of the basic dialectic tool. After obtaining the scores for each item, a correlation matrix between the items is created. The number of factors is determined by the eigenvalue of the factor, the factors are rotated after checking the job offers, and items with large factor loadings related to the rotated factor are interpreted and named based on the content of the items.  b. Predictive validity: The correlation between the VAS values and the scores of the factors explaining coldhypersensitivitiy in the hands and feet is evaluated. The degree of correlation between the VAS value and the sum of the scores of the questions that can well explain the coldhypersensitivity is estimated. To evaluate the questionnaire, Cronbach's alpha is calculated. In addition, we will analyze the change in the dialectic items before and after the treatment, the change in the degree of each item, and the size of the treatment effect for each specific dialectic type. Cluster analysis or factor analysis will be used to identify the association and relevance of the dialectic items related to cold hypersensitivity in the hands and feet. | |
| --- | --- | --- | --- |
| Statistical analysis | 1. Coldness Diagnostic Questionnaire:   Cronbach's alpha will be used to analyze reliability. We will also use the Item-Total Correlation statistical method of to analyze the correlation between items by measuring the Pearson correlation coefficient between the total scores of the remaining items. Additionally, cluster analysis and factor analysis will be performed.   1. Analysis of safety evaluation variables 2. Clinical laboratory test data will be analyzed using appropriate statistical methods, including within-group comparisons before and after treatment, depending on the nature of the variable. Results will be reported in terms of frequency, prevalence, list of each event, detailed time of onset, severity, and causal relationship to acupuncture treatment, and if necessary, presented in graphical form. Statistical analysis will be performed using ANOVA, t-test, chi-square, or Fisher's exact test, depending on the nature of the variable. 3. Adverse events, including self-reported anesthesia: All adverse events will be categorized by treatment group and body system and tabulated with detailed descriptions. The frequency of adverse events causally related to acupuncture treatment and those not causally related will be recorded for each group. The number of adverse events and the proportion of participants experiencing at least one adverse event within each group will be determined with 95% confidence intervals and compare between groups. 4. Clinical pathology tests: Descriptive statistics will be presented for each group and visit for continuous data such as hematology and blood biochemistry test results and vital signs. Differences between visits will be analysed using paired t-test or non- parametric methods. 5. Analyzing missing values 6. In the statistical analysis, the validity endpoint will be analysed using the Last Observation Carried Forward (LOCF) method, which substitutes the measurement value from the previous trial for any observation that is not observed for some reason, i.e., a missing value. For safety evaluation variables, missing values will be treated as missing and analysed accordingly. | |  |

**Table of Contenst**

[<Summary of Clinical Study Schedule >](#_bookmark0) 1

1. [Names of the trial and phase](#_bookmark1) 3
2. [Clinical site name and address](#_bookmark2) 3
3. Name of clinical trial contact person and summary of contract research organization duties by human application site 3
4. [Sponsor name and address](#_bookmark3) 4
5. [Background and purpose](#_bookmark4) 4
6. [About acupuncture treatments](#_bookmark5) 6
7. [Inclusion/exclusion criteria and the number of participants targeted and the rationale](#_bookmark6) 9
8. [Trial duration](#_bookmark7) 11
9. [Test Methods](#_bookmark8) 11

Clinical trial flow chart 17

1. [Observation and inspection items](#_bookmark9) 18
2. [Predicted adverse events and precautions for use of acupuncture](#_bookmark10) 29
3. Study discontinued, study participants exclusion criteria[Criteria](#_bookmark11)  29
4. Criteria for evaluating safety, how it is evaluated, and how it is respond to 30
5. [How](#_bookmark13) to evaluate validity and how to interpret 34
6. [Statistical Methods](#_bookmark14) 36
7. Handling of adherence and protocol violation in clinical trials 38
8. Resource management 38
9. Informed consent form 39
10. [Protocols for victim compensation](#_bookmark18) 39
11. [Standards of care and treatment for post-trial research participants (proposed)](#_bookmark19) 39
12. [Measures to protect the safety of human participants and e](#_bookmark20)tc. 39
13. [Other matters necessary to conduct clinical trials safely and scientifically](#_bookmark21) 40
14. [References](#_bookmark22) 41

[Appendix 1] Protocol for victim compensation 42

[Appendix 2] Helsinki Declaration 43

<Summary of Clinical Study Schedule>

|  | | Screening | Treatment period | | | |
| --- | --- | --- | --- | --- | --- | --- |
| Visit | | (D -7~0) | Visit 1  (D 1) | Visits 2-9 | Visits 10  (D 35±3) | Visit11  (D 63±3) |
| Obtain written consent | | ● |  |  |  |  |
| Assign a screening number | | ● |  |  |  |  |
| Chest X-ray & EKG^1)^ | | ● |  |  |  |  |
| Inclusion/exclusion criteria^2)^ | | ● |  |  |  |  |
| Randomization | |  | ● |  |  |  |
| No-treatment control group | |  | ■ |  | ■ | ■ |
| Assessing treatment adherence | |  |  | ○ | ○ | ○ |
| Whether participants drop out of the study | |  |  | ○ | ○ | ○ |
| Vital signs | | ● | ● | ○ | ● | ● |
| Height, weight, and body mass index  (BMI) | | ● |  |  | ● (Weight only) | ● (Weight only) |
| Pathways and motivations to participate in  research | | ● |  |  |  |  |
| Demographic and sociodemographic  surveys, obstetrics and gynecology^3)^ | | ● |  |  |  |  |
| Investigate medical and medication  history^4)^ and identify changes | | ● | ● | ○ | ● | ● |
| Constitutional Diagnosis Questionnaire  (KS-15) | | ● |  |  |  |  |
| Inspect | |  |  |  |  |  |
|  | General physical exam | ● | ● | ○ | ● | ● |
|  | Thermometer measurement^5)^ | ● | ● | ○ | ● | ● |
|  | VAS of coldhypersensitivity in the hands and feet | ● | ● | ○^6)^ | ● | ● |
|  | Screening for and identifying  adverse events |  |  | ○ | ● | ● |
|  | Pattern Identification Questionnaire | ● |  |  |  |  |
|  | Coldness Diagnostic Questionnaire |  | ● |  | ● | ● |
|  | Quality of Life Assessment  Questionnaire (WHOQOL-BREF) |  | ● |  | ● | ● |
|  | Quality of Life Assessment EQ-5D |  | ● |  | ● | ● |
| Laboratory tests | | ●^7)^ |  |  | ○ |  |
| Acupuncture or electroacupuncture | |  | ○ | ○ | ○ |  |
| Specify next visit date | |  | ● | ○ | ● |  |
| Examiner signature | | ● | ● | ● | ● | ● |

● Conduct all three groups

○ Acupuncture treatment group, pre-acupuncture treatment group only

■ No-treatment control visit date

1. Screening includes chest x-ray and electrocardiogram
2. Performed at screening
3. Age, occupation, digestion, exercise, smoking, drinking, sleep, and motivation t o participate
4. Include a medical and medication history related to coldhypersensitivity in the hands and feeet
5. Temperature measured at bilateral anterior thigh (ST32) and dorsum of the foot (LR3) and the palm (PC8) and arm (LU4) sites at each visit
6. The acupuncture group and the electroacupucnture treatment group additionally measured the hands and cold coldness VAS at visit 4 and visit 8.
7. Screening includes hematologic tests (WBC, RBC, Hemoglobin, platelet), blood chemistry tests (BUN, creatinine, AST, ALT, r-GTP, glucose), thyroid function tests (free T4, TSH), urine and urinalysis, and pregnancy test (Urine HCG). At D35 (Visit 10), hematology (WBC, RBC, Hg, platelet), blood
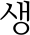
 chemistry (BUN, creatinine, AST, ALT, r-GTP, glucose) will be performed.

#### Name of the trial and phase

Title: A multicenter clinical study of the efficacy and safety of electroacupuncture treatment for cold hypersensitivity in the hands and feet - Randomized, controlled clinical trials -

Phase: Investigator for Clinical Trials

#### Clinical site name and address

- 1. Semyung university korean medical hospital at Chungju

Address: 63, Sangbang 4-gil, Chungju-si, Chungcheongbuk-do, Chungcheongbuk-do, Republic of korea

Phone: +82-43-841-1733, Fax: +82-43-856-1731

- 1. Sangji university korean medical hospital

Address: 80, Sangjiji-gil, Wonju-si, Gangwon-do, Republic of korea

Phone: +82-33-732-2111, Fax: +82-33-732-2124

- 1. Dongguk university korean medical hospital at Ilsan

Address: 27, Dongguk-ro, Ilsandong-gu, Goyang-si, Gyeonggi-do, Republic of Korea

Phone: +82-31-961-9126, +82-31-961-009

#### Name of clinical trial contact person and summary of contract research organization duties by human application site

- 1. Pricipal investigators, co-investigators, study personnel, and medical device managers per site

Principal Investigators, co-investigators, coordinators, investigators, investigational device managers, and monitoring personnel for each site are submitted in the review application or as a separate document for each site.

- 1. Summary of human clinical trial sponsors and tasks

Kyung Hee University Korean Medical Center (23 Gyeonghee-daero, Dongdaemun-gu, Seoul, Republic of Korea)

Role: Protecting the safety and rights of human participants; ensuring the completeness, reliability, and quality of data; ensuring consistency of data across sites where clinical trials are conducted; providing guidelines to monitors on the minimum requirements for monitoring; ensuring that clinical t r i a l s a r e conducted appropriately and in accordance with the International Conference on Harmonization Good Clinical Practice (ICH-GCP), the Declaration of Helsinki, regulations of the relevant authorities, and SOPs agreed to be followed by the sponsor and Kyung Hee University College of Oriental Medicine Clinical Trials Center (K-CTC).

#### Sponsor name and address

- 1. Sponsor: Sangji University Industry Academy Cooperation Foundation
  2. Address: 80, Sangjidae-gil, Wonju-si, Gangwon-do, Republic of Korea

#### Background and Purpose

##### Background

- The concept of coldhypersensitivity
- It is defined as "the sensation of coldness in a specific part of the body despite room temperature when other parts of the body do not feel cold at all" and is medically referred to as "cold intolerance" (Lee SR, et al. A review of the literature on gynecologic cold sensitivity. The journal of oriental obstetrics & gynecology. 1996;9(1):55-80)
- Cold sensation is a condition in which certain parts of the body feel cold and chilled at temperatures that would not normally cause a person to feel cold, making it difficult to maintain normal activities of daily living (Modern Chinese Herbal Medicine, Hansung Publishing House, 1989).
- Coldness is especially prevalent in women, with the frequency of coldness occurring in the whole body, extremities, lower abdomen, pubic area, lumbar area, and abdomen in that order. It is often associated with leukorrhea, postpartum pain, and spontaneous abortion (Bae GM, et al. Research of relationship on cold hypsersensitivity for the patients in OB & GY of Dong Eui Medical center. The journal of oriental obstetrics & gynecology.2002;15(2):101-13).
- The concept of coldness is a symptom not included in the ICD-10. It is a condition that reduces the quality of life for the individual patient, such as cold hypersensitivity in the hands and feet, reluctance to shake hands with others, and the need to wear thick socks even in summer. Effective treatment is required, but there is no clear treatment method in Western medicine, where simple blood circulation administration is used with unknown effectiveness. In contrast, Chinese medicine uses acupuncture, moxibustion, and herbal medicine to treat it, making Chinese medicine a potential primary treatment option. (Choi JH. Guidebook to the practice of oriental medicine. Seoul: Korean Medicine; 2001, p. 284-6.)
- Existing research on cold hypersensitivity (including how to diagnose coldness)
- Standardizing the diagnosis of cold feet with infrared skin thermography

The temperature difference between the infrared thermograms of the lower extremities of 50 patients complaining of cold hypersensitivity in the feet and 50 patients not complaining of cold hypersensitivity in the feet was divided into two groups. The sensitivity was 94.0% and the specificity was 90.0% when the temperature difference between the infrared thermograms of the palm (PC8) and arm (LU4) was more than 0.3℃ in the case of cold feet, and the validity of the diagnosis of cold feet was recognized. In the case of feet cold, the anterior thigh (ST32) and dorsum of the feet (LR3) showed a sensitivity of 94% and a specificity of 76% when the temperature difference was greater than 2.0℃. (Kim DH, et al. standardization of diagnosis of cold hypersensitivity of hands and feet by D.I.T.I.. The journal of oriental obstetrics & gynecology. 2001;14(2): 120-134)

- Diagnosis of coldhypersensitivity with the cold-loading test

Cold-loading testing of the hand has been performed since 1976 and has been used to diagnose Raynaud's syndrome, peripheral circulatory disorders in diabetics, vascular disorders caused by smoking or vibration exposure, and RDS syndrome. This has been done mainly by thermography, but other methods include high-frequency ultrasound and cold-loading testing using a digital hemodynamic meter. (Han JY, et al. Cold stress test for the diagnosis of cold hypersensitivity on hands. Journal of oriental medical thermology. 2003;2(1):17-23)

- Clinical evaluation of infrared skin thermography and peripheral nerve testing for the diagnosis of coldhypersensitivity

In an electrical sensory perception test measured at the median, ulnar, and radial nerves in the hand between mild and severe cold, the electrical sensation was duller in the severe cold group. The mean electrical sensory perception values for each frequency at the peroneal, tibial, and saphenous nerves in the foot between mild and severe cold were also duller in the severe cold group. (Lee KS. A Clinical Evaluation of DITI and Neurometer for the Diagnosis of Cold Hypersensitivity. Journal of oriental medical thermology. 2004;3(1):60-66)

- Characterizing heart rate variability in patients complaining of cold illness

In Chinese medicine, maintaining a normal body temperature is due to the thermoregulatory action of qi, but if the warming and cooling action of qi is impaired due to the effects of chiljeong, exopathic disease, or overwork, the thermoregulatory action is lost and coldness occurs. Therefore, the characteristics of cold patients were identified through HRV, and it was found that HRV testing can be helpful in diagnosing and providing prognosis of cold patients. (Lee MJ, et al. Analysis of Heart Rate Variability in Cold Hypersensitivity Females Visiting Gangnam Kyung-Hee Korean Hospital. The journal of oriental obstetrics & gynecology. 2011;24(3):109-115)

- Using infrared thermography to examine the association of defecation habits with lower abdominal and cold hypersensitivity in the hands and feet

As a result of investigating whether cold feet and abdominal coldness are related to bowel habits by comparing the temperature difference between the lower extremities and abdomen, it was found that cold feet were more severe in the constipation group, general group, and diarrhea group. Feet coldness was also more severe in the constipation group, general group, and diarrhea group. Feet coldness was also more severe in the constipation group, thus securing clinical evidence that cold feet and diarrhea are related. This was helpful in studying the relationship between cold feet and functional growth disorders. (Yoon SW, et al. Clinical research about the correlation between defecation type and cold hypersensitivity of lower of abdomen, hand and Foot. Journal of oriental medical thermology. 2004;3(1):36-42)

- A study of infrared thermographic imaging characteristics of patients with postpartum pain

This study objectively examined the differences between postpartum patients, who often feel tingling and cold in their body parts, and the general population by measuring whether their body temperature is actually cold with an infrared thermograph. (Park KS, et al. A Study on Digital Infrared Thermographic Imaging Characters of Women Suffering from Postpartum Disease. The journal of oriental obstetrics & gynecology. 2010;23(2):116-123)

- Study of infertile women's body heat with infrared imaging

In the infertility group, the temperature of the body surface was measured with an infrared camera. It was found that the temperature of the infertility group was lower than the general population in the lower abdomen and in other areas. The temperature of the knees and waist was also lower than the general population. (Kim HW, et al. Correlation between Women Infertility and DITI. Journal of oriental medical thermology. 2002;1(1):52-56)

- Prescribing circulation enhancers for coldhypersensitivity: 60% of physicians who encountered coldhypersensitivity prescribe circulation enhancers (Kim CM, et al. Recognition of family practitioners on cold hands/feet syndrome and Raynaud’s Disease. J Korean Acad Fam Med. 2007;28:339-45).
- In a study of the association between coldhypersensitivity in the hands and feet and circulatory disorders, erythrocyte deformation capacity was examined as a diagnosis of microcirculatory disorders. It was found that erythrocyte deformation capacity was lower in patients with coldhypersensitivity in the hands and feet, which is thought to decrease the oxygen delivery capacity of erythrocytes and oxygen delivery to microvessels. (Park JY, et al. The relationship between coldness of hands and feet and the erythrocyte deformability in stroke patients. Korean Journal of Oriental Internal Medicine. 2010;31(3):578-85)
- When treating peripheral neuritis patients with warm acupuncture, the treatment group had 26 cured, 6 improved, and 2 not cured, while the control group had 5 cured, 7 improved, and 19 not cured. (Zhang X, et al. Clinical observation on the treatment of sensory disorders in peripheral neuritis with warm needle moxibustion. Xinjiang Journal of Traditional Chinese Medicine. 2007, (06))
- Raynaud's disease is a condition in which blood vessels in the hands and feet are constricted by cold stimuli, causing blood circulation disorders, and acupuncture treatment is effective for Raynaud's disease. This is attributed to the effect of improving blood flow in peripheral blood vessels. The acupuncture points used for treatment include LI4, PC6, TE5, TE4, EX-UE9, HT1, EX-23, LI13, LU5, and LR3, SP6, ST36, EX-LE10, ST41, SP9. (Jeon SW, et al. A review of acupuncture for the treatment of Raynaud’s disease. Korean Journal of Oriental Internal Medicine. 2017;38(4):433-442)

##### Purpose

- - 1. **General purpose**

The purpose of this study is to objectively evaluate the efficacy and safety of acupuncture or electroacupuncture for the relief of coldness in patients with coldhypersensitivity in the hands and feet in a multicenter, randomized, controlled clinical trial.

##### Primary purpose

The efficacy will be evaluated by comparing the change in Visual Analogue Scale (VAS) scores of coldhypersensitivity in the hands and feet after 15 minutes of acupuncture on acupuncture points (LI4-LR3, LR3-SP6), performed twice a week for a total of 10 acupuncture treatments, or by adding electroacupuncture to the same acupuncture points.

##### Secondary purpose

##### The efficacy of the study will be further evaluated by comparing the changes in scores on the Visual Analog Scale of coldhypersensitivity in the hands and feet, body temperature (thermometer measurement), Quality of Life Assessment Questionnaire (WHOQOL-BREF), Quality of Life Assessment EQ-5D, pattern identification questionnaire, and coldness diagnostic questionnaire between the no-treatment control group, acupuncture treatment group, and electro-acupuncture treatment group.

##### Safety assessment

Safety is assessed through adverse events, clinical laboratory tests, and medical examinations.


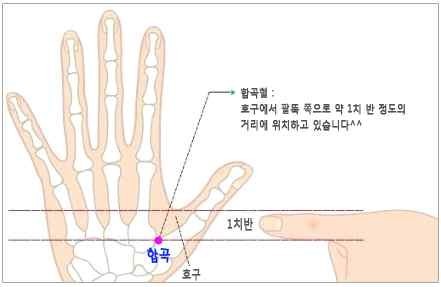


#### About for acupuncture treatments

##### Acupuncture points

###### Acupuncture and electroacupuncture groups

- 1. Hegu (LI4)

Location: On the dorsum of the hand, radial to the midpoint of the 2nd metacarpal bone


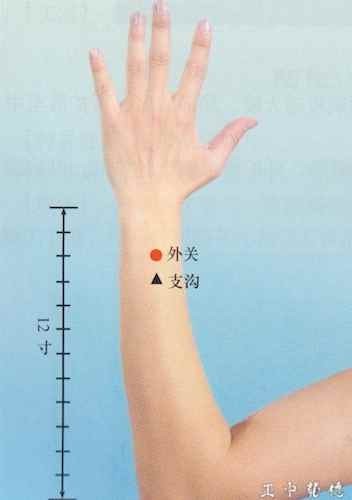
Efficacy: Relieving fever, dispersing wind, clearing away wind, clearing lung-qi, relieving digestive distress, pain and tranquilizing the mind

- 1. Waiguan (TE5)

Location: On the posterior aspect of the forearm, midpoint of the interosseous space between the radius and the ulna, 2 B-cun proximal to the dorsal wrist crease

Efficacy: Clearing heat and detoxifying, invigorating qi, removing congestion heat from triple energizer meridian

- 1. Taichong (LR3)


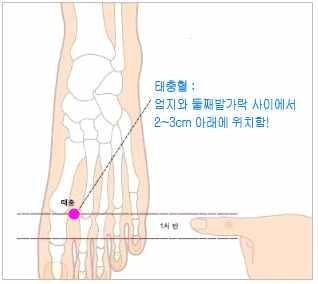
Location: On the dorsum of the foot, between the first and second metatarsal bones, in the depression distal to the junction of the bases of the two bones, over the dorsalis pedis artery

Efficacy: Extinguish liver fire and triple energizer meridian, regulating liver Qi, promoting blood circulation

- 1.
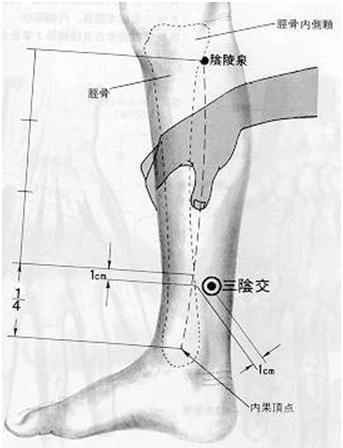
Sanyinjiao (SP6)

Location: On the tibial aspect of the leg, posterior to the medial border of the tibia, 3 B-cun superior to the prominence of the medial malleolus.

Efficacy: Strengthening the spleen, promoting circulation, clearing stagnation of Qi, circulating and regulating the blood, dispelling wind-dampness for the meridians

##### B. Needles, electroacupuncture devices, and treatments used in clinical trials

###### Needle type

0.20 × 30 mm sterile needle (Dongbang Medical, South Korea)

###### Packaging, labeling, and storage

Single-use, sealed, stored at room temperature in the clinical center

###### Clinical trial electroacupuncture devices

The electrospinning machine utilizes an STN-330 (Stratech, South Korea). Details in Appendix.

###### How to use the electrostat

- Prepare

1. Connect the power cord on the back of the main unit to a power source, and turn on the main switch.
2. Confirm that all output dials are set to "0" and that the instrument is operating normally.

- When performing electroacupuncture stimulation.

1. Verify that the dedicated connecting wires used to connect the electroacupuncture devices are free of any cosmetic damage that would cause the sheath to peel off, and that each wire has a dedicated red and black clip attached to it.
2. Securely connect the dedicated connection wire to the output of the electroacupuncture devices (electroacupuncture device cord).
3. Attach the dedicated red and black clips on a dedicated connection wire to connect to the sterile needle.
4. Press the power switch, then set the desired Hz, time, or program.
5. Slowly turn the valve switch to adjust the treatment to the appropriate level of intensity for the patient.


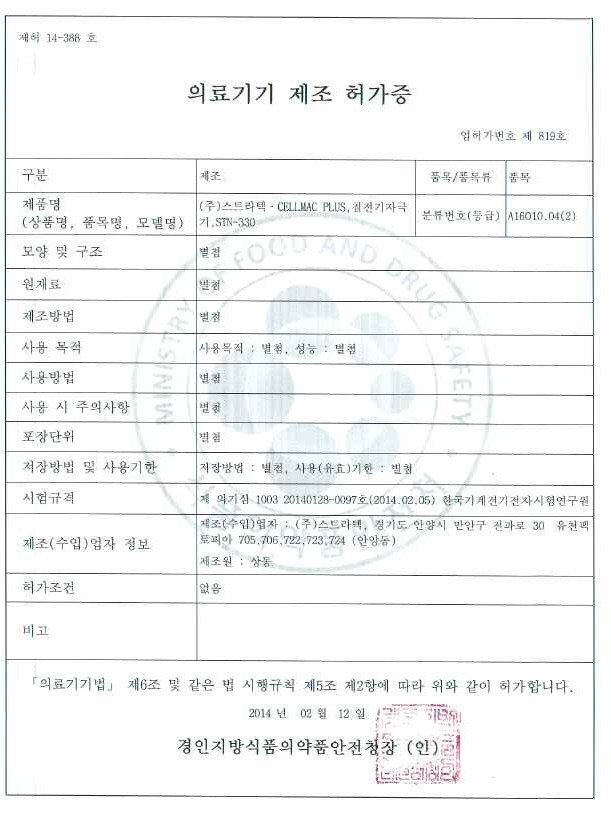


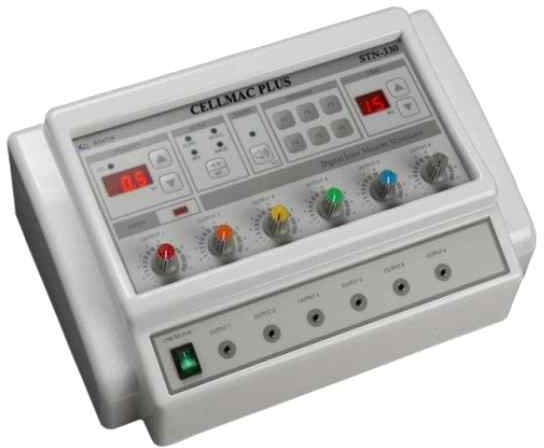

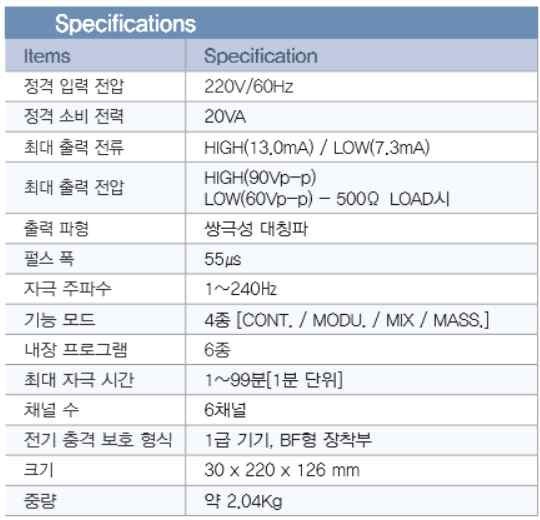


Figure 1. Electroacupuncture device (STN 330) Specifications and Manufacturing License

###### Electroacupuncture Stimulation Methods

- 1. Electroacupuncture stimulation:
     - The acupuncture treatment group consists of 8 points on the bilateral Taichong (LR3), Sanyinjiao (SP6), Hegu (LI4), and Waiguan (TE5), needled at a depth of 10 to 25 mm subcutaneously, and left for 15 minutes. Do not use an electroacupuncture machine.
     - The electroacupuncture treatment group is composed of 8 points on the bilateral Taichong (LR3), Sanyinjiao (SP6), Hegu (LI4), and Waiguan (TE5), needled at a depth of 10 to 25 mm subcutaneously. The Taichong (LR3) is connected to the Sanyinjiao(SP6), and the Hegu (LI4) is connected to the Waiguan (TE5) with a dedicated clip connected to the electroacupuncture needle (red and black color without distinction).
     - Press the power switch of the electroacupuncture device, set the frequency to 2 Hz and the stimulation time to 15 minutes in the on-screen settings, and slowly turn the switches of the individual connecting wires to the right to adjust the intensity to an appropriate level that does not cause pain to the patient.
     - The frequency of treatment is twice a week for a total of 10 acupuncture sessions for 5 weeks, along with electroacupuncture treatment.

##### C. Acupuncture precautions

Care should be taken during acupuncture treatment as it can cause pain, blood clots, tingling sensation, etc.

#### Inclusion/exclusion criteria and the number of participants targeted and their rationale

##### Selection Criteria

It must meet all of the following criteria

1. Women between the ages of 19 and 59 with coldhypersensitivity in the hands and feet
2. Women who meet the definition below, and at least one of the scope items
   - - 1. Definition: A person who complains of discomfort due to cold hands and feet.
       2. Scope items

First, experience coldhypersensitivity in the hands and feet at temperatures where you would not normally feel cold

Second, experience excessive cold extremities compared to others around you at temperatures that make you feel cold

Third, symptoms do not recover easily when moving from a cold to a warm environment

1. Visual Analog Scale (VAS) of 4 or greater for coldhypersensitivity in the hands and feet at the time of the screening visit
2. At the time of screening visit, after 10 minutes of room temperature acclimatization to 24℃ (±2) in the upper and lower exterimities, measured by a thermometer, the temperature difference between the palm (PC8) and arm (LU4) is greater than or equal to 0.3℃, or the temperature difference between the center of the anterior thigh (ST32) and dorsum of the foot (LR3) is greater than or equal to 2℃
3. Who can be followed during clinical trials
4. Agree to participate and sign an informed consent form after receiving a clear explanation of the purpose and nature of this clinical study

##### B. Exclusion Criteria

Individuals who have any of the following conditions are not eligible to participate in this study

1. Taking calcium antagonists or beta-blockers for the treatment of coldhypersensitivity
2. Having one or more ulcers or gangrene on fingers
3. Diagnosed with hypothyroidism or taking thyroid medications
4. Diagnosed with an autoimmune disease
5. Having been diagnosed with carpal tunnel or tarsal tunnel syndrome, or have a positive phalen-tinel test
6. Diagnosed with a herniated disc in the cervical or lumbar spine
7. Diagnosed with diabetes
8. Taking medications that may affect hand and foot coldness (e.g., anticoagulants)
9. Patients with severe hepatic disease (AST-ALT >100 IU/L each) or renal failure (Creatinine >2.0 mg/dL)
10. Behavioral disorders or depression, anxiety, and serious psychiatric disorders
11. Adult women (non-pregnant) Hemoglobin less than 7 g/dL, WBC greater than 11,000/mm^3^
12. Average systolic blood pressure of 180 mm Hg or diastolic blood pressure of 100 mm Hg or higher, measured twice
13. Having a heart condition, such as arrhythmia or ischemic heart disease, that requires treatment on an electrocardiogram
14. Alcohol or drug abusers
15. Pregnant (positive urinary hCG), nursing mothers, women of childbearing potential who are planning to become pregnant or who do not agree to use adequate contraception
16. Having been diagnosed with or treated for a malignancy
17. Participating in another clinical study or within 2 months of completing a clinical study
18. Refused to participate in a clinical trial or provide informed consent
19. Those with limited ability to understand or express Korean
20. Anyone who, in the judgment of the investigator, is deemed unsuitable for the study

##### C. Number of participants

1. Targeted number of participants

|  | Acupuncture  group | Electroacupuncture  group | No treatment group  (control group) | Total number of participants |
| --- | --- | --- | --- | --- |
| Number of eligible evaluations | 18 | 18 | 18 | 54 |
| Number of people considering a  25% dropout rate | 24 | 24 | 24 | 72 |

1. Calculation Rationale

This study was designed to test the hypothesis that acupuncture treatment of patients with coldhypersensitivity in the hands and feet will improve symptoms in the acupuncture treatment group and the electroacupuncture treatment group compared to the no-treatment control group.

The hypothesis of this study is below

H0 : σ = Δ1 – Δ2 = 0

H1 : σ = Δ1 – Δ2 ≠ 0

H0 : σ = Δ1 – Δ3 = 0

H1 : σ = Δ1 – Δ3 ≠ 0

Δ1: Mean change in VAS score from pre-treatment to post-treatment in acupuncture group

Mean change in VAS score of coldhypersensitivity in the hands and feet at week 10 (within ±3 days) after randomization in the acupuncture group

Δ2: Mean change in VAS score from pre- treatment to post-treatment in the electroacupuncture group

Mean change in VAS score of hand coldness at week 10 (within ±3 days) after randomization in the electroacupuncture group

Δ3: Mean change in VAS score from pre-treatment to post-treatment in untreated control group

Mean change in VAS score of hand coldness at week 10 (within ±3 days) after randomization in the no-treatment group

A clinical trial of coldhypersensitivity in the hands and feet with medical devices, acupuncture and electroacupuncture, are designed to estimate the number of participants based on the VAS information form the oxygen chamber device trial.


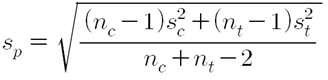
In the referenced trial by Ha et al., the mean VAS difference for the placebo group was -0.48 with a standard deviation of 1.03, and the mean VAS difference for the oxygen chamber group was -1.52 with a standard deviation of 1.17. The expected difference between acupuncture treatments for coldhypersensitivity in the hands and feet is 1.04, and the standard deviation is estimated to be 1.102 using the joint standard deviation formula.

Figure 1. Joint Standard Deviation Formula

The significance level (α), the probabiiity of committing a TypeⅠerror, was set at 0.05, and the probability of committing a TypeⅡerror (β) was assumed to be 0.2. The number of participants in the electroacupuncture treatment group and the no-treatment control group was set to be the same, and the formula for estimating the number of participants by comparing the mean of the two groups was as follows.


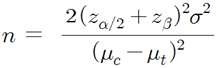


N = [2*(1.96+0.84)^2*1.17^2]/(1.04)^2=17.625 ≒ 18

Based on the above, the number of participants is 18 in each arm. Considering a 25% dropout rate, 24 participants will be enrolled per arm.

18 ÷ (1-0.25) =24

Therefore, we plan to enroll 24 participants in each of the acupuncture treatment group, electroacupuncture treatment group, and no-treatment control group for a total of 72 participants to be analysed.

#### Trial duration

The study duration is set for 24 months from the date of Institutional Review Board (IRB) approval. However, this period may be extended based on the pace of participants enrollment.

#### Test Methods

##### Test design

This study is a randomized, multicenter, parallel design, clinical trial. After receiving sufficient explanation from the researchers involved in this study and voluntarily signing the consent form, participants who are deemed suitable for the study by reviewing the inclusion and exclusion criteria according to the protocol will be assigned a patient identification code. Blood tests will be performed, and they will be randomized to the acupuncture treatment group, electroacupuncture treatment group, and no-treatment control group at a later screening visit.

Each participant will have a screening visit, followed by randomization. If assigned to the acupuncture arm or the electroacupuncture arm, participants will attend bi-weekly visit for a total of 5 weeks, resulting in 10 visits for acupuncture or electroacupuncture, respectively. Subsequently, a follow-up assessment will be conducted 4 weeks later. The control group is required to attend three visits, designated as Visit 1, Visit 10, and Visit 11, respectively.


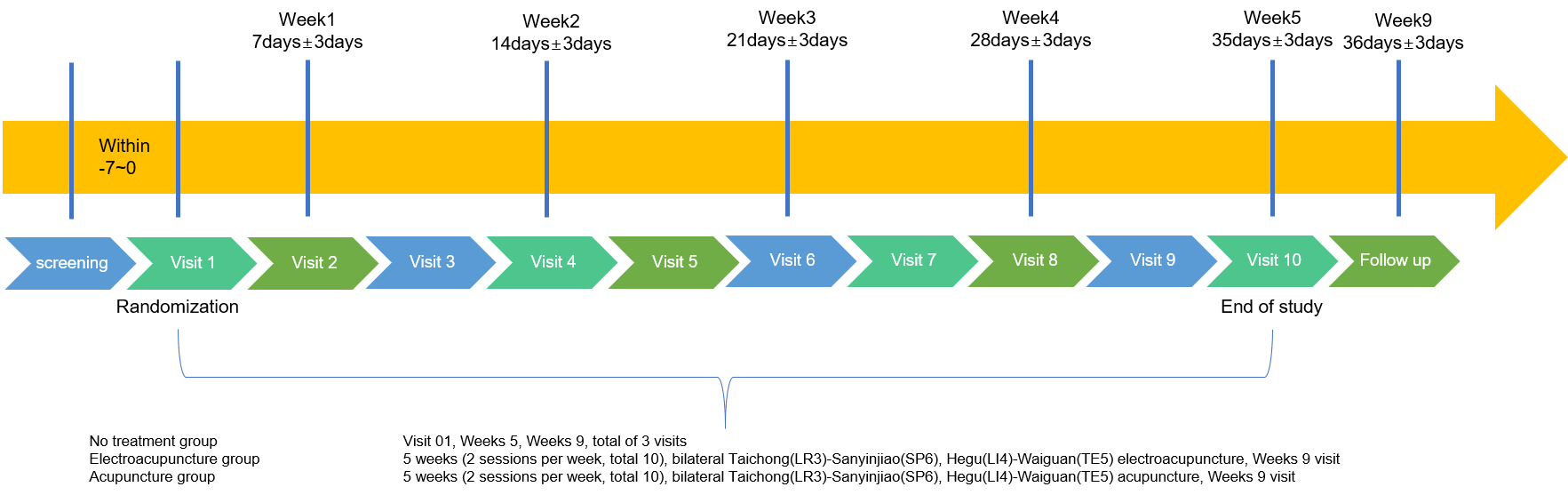


##### Randomization Methods and Blinding

- - 1. Overview

After the particioants have signed the informed consent form, they will be randomized to the acupuncture treatment group, the electroacupuncture treatment group, or the no-treatment control group at Visit 1, if they are deemed eligible for the study after reviewing the inclusion and exclusion criteria. Participants will be instructed on the procedures and visit dates, and will receive 10 treatments twice a week for 5 weeks (±3 days) according to the study schedule. All three arms will be assessed for efficacy 5 weeks (±3 days) after randomization and followed up at 9 weeks (±3 days).

- - 1. Randomization and stratification methods

Patients who agree to participate in this study will be assigned a screening number in the order of their written consent, and if more than one person is screened on a single day, the screening numbers will be assigned in the order of their consent. Randomization codes will be provided by an independent statistician or a third independent investigator with a 1:1:1 ratio of acupuncture, electroacupuncture, and no-treatment control groups. Randomization numbers will be generated using the blockrand package in R 3.4.2 or later, stratified by site at randomization, and block randomization will be used to ensure that the number of participants in each arm is approximately equal. At each site, participants will be assigned numbers based on the order of screening completion.

- - 1. Assignments

This study will use the sealed envelope method. The independent statistician or third independent investigator who generated the randomization code will place a piece of paper with the randomization code inside the opaque envelope, seal it, and give it to the authorized representative at each site. If a participant who has given written consent passes the screening assessment, the authorized representative will open the envelope in the order in which the screening assessment is completed, verify and record the participant's randomization code and assignment arm.

- - 1. Blindfold

This study is an open-label trial in which all arm assignments are not blinded to outcomes. However, we will blind the assessors who evaluate participants' symptoms separately from the treating physician to minimize possible bias in the assessment.

##### Trial Schedule

- - 1. Recruiting trial participants

Volunteers will be recruited through walk-in patients at each site or through open recruitment via outdoor advertising.

- - 1. Consent and assigning participant identification codes

After fully explaining the study, written informed consent will be obtained and a participant identification code will be assigned in this order.

If an employee of the clinical research organization or a student at his/her university wishes to participate, the study will be conducted only on participants unrelated to the study; the study will be fully explained to the employee or student who is motivated to participate voluntarily; and the employee or student will be guided to complete and sign an informed consent form only if he/she agrees. (Example: "I am an employee of a hospital or a student at a university, and I have been fully informed about the study and am motivated to participate voluntarily and sign the consent form.")

- - 1. Participants selection
       1. Demographic and sociodemographic information / other pre-treatment characteristics

Assign a screening number and collect demographic and sociodemographic information. The following will be recorded

- Whether and date of written consent
- Participant initials
- Age
- Blood pressure, height, weight
- Occupation
- Digestibility
- Exercise status and related information
- Smoking status and related information
- Drinking status and related information
- Sleep status and related information
- Obstetrics and gynecology information
- Pathways and motivations to participate in research
  - - 1. Medication history for coldhypersensitivity in the hands and feet / Other Organs

History and Medication History for Hand and Foot Cold: Conducted on Visit 0

Items related to Hand and Foot Cold (if applicable)

- Whether or not you've been diagnosed with coldhypersensitivity in the hands and feet
- Duration of cold feet morbidity (months)
- Previous treatment for cold feet

(medication / korean medical treatment (acupuncture / moxibustion / her bal medicine, etc.) / exercise therapy / psychotherapy / other complementary medications)

- Medications and treatments you are taking for coldhypersensitivity in the hands and feet
- Other medical and medication history items
- General physical exam items: Performed at every visit

Sensory, musculoskeletal, endocrine, multiple, urinary, reproductive, digestive, cardiovascular, nervous, dermatologic, and estrogenic systems. Obtain a history of renal, hematologic, lymphologic, respiratory, and other medical and medication history. Any significant findings o n examination at the initial visit should be recorded in the other organ systems section of the case record sheet, and any significant medical findings that meet the definition of an adverse event after initiation of the study procedure should be recorded on the adverse event sheet.

- - - 1. Visual Analog Scale (VAS)

Assess at each visit. At each visit, a VAS for the degree of coldhypersensitivity in the hands and feet will be completed by the participants with the assistance of the investigator.

- - - 1. Pattern Identification Questionnaire and Constitutional Diagnostic Questionnaire (KS- 15)

Administered at Visit 0.

Two principal investigators or a Korean medicine doctor will evaluate the participants.

The evaluator checks the items and marks ◯ on the most appropriate item to indicate the degree of the condition the participant has through the pattern.

The evaluator marks the corresponding pattern identification with a ◯.

- - - 1. Vital signs and height and weight measurements

Vital signs are assessed at every visit. Vital signs include blood pressure, pulse rate, and temperature. Height is measured only at Visit 0, and weight is measured at Visits 0, 10, and 11.

- - - 1. Determining conformance to inclusion/exclusion criteria

At Visit 0, consent, demographic and sociodemographic information, medical and medication history, laboratory tests, pregnancy test and chest x - ray, electrocardiogram, and thermometer measurements to determine if the participant meets the cold diagnostic criteria will be taken.

- - - 1. Check for changes in medical and treatment history

Inquire at each visit whether there have been any changes in medical and treatment history since Visit 0 (Screening). Inquire about any changes compared to what was identified in the drug history screening at Visit 0. If there are any changes in concomitant medications, document them in detail in the case record.

- - - 1. Clinical examination items

Obtain written informed consent and perform a 12-lead EKG, chest x-ray, and examination of the following on Visit 0

①Hematological tests: WBC, RBC, Hemoglobin, Platelet

②Blood biochemical tests: BUN, Creatinine, AST, ALT, r-GTP, Glucose

③Thyroid function test: free T4, TSH

④Urine and urine specimen tests: Blood, Urobilinogen, Bilirubin, Ketone, Protein, Nitrate, Glucose, pH, S.G, LEU, WBC, RBC

⑤Pregnancy test (women of childbearing age): Urine HCG

However, for Visit 10, the following checks are performed

①Hematologic tests; WBC, RBC, Hemoglobin, platelet

②Blood biochemical tests: BUN, Creatinine, AST, ALT, r-GTP

Systolic blood pressure greater than or equal to 180 mm Hg or diastolic blood pressure greater than or equal to 100 mm Hg as measured and averaged from the inclusion/exclusion criteria during Screening at Visit 0, Hemoglobin less than or equal to 7 g/dL for adult women (non-pregnant), WBC greater than or equal to 11,000/mm3, random plasma glucose less than or equal to 50 mg/dL or greater than 250 mg/dL, AST-ALT greater than or equal to 100 IU/L each, Creatinine greater than or equal to 2.0mg/dL, positive urinary hCG, presence of pulmonary TB lesions or pneumothorax other than inactive TB on chest x-ray, or cardiac disease such as arrhythmia or ischemic heart disease requiring treatment on electrocardiogram.

If there are no abnormal findings on the above examination and the patient's symptoms are considered, the patient will be included in the study.

The above tests will be performed by the laboratory of each medical institution participating in the clinical trial, and the korean medical hospital will refer the above tests to the laboratory of the western medical institution for testing.

- - 1. Randomization

Randomize patients to acupuncture treatment, electroacupuncture treatment, and no treatment control.

- - 1. Baseline checks

Baseline measurements of Visual Analogue Scale (VAS), body temperature (thermometer measurement), quality of life questionnaire (WHOQOL-BREF), quality of life assessment EQ-5D, coldness diagnostic questionnaire, weight, and vital signs will be taken from randomized participants. To minimize evaluators’s bias, one of the participating researchers will be assigned to complete the participants surveys and will not be involved in the acupuncture treatment or electroacupuncture treatment groups. The evaluator should not question the participant about the treatment.

- - 1. Needling and electroacupuncture methods:

Treatments for the acupuncture and electroacupuncture groups will be performed by researchers or study personnel who are not involved in the survey and evaluation.

###### Acupuncture group

The standard for the acupuncture group is 10 acupuncture treatments, twice a week for 5 weeks of treatment. Using 0.25 x 30 mm sterile needle (Dongbang Medical, South Korea), needle 8 points on the bilateral Taichong (LR3), Sanyinjiao (SP6), Hegu (LI4), and Waiguan (TE5), at a depth of 10 to 25 mm subcutaneously.

###### Electroacupuncture group

The standard treatment regimen for the electroacupuncture group is 10 electroacupuncture treatments, twice a week for 5 weeks.

Using a 0.25 x 30 mm sterilized needle (Dongbang Medical, Korea), needle 8 points on the bilateral Taichong (LR3), Sanyinjiao (SP6), Hegu (LI4), and Waiguan (TE5), at a depth of 10 to 25 mm subcutaneously. The Taichong (LR3) was connected to Sanyinjiao (SP6), and Hegu (LI4) to Waiguan (TE5) with dedicated clips connected to an electro-stimulator (STN-330, Stratech) (red and black color-coded). The frequency of electrical stimulation is set at 2 Hz, the duration of stimulation is 15 minutes, and the intensity is such that the participant is aware of the stimulation but does not feel pain and feels comfortable. After the stimulation, the n eedle is removed and the needle site is sterilized.

###### No-treatment control group

We do not offer acupuncture or electroacupuncture.

- - 1. End of study

After 5 weeks of acupuncture treatment, adverse events and adherence will be assessed, including visual analog scale (VAS), body temperature (thermometer measurement), quality of life assessment questionnaire (WHOQOL-BREF), quality of life assessment (EQ-5D), coldness diagnostic questionnaire, vital signs, weight measurement, laboratory tests, medical history and medication changes, and blinded maintenance survey.

- - 1. Tracking

Four weeks after the end of the study, participants will be evaluated for adverse events, as needed, and checked for changes in visual analog scale (VAS), body temperature (thermometer measurement), quality of life questionnaire (WHOQOL-BREF), quality of life assessment EQ-5D, coldness diagnostic questionnaire, vital signs, weight, medical history, and medication history.

##### Combination Therapy and Cautions

- - 1. In principle, medications and other therapies other than acupuncture should not be administered during the trial period.
    2. You should not take any additional medications during the study period, including below.

Example) Anticoagulants, psychotropic medications, antidepressants, medications related to hyperthyroidism, dietary supplements (such as red ginseng), OTC blood flow enhancers, and other medications that may affect hand and foot colds.

- - 1. However, if necessary, this may be done at the discretion of the investigator based on the following criteria. A participant will be dropped from the study if, in the opinion of the investigator, the medications and therapies administered may affect the endpoints of the study. (See exclusion criteria).

Example) Anticoagulants, psychotropic medications, antidepressants, medications related to hyperthyroidism, dietary supplements (such as red ginseng), OTC blood flow enhancers, and other medications that may affect hand and foot colds.

- - 1. All medications administered and the reason for administration must be documented on the CRF and signed by the investigator.

**Clinical trial flowchart**

| Recruitment and Informed Consent (Visit 0) |
| --- |

↓

| Screening |
| --- |
| Vital signs, height, weight, pathways to and motivation for study participation, demographic and sociodemographic surveys, obstetrical Informational history, medical history and medication history, general physical examination, thermometer measurement, VAS of coldhypersensitivity in the hands and feet, pattern identification questionnaire, laboratory test, Chest X-ray, ECG, Determination of exclusion criteria |

↓

| Randomization |
| --- |
| Acupucnture treatment group, Electroacupuncture treatment group, Control group |

↓

| Baseline (0week) Evaluation (Visit 1) |
| --- |
| Vital signs, weight, medical history and medication history and changes, constitutional diagnostics questionnaire (KS-15), general physical examination, thermometer measurement, VAS of coldhypersensitivity in the hands and feet, quality of life assessment questionnaire (WHOQOL-BREF), quality of life assessment EQ-5D, coldness diagnostic questionnaire, treatment, scheduling next visit |

↓

| Acupuncture or Electroacupuncture treatment (Visit 2~9) |
| --- |
| Acupuncture or electroacupuncture treatment for 5 weeks, vital signs, medical history and medication history and changes, general physical examination, thermometer measurement, VAS of coldhypersensitivity in the hands and feet, checking for adverse events, laboratory tests, scheduling next visit |

| Week 5 Evaluation (Visit 10) |
| --- |
| Vital signs, weight, medical history and medication history and changes, general physical examination, thermometer measurement, VAS of coldhypersensitivity in the hands and feet, quality of life assessment questionnaire (WHOQOL-BREF), quality of life assessment EQ-5D, coldness diagnostic questionnaire, checking for adverse events, laboratory tests, scheduling next visit |

↓

| Daily routine for 4 weeks |
| --- |

↓

| Week 9 Evaluation (Visit 11) |
| --- |
| Vital signs, weight, medical history and medication history and changes, general physical examination, thermometer measurement, VAS of coldhypersensitivity in the hands and feet, quality of life assessment questionnaire (WHOQOL-BREF), quality of life assessment EQ-5D, coldness diagnostic questionnaire, checking for adverse events |

↓

| Data cleaning and statistical processing |
| --- |

↓

#### Observation and inspection items

|  | | Screening | Treatment period | | | |
| --- | --- | --- | --- | --- | --- | --- |
| Visit | | (D -7~0) | Visit 1  (D 1) | Visits 2-9 | Visits 10  (D 35±3) | Visit11  (D 63±3) |
| Obtain written consent | | ● |  |  |  |  |
| Assign a screening number | | ● |  |  |  |  |
| Chest X-ray & EKG^8)^ | | ● |  |  |  |  |
| Inclusion/exclusion criteria^9)^ | | ● |  |  |  |  |
| Randomization | |  | ● |  |  |  |
| No-treatment control group | |  | ■ |  | ■ | ■ |
| Assess treatment adherence | |  |  | ○ | ○ | ○ |
| Whether participants drop out of the study | |  |  | ○ | ○ | ○ |
| Vital signs | | ● | ● | ○ | ● | ● |
| Height, weight, and body mass index (BMI) | | ● |  |  | ●  (Weight only) | ●  (Weight only) |
| Pathways and motivations to participate in  research | | ● |  |  |  |  |
| Demographic, sociodemographic, gynecologic and obstetric information^10)^ | | ● |  |  |  |  |
| Investigate medical and medication  history^11)^ and identify changes | | ● | ● | ○ | ● | ● |
| Constitutional Diagnosis Questionnaire (KS-15) | | ● |  |  |  |  |
| Inspection | |  |  |  |  |  |
|  | General physical exam | ● | ● | ○ | ● | ● |
|  | Thermometer measurement^12)^ | ● | ● | ○ | ● | ● |
|  | VAS of coldhypersensitivity in the hands and feet | ● | ● | ○^13)^ | ● | ● |
|  | Screening for and identifying  adverse events |  |  | ○ | ● | ● |
|  | Pattern identification questionnaire | ● |  |  |  | ● |
|  | Coldness diagnostic questionnaire |  | ● |  | ● | ● |
|  | Quality of life assessment  questionnaire (WHOQOL-BREF) |  | ● |  | ● | ● |
|  | Quality of life assessment EQ-5D |  | ● |  | ● | ● |
| Laboratory test | | ●^14)^ |  |  | ○ |  |
| Acupuncture or electroacupuncture treatment | |  | ○ | ○ | ○ |  |
| Specify next visit date | |  | ● | ○ | ● |  |
| Examiner signature | | ● | ● | ● | ● | ● |

● Conduct all three groups

○ Acupuncture treatment group, electroacupuncture treatment group

■ No-treatment control group

1. Chest x-ray and electrocardiogram performed during screening
2. Performed at screening
3. Age, occupation, digestion, exercise, smoking, drinking, sleep, and motivation to participate
4. Include a medical and medication history related to coldhypersensitivity
5. Bilateral temperature measurements of the anterior thigh (ST32), dorsum of the foot (LR3), palm (PC8) and arm (LU4) at each visit
6. The acupuncture group and the electroacupuncture group additionally VAS of coldhypersensitivity in the hands and feet at Visit 4 and Visit 8
7. Screening includes hematologic tests (WBC, RBC, Hemoglobin, platelet), blood chemistry tests (BUN, creatinine, AST, ALT, r-GTP, glucose), thyroid function tests (free T4, TSH), urine and urinalysis, and pregnancy test (Urine HCG). At D35 (Visit 10), hematology (WBC, RBC, Hg, platelet), blood chemistry (BUN, creatinine, AST, ALT, r-GTP, glucose) will be performed

##### Visit 0 (Screening)

- 1. **Recruiting research participants**

The recruitment of volunteers is conducted through two primary avenues: walk-in patients at the site and open recruitment through outdoor advertising. Volunteers are selected through a competitive recruitment process.

##### Consent and assigning participant identification codes

- - 1. The principal investigator or research associate will fully explain the study to participants in the IRB approved informed consent document and privacy statement.
    2. The research participants are fully explained and given time to think about whether or not to consent to the study, and if they agree, they sign the consent form in their own handwriting with their name and date of consent. The principal investigator or research associate in charge of explaining the consent also writes the date, name, and signature.

※ Note

- The principal investigator or research associate must verify that the signature, name, and date of the participant's consent form match before providing a copy of the consent form.
- If the participant makes a mistake and tries to correct the consent form, the first step is to rewrite it on a new form. Otherwise, write one line in black, followed by a comment, the date of the correction, and your name.

The use of overwrites or modifications is absolutely prohibited.

a. How to make a correction: Draw a line and write "Reason/Correction Date/Signature (or initials)”.

Mislabeling 08-03-24 JBH

146~~/86~~ 88

- Ensure that the date of the consent form is the same as the date of the visit in the medical record, and that the date of the consent form can never be later than the date of the actual assignment of the participant identification code in the clinical trial.
  - 1. Give the consent form and one copy of the consent form to the participant and obtain a handwritten "I have received a copy of the consent form" and the date, name, and signature of the participant.
    2. Assign a participant identification code for screening.

##
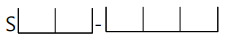


① S: Screening

② The first two spaces

: When recruiting research participants at Semyung university korean medical hospital at Chungju, capitalize SC for each space.

## S

S C

③ The abbreviations for each sites are as follows

- Semyung university korean medical hospital at Chungju (SC)
- Sangji university korean medical hospital (SJ)
- Dongguk university korean medical hospital at Ilsan (DG)

④ Hyphen followed by three spaces

Number each visit according to the order in which you signed the consent form.

Example) First: 001

Example) The screening number of a participant who signed the 15th informed consent at Semyung university korean medical hospital at Chungju is as follows


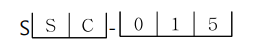


##### Audience selection

If the person wants to participate in the study, explain the study in detail, have them sign a voluntary written consent form, and give them a screening number.

- - 1. Record vital signs, height, and weight

Vital signs a re assessed at every visit. Vital signs measure blood pressure, pulse rate, and temperature.

- - 1. Demographic and sociographic information

Age, Occupation, Digestion, Exercise, Smoking, Drinking, Alcohol, Sleep

- - 1. Pathways and motivations to participate in research
    2. Gynecology and obstetrics information

Menstrual History

Age at menarche, average menstrual cycle, average menstrual duration, presence or absence of lumps during menstruation, color of menstruation, presence or absence of dysmenorrhea

Leukorrhea

Presence, and color

Pregnancy

Number of past pregnancies, fertility status, lower abdominal coldness

- - 1. Coldhypersensitivity in the hands and feet/other body system medical history and medication history
       - Medical history for coldhypersensitivity in the hands and feet: Conducted at Visit 1

Items related to coldhypersensitivity in the hands and feet (if applicable)

- - - - - Whether or not you've been diagnosed with coldhypersensitivity in the hands and feet
        - Duration of coldhypersensitivity in the hands and feet (months)
        - Previous treatment for coldhypersensitivity in the hands and feet

(medication/ herbal treatment (acupuncture/ moxibustion/ herbal medicine, etc.)/ exercise therapy/ psychotherapy/ other complementary medications)

- - - - - Medications and treatments taking for coldhypersensitivity in the hands and feet
    1. Other organ-specific medical history items- at Visit 1

General physical exam items: at each visit

Obtain a medical history including sensory, musculoskeletal, endocrine, multiple system, urinary, reproductive, digestive, cardiovascular, neurologic, dermatologic, psychiatric, hematologic and lymphatic, and respiratory systems. Any significant findings on examination at the first visit will be recorded in the other body system related fields of the case record sheet, and any significant findings on medical examination that meet the definition of an adverse event after initiation of the study procedure will be recorded on the adverse event case record sheet.

- - 1. Thermometer measurements

In a room at 24±2°C, and 40-60% humidity, expose at least 10cm above both elbows, sit and rest for 10 minutes with lower extremity clothing up to the knee, and take three consecutive measures using a non-contact infrared thermometer at a distance of approximately 3cm from the anterior thigh (ST32), dorsum of the foot (LR3), palm (PC8), and arm (LU4), and record the median value, excluding the highest and lowest values.

- - 1. Hands and Feet Coldness (Visual Analog Scale: VAS)

The VAS of coldhypersensitivity in the hands and feet will be completed by the participants with the assistance of the researcher. On the VAS, participants will mark an “I” for their level of hands and feet coldness.

O, V, not verbalized (to be precise)


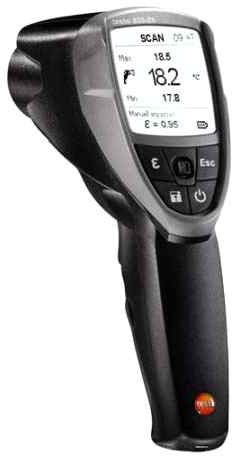

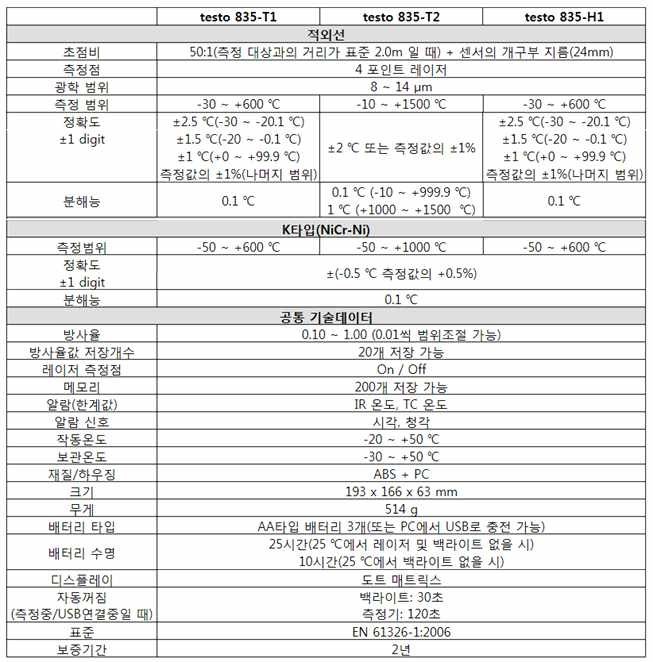
Using a horizontal straight line labeled with numbers from 0 to 10 before the start of the acupuncture treatment and after the end of the treatment, have the participants select the number that corresponds to their level of fatigue. At the left end of the straight line, add the description 'no cold' and at the right end, 'the most severe cold imaginable'. The score is the number they choose.

Only those who have a temperature difference of 0.3℃ or more between the palm (PC8) and arm (LU4) or a temperature difference of 2℃ or more between the anterior thigh (ST32) and dorsum of foot (LR3) measured by a thermometer at the time of the screening visit and a Visual Analogue Scale (VAS) of coldhyepersensitivity in the hands or feet of 4 or more will be enrolled.

- - 1. Pattern identification questionnaire

At Visit 0, the principal investigator or research associate will evaluate participants.

1. The evaluator checks the most appropriate condition for the participants and ◯ marks.
2. The evaluator marks the corresponding pattern identification with a ◯.
   - 1. Constitutional Assessment Questionnaire (KS-15)

Conducted by the principal investigator or research associate at Visit 0

- - 1. Laboratory tests

At visit 0, a 12-lead EKG, chest x-ray, and the following tests are performed

① Hematologic tests: WBC, RBC, Hemoglobin, Platelet;

② Blood biochemistry test: BUN, creatinine, AST, ALT, r-GTP, glucose

③ Urine and urine specimen tests: Blood, Urobilinogen, Bilirubin, Ketone, Protein, Nitrate, Glucose, pH, S.G, LEU, WBC, RBC

④ Pregnancy test (women of childbearing age): Urine HCG;

⑤ Thyroid function tests: free T4, TSH

- - 1. Determining conformance to inclusion/exclusion criteria

At Visit 0, consent, demographic and sociodemographic information, medical history, laboratory tests, pregnancy test and chest x-ray, electrocardiogram, and thermometer measurements to determine if the participant meets the coldhypersensitivity diagnostic criteria will be performed. Results will be combined to make a final determination of eligibility and participants will be contacted by phone within 14 days of testing to schedule Visit 1.

##### B. Visit 1 (D1)

1. Vital signs and weight measurement
2. Verify medical conditions/medications/treatments (check for changes in medical history and medications)

From Screening to Visit 1, investigate whether any new conditions, additional medications, or additional treatments have occurred. If yes, record in the Conditions/Medications/Treatments CRF form.

1. General physical exam

Determine if the patient has any of the conditions listed on the general physical examination form in the CRF at the time of the visit.

If present, circle yes and record in the abnormal findings section below.

V for none.

1. Thermometer measurements

At each visit, before and after treatment, bilateral temperatures at the anterior thigh (ST32) and dorsum of the foot (LR3), and at palm (PC8) and arm (LU4) will be measured using a non-contact infrared thermometer at a distance of, 3cm for three consecutive measurements and the median value excluding the highest and lowest values will be recorded.

1. Hands and feet coldness Visual Analogue Scale (VAS)

The VAS of coldhypersensitivity in the hands and feet will be completed by the participants with the assistance of the researcher. On the VAS, participants will mark an I for their level of hands and feet coldness.

O, V, not verbalized (to be precise)

1. Coldness diagnostic Questionnaire

Let the paticipants fill in the fields that need to be filled in.

The measurement must be performed by a practitioner.

1. The Korean version of the World Health Organization Quality of Life Scale (Short Form) (WHOQOL-BREF) and the EQ-5D Quality of Life Assessment

Check ◯ only for the most appropriate number.

Paper medical records keep the questionnaire and, if the medical record is electronic, the score, description, or item number. or itemized numbers. If this is difficult, create and store a clinical trial-specific paper medical record file.

1. Randomization

Randomize to no treatment, acupuncture treatment, and electroacupuncture treatment.

Record the predetermined randomization table number in the medical record and CRF.

1. Acupuncture or electroacupuncture

An investigator who does not participate in the survey will administer the treatment to the randomized acupuncture group or electroacupuncture group, respectively.

1. Specify your next visit date

In the acupuncture treatment group and the electroacupuncture treatment group, the next visit will be scheduled within 4±3 days after the treatment, based on the principle of twice-weekly treatment. For the no-treatment control group, the next visit will be scheduled on day 35±3 (visit 10) without any treatment.

##### C. Visit 2 ~ Visit 9

1. Participants dropouts

If a participant violates the protocol for any reason, discontinue participation in the study and record it in the End of Study field. If there is no reason for the violation, the clinical trial proceeds.

Withdrawal from a clinical study is based on the following criteria. Participant will be excluded from the study if any of the following conditions apply, as determined by researcher.

a. Participants will be dropped if predicted treatment adherence is less than 70%. Failure to visit within ±3 days of the scheduled visit will be considered a missed visit.

b. Became pregnant during the study

c. If surgery or hospitalization is required due to a disaster such as an accident or the onset of other diseases

d. In case of refusal by a clinical trial participant

e. Prohibited concomitant medications and therapies.

Example) Anticoagulants, psychotropic medications, antidepressants, medications related to hyperthyroidism, dietary supplements (such as red ginseng), OTC blood flow enhancers, and other medications that may affect coldness in the hands and feet.

f. Worsening of coldness in the hands and feet symptoms requiring standard treatment.

f. Serious adverse events that make it unreasonable to continue in the study.

g. Other unavoidable reasons have arisen that make it impossible to continue the study or that the principal investigator believes would have affected the results of the study.

1. Measure vital signs

Measure and record vital signs.

For vital signs, check and record body temperature, systolic blood pressure, diastolic blood pressure, and pulse rate.

1. Verify medical conditions/medications/treatments (check for changes in medical history and medications)

Check for any new medical conditions, additional medications, or other treatments during Visits 2 through 9.

If so, record it in the Condition/Medical Supplies/Treatment CRF form.

1. General physical exam

Determine if the participant has any of the conditions listed on the general physical examination form in the CRF at the time of the visit.

If present, circle yes and record in the abnormal findings section below.

V for none.

1. Acupuncture or electroacupuncture

A researcher who did not participate in the survey will administer the appropriate treatment to the randomized acupuncture or electroacupuncture arm.

1. Thermometer measurements

At each visit, before and after treatment, bilateral temperatures at the anterior thigh (ST32) and dorsum of the foot (LR3), and at the palm (PC8) and arm (LU4) will be measured using a non-contact infrared thermometer at a distance of 3 cm for three consecutive readings, and the median value excluding the highest and lowest readings will be recorded.

1. Visual Analogue Scale (VAS) of coldhypersensitivity in the hands and feet

The VAS of coldhypersensitivity in the hands and feet will be completed by the participant with the assistance of the researcher. On the VAS, participants will mark an I for their level of hands and feet coldness.

O, V, not verbalized (to be precise)

1. Screening for and identifying adverse events

Check for any adverse events that occurred during the acupuncture treatment.

If there are any adverse events, complete an adverse event report in the CRF.

Adverse event reporting procedures are in accordance with the adverse event reporting SOP and the standard operating procedures for acupuncture treatment administration.

1. Specify your next visit date

Notify the study box of the date of the next visit, which should be within 4±3 days from the previous visit, based on the principle of twice-weekly treatments.

##### D. Visit 10 (D35±3)

1. Participant dropouts

Calculate treatment adherence for participants.

Example) Treatment Adherence (%) = Participant's actual acupuncture treatments / 10 acupuncture treatments

Adherence to acupuncture treatment: □□.□ %

1. Vital signs and weight measurement

Measure and record vital signs and weight.

For vital signs, check and record body temperature, systolic blood pressure, diastolic blood pressure, and pulse rate.

1. Verify conditions/medications/treatments (check for changes in medical history and medication adherence)

Check for any new conditions, additional medications, or other treatments since visit 9. If so, record in the Condition/Medications/Treatments CRF form

1. General physical exam

Determine if the particiapant has any of the conditions listed on the general physical examination form in the CRF at the time of the visit. If present, circle yes and record in the abnormal findings section below.

V for none.

1. Acupuncture or electroacupuncture

A researcher who did not participate in the survey will administer the appropriate treatment to the randomized acupuncture or electroacupuncture arm.

1. Thermometer measurements

Before and after treatment, bilateral temperatures at the anterior thigh (ST32) and dorsum of the foot (LR3), and palm (PC8) and arm (LU4) will be measured using a non-contact infrared thermometer at a distance of 3cm for three consecutive readings, and the median value excluding the highest and lowest values will be recorded.

1. Visual Analogue Scale (VAS) of coldhypersensitivity in the hands and feet

The VAS of coldhypersensitivity in the hands and feet will be completed by the participant with the assistance of the researcher. On the VAS, participants will mark an I for their level of hands and feet coldness.

O, V, not verbalized (to be precise)

1. Screening for and identifying adverse events

Check for any adverse events that occurred during the study. If so, create an adverse event report in the CRF.

Adverse event reporting procedures are in accordance with the adverse event reporting SOP and the standard operating procedures for acupuncture treatment administration.

1. The Korean version of the World Health Organization Quality of Life Scale (Short Form) (WHOQOL-BREF) and the EQ-5D Quality of Life Assessment

Use ◯ only for the most appropriate number.

In the paper medical record, keep the questionnaire and, if it is an electronic medical record, record the score, description, or item number. If this is difficult, create and store a paper medical record file for your study.

1. Coldness Diagnostic Questionnaire

Let the participant fill in the fields that need to be filled in.

The measurement must be performed by a practitioner.

1. Laboratory tests

General hematology tests for WBC, RBC, Hemoglobin, and Platelets.

Blood chemistry tests for BUN, Creatinine, AST, ALT, and r-GTP.

It will be performed in the laboratory of each participating medical center.

The korean medical hospital will refer the above tests to the laboratory of a western medical institution for testing.

1. Specify your next visit date

Remind participants to schedule their next visit within 4 weeks ± 7 days.

##### E. Visit 11 (D63±7)

1. Vital signs and weight measurement

Measure and record vital signs and weight.

For vital signs, check and record body temperature, systolic blood pressure, diastolic blood pressure, and pulse rate.

1. Verify conditions/medications/treatments (check for changes in medical history and medication adherence)

Check for any new medical conditions, additional medications, or other treatments during the period from Visit 10 to Visit 11.

If so, record it in the Condition/Medical Supplies/Treatment CRF form.

1. General physical exam

Determine if the participant has any of the conditions listed on the general physical examination form in the CRF at the time of the visit. If present, circle yes and record in the abnormal findings section below.

V for none.

1. Thermometer measurements

At each visit, bilateral temperatures at the anterior thigh (ST32) and dorsum of the foot (LR3), and palm (PC8) and arm (LU4) will be measured using a non-contact infrared thermometer at a distance of 3cm for three consecutive readings, and the median value excluding the highest and lowest readings will be recorded.

1. Visual Analogue Scale (VAS) of coldhypersensitivity in the hands and feet

The VAS of coldhypersensitivity in the hands and feet will be completed by the participant with the assistance of the researcher. On the VAS, participants will mark an I for their level of hands and feet coldness.

O, V, not verbalized (to be precise)

1. Screening for and identifying adverse events

Check for any adverse events that occurred during the four weeks. If there are any, create an adverse event report in the CRF.

Adverse event reporting procedures are in accordance with the adverse event reporting SOP and the standard operating procedures for acupuncture treatment administration.

1. The Korean version of the World Health Organization Quality of Life Scale (Short Form) (WHOQOL-BREF) and the EQ-5D Quality of Life Assessment

Check ◯ only for the most appropriate number.

In the paper medical record, keep the questionnaire and, if it is an electronic medical record, record the score, description, or item number. If this is difficult, create and store a paper medical record file for your study.

1. Coldness Diagnostic Questionnaire

Let the participant fill in the fields that need to be filled in. The measurement must be performed by a practitioner.

##### F. End of Study

1. V for Yes if the trial is finalized and record the end date.

Example) Was the trial completed in accordance with the protocol?

□ Yes → End date 20□□Year □□Month □□Day

1. If you dropped out of the study midway through or lost compliance, V-Check No in the CRF form [Study Terminated], complete the date of the dropout or withdrawal, and V-Check the reason for the dropout or withdrawal.

Example) □ No → Suspension or dropout date20□□Year □□Month □□Day

Causes of study discontinuation or dropout

- - Violation of Inclusion/Exclusion Criteria
  - Unidentified systemic disease before clinical trial
  - Adverse reaction
  - Intercurrent illness (not intervention related)
  - Participant's request for withdrawal (other than adverse reaction)
  - Investigator indicated termination
  - Exclusion from study due to non-adherence to protocol
  - Pursuit failure
  - Other other ( )

1. Make any notes in the progress or overall comments.
2. The principal investigator must sign and date the CRF after finalizing it.

##### G. Complete CRF and finalize case closure signature

1. Once the CRF is finalized, the principal investigator checks that it matches the supporting documentation (medical records) and identifies any discrepancies, missing records, or missing CRF checks.
2. Check for omissions through monitoring, etc.
3. Verify that the supporting documentation matches the CRF and that the consent form is signed, dated, and signed with no misspellings.
4. The investigator and principal investigator finalize the completion of the CRF by signing the case conclusion to assure compliance with the case record.

#### Predicted Adverse Events and Precautions for Use of Acupuncture

##### Predicted adverse events

Acupuncture and electroacupuncture are generally considered to have the potential to cause adverse reactions, including redness, swelling, edema, pain, and purpura, based on what is known to occur.

##### Precautions for use

Precautions for acupuncture and electroacupuncture include below

Pay attention to hygiene when storing acupuncture treatments

Be mindful of hygiene and infection when needling

Stop if patient complains of severe pain

#### Study discontinued, study participants Exclusion Criteria

Particiapants will be excluded from the study if any of the following conditions apply, as determined by researcher.

##### Particiapants will be dropped from the study if the predicted treatment adherence is less than 70% at the end of the study. Failure to visit within ±3 days of the scheduled visit will be considered a missed visit.

##### Became pregnant during the study

##### If surgery or hospitalization is required due to a disaster such as an accident or the onset of other diseases

##### In case of refusal by a clinical trial participant

##### Prohibited concomitant medications and therapies.

Example) Anticoagulants, psychotropic medications, antidepressants, medications related to hyperthyroidism, dietary supplements (such as red ginseng), OTC blood flow enhancers, and other medications that may affect coldhypersensitivity in the hands and feet.

##### Worsening of coldhypersensitivity in the hands or feet requiring standard treatment.

##### Serious adverse events that make it unreasonable to continue in the study.

##### Other unavoidable reasons have arisen that make it impossible to continue the study or that the principal investigator believes would have affected the outcome of the study.

For those who drop out, we will recommend conventional medical treatment of coldhypersensitivity in the hands and feet if the participant wants additional treatment, or standardized treatment if the participant wants maintenance treatment. The duration of treatment for these dropped particiapnts and the issue of compensation with or without treatment will be determined by the institutional review board according to the condition of the dropped participants and the causal relationship with acupuncture treatment.

#### Criteria for evaluating safety, how it is evaluated, and how it is reported to

##### Predictable or Unpredictable Adverse Events

An adverse event is an undesirable, unintended event, symptom, or illness that occurs during the course of a clinical trial and is not necessarily causally related to the intervention.

Predictable adverse events

The types of adverse reactions that can occur with body or electroacupuncture include below

- Localized abnormalities

Subcutaneous hemorrhage and hemorrhagic bullae formation

Peripheral neuritis/ Cellulitis

Localized tingling sensation

Allergic reaction to needle cramps or burning needles

Pain at the needle site (only if it lasts longer than 2 weeks)

- Autonomic and central nervous system abnormalities

Drop in blood pressure and fainting/excessive sweating Drowsiness (only if lasting more than 1 week)

Confusion (only if lasting more than 1 day) / Coma

- Gastrointestinal abnormalities Nausea/vomiting
- Mental issues

Anxiety, fear (only if it lasts for more than 60 hours per incident) Drowsiness

- Other

Headache (only if it lasts more than 3 days)

Hypersensitivity with tingling symptoms (only if lasting more than 3 days)

Attacks after needle insertion

Unclear language

Unpredictable adverse events

A discrepancy in the modality or degree of harm in light of available information about body acupuncture and electroacupuncture (e.g., acupuncture practice guidelines, acupuncture textbooks, relevant literature).

Cautions for use

If a participant complains of excessive fatigue or declining health on the day of a scheduled acupuncture or electroacupuncture treatment, or if the study researcher examines the participant via diagnosis and determines that the participant's condition is not suitable for treatment, the treatment date can be rescheduled within 3 days before or after the scheduled date to avoid potential adverse events associated with treatment.

##### B. What is evaluated for safety

The safety evaluation includes all participants who were randomized and participated in the trial.

##### C. Safety assessment

- Blood tests: blood tests are performed before and after treatment to check for adverse events.
- Cardiovascular system: Blood pressure and pulse rate are measured at each visit.

##### D. Observe adverse events

Participants are trained to voluntarily report information about any adverse events that occurred during the study period, and the investigators verify the presence of adverse events through laboratory tests and questionnaires.

The investigator should evaluate adverse events and record any previously unrecorded adverse events in the appropriate section of the case record, indicating the action taken, the nature of the event, date and time of onset, duration, severity, and relation to acupuncture or electroacupuncture. Any changes in the frequency or severity of adverse events during the study should be recorded on a separate page.

1. Assessing the severity of an adverse event

Based on clinical signs and symptoms of an adverse event, the criteria are as follows

| 1 = Mild | Does not interfere with normal daily life (functioning) to the extent that it is barely noticeable to the study participant.  Mostly not requiring treatment. |
| --- | --- |
| 2 = Moderate | The degree to which the participant is uncomfortable and interferes with normal daily activities (functioning).  The degree to which the particiapant may be able to continue the study but may need treatment. |
| 3 = Severe | The degree to which the participant is so uncomfortable that daily activities (functioning) are not possible and continued participation in the study is not possible.  The extent to which treatment or hospitalization may be required |

1. Evaluating acupuncture and causality

The degree of certainty that an adverse event can be attributed to acupuncture or electroacupuncture (or to other causes, such as the natural history of the underlying condition or concomitant therapies) is determined by how well the adverse event fits the following criteria: the adverse event appeared after starting acupuncture, disappeared after discontinuation, or reappeared upon reuse, indicating that it is related to acupuncture treatment.

1 = Definitely relevant Certain > 90%

2 = Highly relevant Probable/likely > 70%

3 = Possibly related Possible 50%

4 = Not thought to be relevant Unlikely < 30%

5 = Difficult to determine relevance Conditional/ unclassified < 10%

1. Serious adverse events

"Serious adverse event" means an event that meets 1 of the following criteria

A) Causes death or is life-threatening;

B) Requires hospitalization or extended hospitalization.

C) Causes persistent or significant disfigurement or impairment

D) Causes birth defects or abnormalities

Adverse events should also be reported as serious if they are deemed serious by the investigator or if they suggest a significant risk, contraindication, adverse effect, or caution associated with acupuncture or electroacupuncture. Participants who experience an adverse event should be followed by the investigator until the adverse event resolves or stabilizes.

1. Actions taken regarding adverse events

0 = No action taken

1 = Temporary suspension

2 = Stop acupuncture or electroacupuncture interventions

3 = Medications

4 = Non-drug treatments

5 = Extended hospitalization/stay

**E. Reporting**

The principal investigator will educate co-investigators and participants about any adverse reactions that may occur after acupuncture or electroacupuncture interventions and will educate them to report any phenomena that occur after the intervention.

All systemic or pathologic symptoms that occur after acupuncture or electroacupuncture interventions shall be recorded and kept in a case record sheet that conforms to the standards of clinical trial management, including the type, time of occurrence, severity, treatment, therapeutic agent, outcome, and causal relationship to acupuncture treatment.

If a "serious adverse event" occurs during the study, it will be reported to the Institutional Review Board (IRB) to determine whether the study should be continued or discontinued.

1. If participants die during the study or pose a serious risk to life, this will be reported within 7 days of receiving the report, but in this case, further details will be reported within 8 days of the initial report.
2. For all other serious and unexpected adverse events, report them within 15 days of the date the sponsor is informed or becomes aware of them.
3. Additional safety information related to the reporting of the above should be reported by phone or in-person by the investigator until 7 days after the end of the study (washout period).
4. The principal investigator shall conduct all aspects of the study in accordance with the Declaration of Helsinki.

□ Women of childbearing age

①Women of childbearing potential must have a negative urine pregnancy test prior to study entry.

②Women of childbearing potential must be using adequate and reliable contraception for at least 1 month prior to study entry and continue for the duration of the study.

③If you think you may become pregnant during the study, notify the study staff immediately, and the pregnancy test may be repeated.

##### F. What to do if an adverse event occurs

During this study, the investigator and personnel in charge of the study must ensure the safety of the participants, and in the event of a serious adverse event, the study must be stopped and prompt and appropriate measures must be taken to minimize the adverse event.

In the event of a "serious adverse event" during a clinical trial, each person's responsibilities are as follows

1. Principal investigator duties

The principal investigator should immediately report any serious adverse events that occur during the clinical trial to the institutional review board and sponsor and stop all or part of the clinical trial for that acupuncture and electroacupuncture treatment until otherwise instructed.

1. Investigator duties

The clinical investigator shall immediately report any serious adverse events that occur during the conduct of the clinical trial to the clinical investigator in charge and the sponsor.

1. Institutional review board duties

In the event of a serious adverse event, the IRB shall take any necessary action, including ordering the discontinuation of part or all of the study, to the principal investigator.

#### How to evaluate validity and how to interpret

##### Primary outcome variable

- - 1. Visual Analogue Scale (VAS) of coldhypersensitivity in the hands and feet:
- At each visit, a 10 cm long line is drawn and participants are asked to select the number on the line that corresponds to their level of coldness. For the hand visual analog scale, the left end of the straight line is labeled "no hand coldness" and the right end is labeled "most severe hand coldness"; for the feet visual analog scale, the left end of the straight line is labeled "no feet coldness" and the right end is labeled "most severe feet coldness". The score is the number of ruler points at the location of the 'I' in the big box.
- Examine the difference between pre-treatment and post-treatment results for VAS outcomes assessed at each visit, by visit date.

[References]

Ryoo GS, et al. A case report of cold hypersensitivity caused by acute and chronic blood deficiency. The journal of oriental obstetrics & gynecology. 2009;22(2):222-30.

Lee MJ, et al. A clinical case study to evaluate the hot flush and abdominal cold hypersensitivity relief efficacy of Cheonglijagamtang in climacteric women by DITI. Journal of oriental medical thermology. 2010;8(1):26-32.

Cho JY, et al. A case report of cold hypersensitivity of hands and feet patient. The journal of oriental obstetrics & gynecology. 2011;24(3):195-202.

Lee YJ, et al. Effects of far-infrared rediating products on coldhypersensitivity of lower limbs using cold stress test (CST): a pilot study. Journal of oriental medical thermology. 2008;6(1):69-75.

- Visual Analogue Scale (VAS)

Please place an 'I' in the graph below for your average daily hand coldness over the past week

No hand coldness

Most severe hand coldness

0 points 10 points

- Visual Analogue Scale (VAS)Score:

Please place an 'I' in the graph below for your average daily feet coldness over the past week.

No feet coldness

Most severe feet coldness

0 points 10 points

##### Score:

- 1. **Secondary outcome variable**
     1. Body temperature: Thermometer measurement
- The difference in temperature between the bilateral palm (PC8), arm (LU4), anterior thigh (ST32), and dorsum of foot (LR3) regions, measured within 10 minutes of each visit, before the start of treatment and after the end of treatment, respectively, using a thermometer


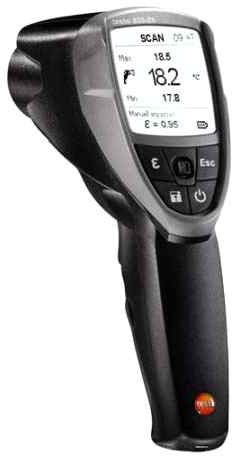

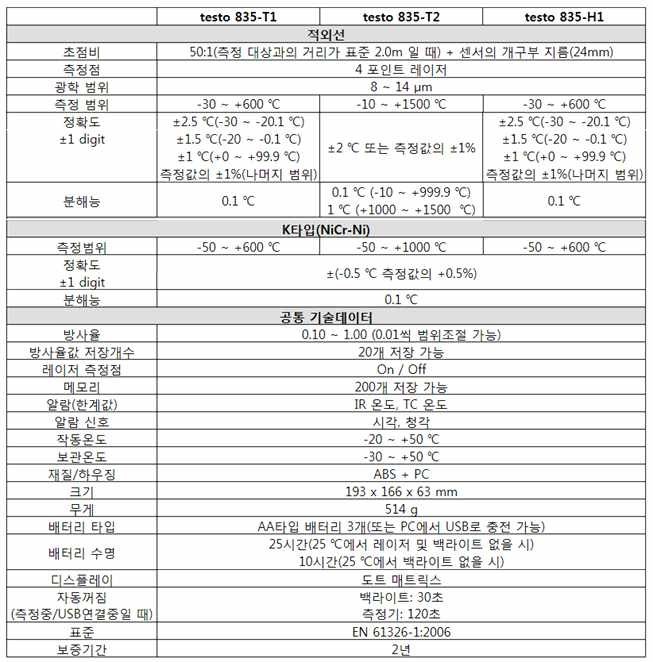


[References]

Lee MJ, et al. A clinical case study to evaluate the hot flush and abdominal cold hypersensitivity relief efficacy of Cheonglijagamtang in climacteric women by DITI. Journal of oriental medical thermology. 2010;8(1):26-32.

Kim EG, et al. A clinical case study for the diagnosis and assessment of abdominal cold hypersensitivity treatment by DITI. Journal of oriental medical thermology. 2009:7(1):55-65.

Cho JY, et al. A case report of cold hypersensitivity of hands and feet patient. The journal of oriental obstetrics & gynecology. 2011;24(3):195-202.

Lee YJ, et al. Effects of far-infrared rediating products on coldhypersensitivity of lower limbs using cold stress test (CST): a pilot study. Journal of oriental medical thermology. 2008;6(1):69-75.

- - 1. Quality of Life Assessment Questionnaire (WHOQOL-BREF) and Quality of Life Assessment EQ-5D
- Overall quality of life, general health, physical health, psychological health, social relationships, and environment, measured at visits 1, 10, and 11.
  - 1. Pattern Identification Questionnaire
- Pattern identification questionnaire measured at visit 1 to correlate symptoms and VAS changes in patients with cold feet according to dialectic indicators.
  - 1. Coldness Diagnostic Questionnaire
- Coldness Diagnostic Questionnaire, which asks participants about their subjective or objective symptoms of coldness, is measured at Visit 1, Visit 10, and Visit 11.

#### Statistical Methods

##### General principles

The basic analysis method is the degree of change between the results of the baseline test performed before the clinical trial and the results of the test performed at the end of the clinical trial for the efficacy evaluation indicators, and the significance level is 0.05 by two-sided test.

For the efficacy portion of the study, all data from study participants will be analysed using both an intention-to-treat analysis (ITT), in which all participants who met the inclusion/exclusion criteria and who voluntarily agreed to participate in the human study and were randomized regardless of protocol violations or dropouts, and a per-protocol analysis (PP), in which all participants who were randomized will be included in the analysis, excluding participants with noncompliance or missing data. Missing observations for efficacy endpoints will be analysed using the last observation carried forward (LOCF) method, and missing observations for safety endpoints will be treated as missing.

As assigned analyses include all participants who have completed the baseline and final examinations, with the last examination data considered the final examination data. The safety analysis methodology will also follow the as assigned analysis methodology. As-treated analyses will include patients who completed the final examination with no major protocol violations.

The results of the two analyses should theoretically agree within the same study, but if there is an induced difference between the results, a plausible explanation for the difference should be provided.

For statistical analyses other than those specified in this protocol, please refer to the statistical guidelines for clinical trials.

##### Compare baseline demographic and assessment variables

To test whether the distribution of variables in the treatment and control groups is homogeneous, analysis of variance (ANOVA) or nonparametric methods are used for continuous variables, and Chi-square test is used for categorical variables.

##### How to analyze validity variables

- - 1. Primary efficacy endpoint: Visual Analogue Scale (VAS) coldhypersensitivity in the hands and feet

The primary efficacy evaluation variables will be analyzed using the ITT group as the main analysis group, and the results of the PP group will be presented in addition. Repeated ANOVA, paired t-test, etc. will be performed to test whether there is a difference in the change in VAS score between the no-treatment control group and the acupuncture treatment group or the electroacupuncture treatment group. The comparison of the degree of improvement of VAS scores within each group will be performed by paired t-test using the difference between pre- and post- treatment VAS scores (VAS score at Weeks 5±3 days - VAS score at Visit 1).

- - 1. Secondary Validation Variables
       1. Body Temperature (BT; thermometer measurement): Repeated ANOVA is performed to test whether there is a difference in BT change between the acupuncture treatment group and the electroacupuncture treatment group. To compare the degree of improvement in BT within each group, a paired t-test will be performed using the difference between BT before and after acupuncture treatment (BT at Weeks 5±3 days - BT at Visit 1).
       2. WHOQOL-BREF Quality of Life Assessment Questionnaire: ANOVA and post hoc tests will be used to test the difference between each group. Comparison within each group is done by paired t-test using the difference in values before and after acupuncture treatment (value at Weeks 5±3 days - value at Visit 1).
       3. Pattern Identification Questionnaire:
          - This figure shows the distribution of the dialectical observables (dialectical indicators) in participants with coldhypersensitivity in the hands and feet. To determine the relationship between the dialectical indicators and the change in symptom VAS, an ANOVA test (non-parametric test if not normally distributed) and post hoc tests are performed for each indicator with the change in symptom VAS as the dependent variable.
          - Validity and reliability of the basic instrument are evaluated. The validity evaluation is

a. Construct validity: The construct validity is evaluated by conducting a factor analysis of the items of the basic dialectic tool. After obtaining the scores of each item of the basic argumentation tool, the correlation matrix between the items is obtained. Determine the number of factors by the eigen value of the factor, rotate the factor after checking the construct validity, and interpret and name the items with large factor loadings related to the rotated factor based on the content of the items. b. Predictive validity: Evaluate the correlation between the values of the visual analog scale and the scores of the factors explaining cold feet. It estimates the degree of correlation between the visual analogue scale values of participants with coldhypersensitivity in the hands and feet and the sum of the scores of items that can well explain coldhypersensitivity in the hands and feet. Cronbach's alpha was measured to evaluate the questionnaire. In addition, we will analyze the changes i n the items and the magnitude of each item before and after the treatment, and the magnitude of the treatment effect for each specific type of excuse. In addition, we will use cluster analysis or factor analysis to identify the association and excuse relevance of excuse items related to coldhypersensitivity in the hands and feet.

- - - 1. Coldness Diagnostic Questionnaire:
         - Cronbach's alpha is used to analyze reliability. We also use the statistical method of Item-Total Correlation to analyze the correlation between items by measuring the Pearson correlation coefficient between the total scores of the remaining items.
         - Perform additional cluster analysis and factor analysis.
      2. Quality of life assessment EQ-5D: The EQ-5D index score does not have an officially established cutoff value to assess impaired health-related quality of life, but it is utilized for economic evaluation using the 4th and 5th quintiles.

##### Analysis of safety endpoints

- - 1. Clinical laboratory test data will be analysed using appropriate statistical methods, including within-group comparisons before and after treatment, depending on the nature of the variable, and the frequency, incidence, list, detailed time of occurrence, severity, and causal relationship to acupuncture treatment will be reported in graphical form, if necessary. The statistical analysis will be performed by ANOVA, t-test, chi-square or Fisher's exact test, depending on the nature of the variable.
    2. Adverse events: Table all adverse events, categorized by treatment group and body system, with detailed descriptions. For each group, record the frequency of adverse events that are causally related to acupuncture treatment and those that are not causally related. Determine the number of AEs and the proportion of participants experiencing at least one AE within each arm with 95% confidence intervals and compare between arms.
    3. Clinical pathology: Descriptive statistics will be presented for each group and visit for continuous data such as hematology and blood biochemistry test results and vital signs, and differences between visits will be analysed using paired t-test or non-parametric methods.

##### E. Analyzing missing values

1. In the statistical analysis, the validity endpoint is analyzed using the Last Observation Carried Forward (LOCF) method, which substitutes the measurements from the previous equation for any observations that are not observed for any reason, i.e., missing values. For safety evaluation variables, missing values are treated as missing and analyzed.

#### Handling of adherence and protocol violations in clinical trials

- 1. The principal investigator and personnel in charge of this study must be fully familiar with and thoroughly implement the protocol to prevent violations of the protocol from occurring. On the other hand, any unavoidable violation of the protocol shall be handled as follows.
  2. In the case of serious violations, e.g., violation of inclusion/exclusion criteria, failure to obtain informed consent, etc. that may have a significant impact on the safety and outcome of the study, the participant shall be dropped out of t h e study.
  3. For other minor violations, accurately describe the extent of the violation or delay and the reason for it, and consider whether it affected the pilot study when analysing the results.

#### Resource Management

The principal investigator will manage the data from this study in a manner that maintains objectivity, safety, and complementarity, in accordance with the procedures below. Upon completion of this study, data specific to this study will be kept in a separate file for 3 years.

##### A. Check case notes and raw data

The Case Record Form (CRF) should be filled out as soon as the data to be recorded occurs. If it is not recorded by the end of the case, the appropriate reason for the missing data should be recorded. All amendments should be marked by crossing out the original record and recording the amendment, the amender, the reason for the amendment, and the date of the amendment. Do not use a redaction that obscures the original record.

The CRFs of the patients whose case records have been completed are delivered to the computerized data entry personnel at the race track clinical trial site for entry into the computerized system through the source document verification process as stipulated in the SOP of the organization to which the separate monitoring personnel belong. After all data are entered into the computerized system, the source documents and CRFs shall be kept so that they can be verified upon request by relevant government agencies, sponsors, etc.

The investigator should provide normal ranges and reference values in an appropriate location, such as the CRF, before the trial begins so that the computerized record can be used for verification or validation.

#### Informed consent form

In conducting this study, after fully explaining the contents of this study, the effect of electroacupuncture treatment, acupuncture treatment and adverse events to the participants in advance, the participant's consent is obtained and the consent form is completed, and the date of obtaining consent is recorded in the case record.

#### Protocol for victim compensation

Attachments

#### Standards of care and treatment for post-trial research participants (proposal)

Seek medical attention for adverse events requiring treatment or for participants requiring ongoing treatment.

#### Measures to protect the safety of human participants and etc.

- 1. Pre-treatment testing will be performed to strictly evaluate whether the participant is appropriate for this study.
  2. For any adverse event that appears to be related to the study, the principal investigator shall immediately notify the Institutional Review Board and follow its instructions. However, in cases requiring emergency medical treatment, it is the responsibility of the principal investigator to reprot after preliminary treatment.
  3. Consider all possibilities that may involve a risk of physical injury (including any side effects that affect the body, such as rashes, infections, etc.).
  4. If a participant receives medical treatment for physical injuries that occur as a direct result of participant's participation in the study (and that are not normally expected in the ordinary course of medical care), the participant will not pay for the treatment.
  5. The medical expenses for physical damage caused by the research commissioned by Sangji University Industry-Academic Cooperation Center shall be paid by the insurance company subscribed for this research unless the researcher is grossly negligent due to intentional or negligent acts.
  6. During the study, participants will be treated with traditional chinese medicine for coldhypersensitivity in the hands and feet if their symptom worsen. Since there is no standardized treatment for coldhypersensitivity in the hands and feet, the treatment will be based on the existing treatment methods in korean medicine.

#### Other matters necessary to conduct clinical trials safely and scientifically

##### Korea Good Clinical Practice (KGCP)

In conducting this clinical trial, the fundamental spirit of the KGCP and the Helsinki Declaration will be followed to ensure that the study is conducted with ethical and scientific consideration. Anything not presented in this protocol will be in accordance with the KGCP, the KFDA notification, and the Helsinki Declaration.

##### Human Participants Consent

The investigator explains the nature, scope, and expected outcomes of the study to the participant in advance in a way that the participant can easily understand, and the consent form is signed by the investigator and the participant.

##### Confidentiality

All participants’ names must be kept confidential. The signed consent forms are kept by the principal investigator. The principal investigator should maintain a list with the participant’s number and name so that records can be retrieved at a later date.

##### D. Clinical Trial Monitor

1. Monitoring is conducted to protect the rights and welfare of research participants, to ensure that reported clinical trial-related data are accurate, complete, and verifiable against supporting documentation, and to ensure that clinical trials are conducted in accordance with the provisions of the approved protocol, Good Clinical Practice, and implementing regulations.
2. The study monitor will monitor the study by telephone, e-mail, or in person as needed. The study monitors and investigators will review the progress of all studies and check all records in the case notes. They will also verify that the study is being conducted according to the protocol and communicate and coordinate with the investigator about any problems that arise.
3. The time of the visit should be arranged in consultation with the principal investigator and the study monitor.
4. The principal investigator must also make available to the monitor access to the patient's source documents (source documents: hospital or personal charts, lab result records, appointment records, etc.

#### References

1. Lee SR, et al. A review of the literature on gynecologic coldness. The journal of oriental obstetircs & gynecology. 1996;9(1):55-80
2. Modern Herbal Medicine. Hansung Publishing House. 1989
3. Lee NH, et al. Research of relationship on cold hypersensitivity for the patients in OB & GY OF Dong Eui medical center. The journal of oriental obstetircs & gynecology. 2002;15(2):101-13
4. Cho GH. Guidebook to the practice of oriental and western medicine. Seoul: Korean Medicine. 2001, p. 284-6
5. Kim JH, et al. Analysis of studies on Ojeok-san for estblishment of evidence-based medicine. The korean journal of oriental medical prescription.2008;16(2):1-9
6. Cho SW, et al. A study on quality evaluation of Ojeok-san extract powders distributed in Korea. The journal of korean acupuncture & moxibustion society. 2010;27(2):105-113
7. Kim CM, et al. Recognition of family practitioners on cold hands/feet syndrome and Raynaud’s disease. J Korean Acad Fam Med. 2007;28:339-45
8. Park JY, et al. The relationship between coldness and hands and feet and the erythrocyte deformability in stroke patients. Korean journal of oriental internal medicine. 2010;31(3):578-85
9. Han JY, et al. Cold stress test for the diagnosis of cold hypersensitivity on hands. Journal of oriental medical therology. 2003;2(1):17-23
10. Lee KS. A clinical evaluation of DITI and neurometer for the diagnosis of cold hypersensitivity. Journal of oriental medical therology. 2004;3(1):60-66
11. Kim DH, et al. Standardization of diagnosis of coldhypersensitivity of hands and feet by D.I.T.I. The journal of oriental obstetircs & gynecology. 2001;14(2): 120-134
12. Lee MJ, et al. Analysis of heart rate variability in cold hypersensitivity females visiting Gangnam Kyung-Hee korean hospital. The journal of oriental obstetircs & gynecology.2011;24(3):109-115
13. Yoon SW, et al. Clinical research about the correlation between defecation type and cold hypersensitivity of lower abdomen, hand and foot. The journal of oriental obstetircs & gynecology.2004;17(2):130-7
14. Park KS, et al. A study on digital infrared thermographic imaging characters of women suffering from postpartum disease. The journal of oriental obstetircs & gynecology. 2010;23(2):116-123
15. Kim HW, et al. Correlation between women infertility and DITI. Journal of oriental medical therology.2002;1(1):52-56
16. Park KS, et al. Efficacy and safety of Korean red ginseng for cold hypersensitivity in the hands and feet: A randomized, double-blind, placebo-controlled trial. Journal o f Ethnopharmacology. 2014;158:25-32
17. Ryoo GS, et al. A case of report of cold hypersensitivity caused by acute and chronic blood deficiencycoldness. The journal of oriental obstetircs & gynecology. 2009;22(2):222-30
18. Lee MJ, et al. A clinical case study to evaluate the hot flush and abdominal cold hypersensitivity relief efficacy of Cheonglijagamtang in climacteric women by DITI. Journal of oriental medical therology. 2010;8(1):26-32
19. Cho JY, et al. A case report of cold hypersensitivity of hands and feet patient. The journal of oriental obstetrics & gynecology. 2011;24(3):195-202
20. Ha HY, et al. Effect and safety of oxygen chamber therapy on cold hypersensitivity: a randomized, controlled trial. The journal of oriental obstetircs & gynecology. 2013;26(4):123-39
21. Lee YJ, et al. Effects of far-infrared rediating products on coldhypersensitivity of lower limbs using cold stress test (CST): a pilot study. Journal of oriental medical thermology. 2008;6(1):69-75
22. Nishida S, et al. Effects of a traditional herbal medicine on peripheral blood flow in women experiencing peripheral coldness: a randomized controlled trial. BMC Complement Altern Med. 2015;15:105. doi: 10.1186/s12906-015-0617-4
23. Zhing X, et al. Clinical observation on the treatment of sensory disorders in peripheral neuritis with warm needle moxibustion. Xinjiang journal of traditional chinese medicine. 2007;25(6):22
24. Jeon SW, et al. A review of acupuncture for the treatment of Raynaud’s disease. Journal of internal korean medicine. 2017;38(4):433-42

[Attachment 1] Protocol for Victim Compensation

[Attachment 1] Protocol for Victim Compensation

Protocols for victim compensation

1. Study Title: Multicenter clinical study of the efficacy and safety of electroacupuncture for cold hypersensitivity in the hands and feet - Randomized, controlled clinical trials –
2. Principles

①The insurance company contracted with this study will cover any physical harm (including death) to the research participant.

②The insurance company contracted with the study shall compensate the study participant if the cause of the damage is due to the conduct of the acupuncture treatment in the study.

③Only compensate for damage that is serious enough to be persistent and disabling, not for temporary pain or damage that is easily treated.

④If there are side effects caused by acupuncture treatment in clinical research or damage caused by the process of treating side effects, we will compensate for the damage directly caused by acupuncture treatment in clinical research.

⑤If any harm is caused to the participants as a result of this study, it will be compensated through the insurance purchased by the principal investigator.

1. We will not compensate if

①Damage caused by adverse events resulting from treatments or medications not administered or provided under the auspices of this study

②Compensation for the failure of an investigational acupuncture treatment to provide effectiveness or benefit for the indication for which it was given

③Damage caused by failure to comply with a mutually agreed upon protocol

④Damage caused by the negligence of the research participant or his/her guardian

1. Compensation evaluator

① The level of compensation should be appropriate to the nature, extent, and duration of the damage, and should be the same as that generally awarded for similar damage by Korean courts.

②If there is a disagreement between the research participant and the principal investigator about the level of compensation, expert advice acceptable to both parties should be sought.

As the principal investigator of this study, Dr. Jun-Sang Yu, a doctor of korean medicine affiliated with Sangji University, has obtained clinical compensation insurance for this study, and pledges to take care that the research participants do not suffer any disadvantages from this study by keeping the aforementioned provisions in mind, and that he will be held liable under the Victim Compensation Act if any problems arise from this study.

Date: 30/12/2018

Investigator affiliation: Sangji university korean medical hospital


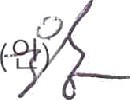
Signature: Professor Jun-Sang

[Attachment 2] Helsinki Delcation

**Helsinki Declaration**

# - Ethical principles for medical research involving human participants

###### Preface

- 1. The World Medical Assembly, through the Declaration of Helsinki, has set forth ethical principles to guide physicians and researchers involved in medical research using human participants. Medical research involving human participants includes research on human samples or materials that can be positively identified.
  2. It is the duty of a physician to promote and protect the health of mankind. A physician's knowledge and conscience should be devoted to fulfilling this duty.
  3. The Declaration of Geneva of the World Medical Congress calls on physicians to "regard the health of the patient as my first concern," and the International Convention on Medical Ethics declares that "when administering treatments that may weaken the patient's physical or mental condition, the physician shall act solely in the patient's interest.
  4. Medical advances are ultimately based in part on experiments with human participants.
  5. In medical research involving human participants, consideration for the welfare o f research participants must be prioritized over scientific and societal interests.
  6. The primary purpose of medical research using the human body is to improve prevention, diagnosis, and treatment, and to advance our understanding of the causes and development of disease. Even well-known preventive, diagnostic, and therapeutic measures should be participant to ongoing research into their effectiveness, efficacy, utility, and quality.
  7. Most of the healthcare and medical research being done today involves a number of risks and burdens in terms of prevention, diagnosis, and treatment.
  8. Medical research must meet ethical standards that promote respect for all humanity and protect human health and rights. Some experimental groups may be at risk and require special protection. Be aware of the special needs of research participants who are economically or medically disadvantaged. Special attention should be paid to research participants who may not have the capacity to grant or refuse consent on their own, or who may have consented under coercion, or who have no personal benefit from the research, or where research and treatment are combined.
  9. Researchers should be aware of the ethical and legal requirements and regulations in their own countries, in addition to international requirements, when conducting research involving human participants. However, no national ethical or legal requirements or regulations can diminish or eliminate the protections provided in this statement for human participants.

###### Basic principles for all medical research

1. In medical research, it is a physician's duty to protect the life, health, privacy, and dignity of research participants.
2. Medical research involving human participants should be based on generally accepted scientific principles, be well informed by the scientific literature and other relevant information, and be supported by adequate experimental data and, where possible, results from animal studies.
3. Due care must be taken when conducting research that may have environmental impacts, and the welfare of animals used in experiments must be considered.
4. The planning and conduct of each experimental procedure involving human participants must be clearly documented in a research protocol. This protocol must be submitted to a specially constituted Institutional Review Board for review, advice, guidance, or, if necessary, approval. This committee must be independent of the investigator, sponsor, or any other party with the power to exert undue influence of any kind. This independent committee must follow the laws and regulations of the country in which the trial is being conducted and has the right to investigate how the trial is being conducted. The investigator is obligated to report information to the committee, especially any serious adverse events. Investigators must also report to the committee for review any potential conflicts of interest with funding, sponsors, affiliated organizations, and other interests, as well as any potential conflicts of interest with respect to participant pay.
5. The protocol should always include a statement that ethical considerations were considered and that the principles set out in the Declaration of Helsinki were followed.
6. Biomedical testing using human participants may only be conducted by qualified scientists under the supervision of a competent clinician. The responsibility for research involving human participants rests with the qualified medical practitioner and never with the participant, even if the participant has given consent.
7. All medical research involving human participants must be carefully weighed against all foreseeable benefits, risks, and burdens to the research participants and others before proceeding. However, this does not preclude healthy volunteers from participating in medical research. All protocols must be publicly available.
8. A physician should only undertake a test that involves human participants if he or she is confident that the risks have been adequately reviewed and can be adequately managed. If the risks are judged to outweigh the potential benefits, or if there is conclusive evidence of a positive and beneficial outcome, the test should be stopped.
9. Medical research involving human participants should only be conducted when the importance of the research outweighs the risks and burdens to the human participants. This is especially important when the research participants are healthy volunteers.
10. Medical testing can only be justified if there is a reasonable likelihood that the group on whom the testing is conducted will benefit as a result of the testing.
11. Participants must be volunteers and know they are participating in a study.
12. The rights of human participants to protect their own safety must be respected. Every care must be taken to protect the privacy of the research participants, to ensure the confidentiality of their personal information, and to reduce the physical and psychological trauma and personality effects of the research.
13. For research involving human participants, the participants must be fully informed in advance about the purpose and methods of the research, t h e source of funding, any possible conflicts of interest, the institutional affiliation of the investigator, the anticipated benefits and inherent risks, and the risks of suffering. They should also be informed that they are free to stop participating in the study at any time without penalty and that they are free to withdraw their consent at any time. Once the physician has verified that the participant understands everything, the physician should obtain the participant's freely given, documented consent, if possible. If documented consent cannot be obtained, verbal consent should be obtained in the presence of witnesses and formalized in writing.
14. When obtaining consent to conduct research, physicians should pay particular attention to whether the relationship is not one in which the participant has any expectations of the physician or whether the consent is given under any circumstances of coercion. If this is the case, consent should be obtained by a physician who is not participating in the research, has no relationship with the participant, and is fully informed about the research.
15. When a research participant i s legally incompetent, physically or mentally incapacitated, unable to give direct consent, or a minor, consent must be obtained from are relative who is legally authorized to act on their behalf. These populations should not be subjected to research unless the research is necessary to promote the health of the population or cannot be performed on legally competent persons.
16. If a person is deemed legally incapacitated, such as a minor, but is actually able to express a willingness to participate in a test, the investigator must obtain the person's consent in addition to the consent of a relative who can act on their behalf.
17. Testing on individuals who are incapable of giving informed consent, including proxy consent and informed consent, should only be conducted if the physical or mental circumstances that prevent them from giving consent are necessary for the experiment. The reasons for testing on individuals who are incapable of giving consent must be described in the protocol submitted for review and approval by the review board. The protocol must also state that consent to continue participation in the study must be obtained from each individual or their legal representative as soon as possible.
18. Both authors and publishers have ethical responsibilities. When publishing research results in magazines, investigators should be impartial. Both positive and negative results should be published or disclosed in the magazine. The source of funding, the organizations involved, and any possible conflicts of interest should also be disclosed in the publication. Tests that violate the principles set forth in this declaration should not be accepted for publication in the magazine.

###### Additional Principles for Medical Research with Therapeutic Applications

1. A physician may only conduct medical research in conjunction with his or her practice to the extent that the research is recognized by the public as helpful in the prevention, diagnosis, and treatment of disease. When conducting medical research as part of a practice, additional rules must be followed to protect patients who are research participants.
2. The benefits, risks, burdens, and effects of adopting a new method should be weighed against the best available prevention, diagnosis, and treatment. This is also true for the use of placebo or no treatment in the absence of known preventive, diagnostic, and therapeutic options.
3. At the conclusion of the trial, all patients who participated in the trial should be able to be confident that they have access to the best prevention, diagnosis, and treatment availablethrough the research.
4. The physician must fully inform the patient of what part of the treatment is related to the study. A patient's refusal to participate in a study should not interfere with the doctor-patient relationship will be.
5. In treating patients, physicians should be free to use unproven or new preventive, diagnostic, and therapeutic measures, with the patient's consent, when known preventive, diagnostic, and therapeutic measures are unavailable or ineffective, if they believe they will help save lives, promote health, and alleviate suffering. Whenever possible, these new methods should be tested and designed to reveal their safety and effectiveness. In all cases, new information should be documented and, if possible, published in papers. Other related guidelines in this manifesto should also be followed.
